# Continental influx and pervasive matrilocality in Iron Age Britain

**DOI:** 10.1038/s41586-024-08409-6

**Published:** 2025-01-15

**Authors:** Lara M. Cassidy, Miles Russell, Martin Smith, Gabrielle Delbarre, Paul Cheetham, Harry Manley, Valeria Mattiangeli, Emily M. Breslin, Iseult Jackson, Maeve McCann, Harry Little, Ciarán G. O’Connor, Beth Heaslip, Daniel Lawson, Phillip Endicott, Daniel G. Bradley

**Affiliations:** 1 Department of Genetics, Trinity College Dublin, Dublin, Ireland; 2 Department of Archaeology and Anthropology, Bournemouth University, Bournemouth, UK; 3 Department of Life and Environmental Sciences, Bournemouth University, Bournemouth, UK; 4 School of Mathematics, University of Bristol, Bristol, UK; 5 Institute of Genomics, University of Tartu, Tartu, Estonia; 6 Department of Linguistics, University of Hawai‘i at Mānoa, Mānoa, HI USA; 7 DFG Center for Advanced Studies, University of Tübingen, Tübingen, Germany; 8 Éco-anthropologie, Musée de l’Homme, Paris, France

**Keywords:** Population genetics, Archaeology, Evolutionary genetics, Social anthropology

## Abstract

Roman writers found the relative empowerment of Celtic women remarkable^1^. In southern Britain, the Late Iron Age Durotriges tribe often buried women with substantial grave goods^2^. Here we analyse 57 ancient genomes from Durotrigian burial sites and find an extended kin group centred around a single maternal lineage, with unrelated (presumably inward migrating) burials being predominantly male. Such a matrilocal pattern is undescribed in European prehistory, but when we compare mitochondrial haplotype variation among European archaeological sites spanning six millennia, British Iron Age cemeteries stand out as having marked reductions in diversity driven by the presence of dominant matrilines. Patterns of haplotype sharing reveal that British Iron Age populations form fine-grained geographical clusters with southern links extending across the channel to the continent. Indeed, whereas most of Britain shows majority genomic continuity from the Early Bronze Age to the Iron Age, this is markedly reduced in a southern coastal core region with persistent cross-channel cultural exchange^3^. This southern core has evidence of population influx in the Middle Bronze Age but also during the Iron Age. This is asynchronous with the rest of the island and points towards a staged, geographically granular absorption of continental influence, possibly including the acquisition of Celtic languages.

## Abstract (web summary)

An analysis of ancient mitochondrial and nuclear DNA shows evidence of matrilocal communities in Iron Age Britain.

## Main

The structure of a society is shaped by the residence patterns of its married couples^4^. Matrilocality, whereby partners predominantly reside with or near the wife’s parents, is relatively rare in modern ethnographic databases^5,6^, whereas patrilocality is by far the most common system. Furthermore, in most European Neolithic, Copper and Bronze Age sites with sufficient genomic and archaeological data, evidence of patrilocality and patriliny has been reported^7–13^.

**Extended Data Fig. 1:**
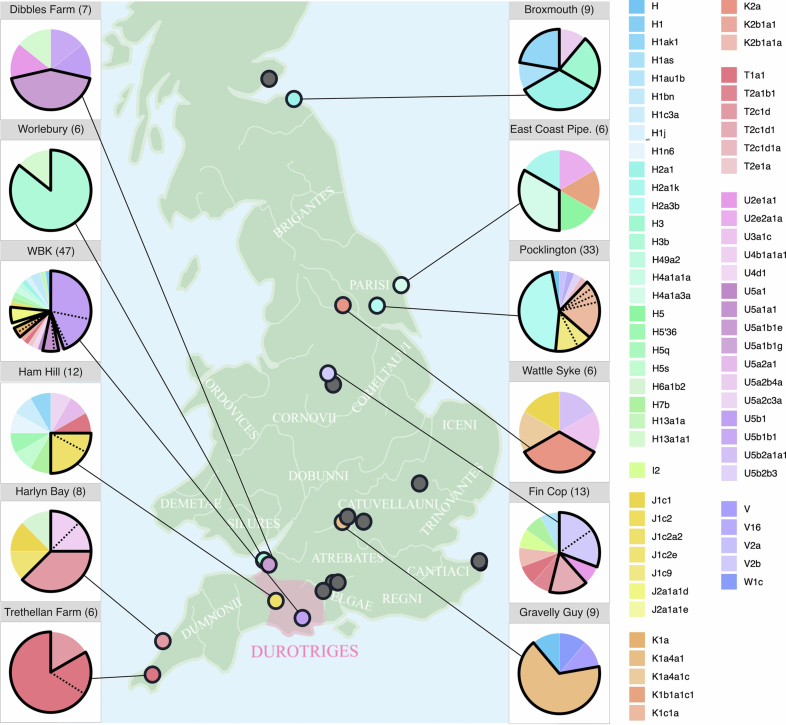
Mitochondrial haplogroup frequencies at British Iron Age sites. Only sites with a sample size of six or higher are considered and first-degree relative pairs are removed. Of the 23 sites considered, 11 have a *h* value of 1 (no haplotype represented more than once). These are marked as black points on the map and are composed almost entirely of genetically unrelated individuals. Pie charts show haplogroup frequencies for the remaining 12 sites. Haplogroups with a count above one are emphasised with a black outline in the pie charts. Dotted lines are used to split haplogroups into downstream subclades based on additional mutations. Iron Age tribe names recorded by classical writers and their approximate geographic locations are shown. The haplotypes of all British Iron Age samples used in this analysis are given in Supplementary Table 12.

Despite being on the cusp of the historical era, little is known about the social structures of the Iron Age peoples of Britain. In the early centuries ad, Ptolemy described the locations of various *ethne* on the island with names of Celtic origin (Extended Data Fig. 1), and Caesar referred to *civitates*. These ambiguous terms are often translated as ‘tribes’, although the complexities of such group identities are not well understood. Interestingly, two of the earliest recorded British rulers were women, Cartimandua and Boudica, suggesting that both sexes could reach the highest political status. From Cartimandua’s 30-year reign of the Brigantes, a tribe covering much of northern England, we learn that women could inherit property, divorce and lead armies to great effect^1^. In the east of England, Boudica of the Iceni famously led an uprising that destroyed three Roman towns and challenged the authority of the imperial government^14^. Furthermore, Julius Caesar noted, in the mid-first century bc, that British women could take multiple husbands (*De Bello Gallico*). However, such social descriptions are seen as suspect, biased towards what would have seemed exotic to a Mediterranean audience that was immersed in a deeply patriarchal world^1^.

**Fig. 1:**
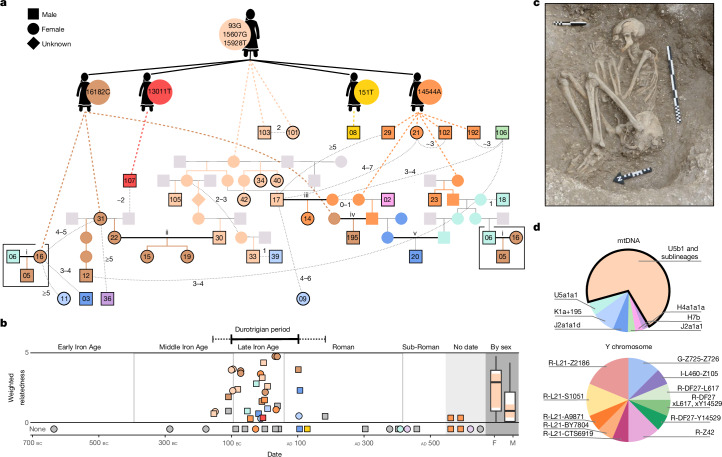
The WBK pedigree. **a**, The best-fitting pedigree (for uncertainties, see Supplementary Note 4). Sampled individuals are outlined in black with WBK ID number and are coloured by mtDNA haplotype. The founding U5b1 + 16189 + @16192 female is shown at the top, with her four descendants with de novo mutations underneath. Further descendants are connected with dashed lines. Matings between descendants of the founding female are shown in bold, labelled i–v. Deduced relationships not fitted on the pedigree are shown with light-grey lines, with the estimated degree of relatedness. **b**, Weighted relatedness of each genome plotted versus the point carbon-14 date estimate (average 95% confidence range: 202 years). For each, the sum of their total number of biological kinship links (seventh degree or less) is shown, inversely weighted by the degree of the relationship. Individuals are coloured by mtDNA haplotype; grey indicates singleton haplogroups. The Durotrigian period (solid line) and the range of dates of family members (dashed line) are indicated. The summed relatedness is also shown in box plots (Tukey) by sex for individuals in the latter range; a significant difference between males (M) and females (F) is observed (Welch’s *t*-test, two-tailed, *P* = 0.029). The frequency of the dominant mtDNA lineage for each group is the proportion of each boxplot body in colour, which was also significantly different (two-tailed Fisher’s exact test, *P* = 0.02). **c**, A flexed inhumation excavated at WBK, typical of the Durotrigian cultural zone (photo credit: Bournemouth University). **d**, mtDNA and Y chromosome haplogroup frequencies for individuals with at least one genetic relative and sufficient Y chromosome coverage (Supplementary Table 9).

The distributions of grave goods in multiple western European Celtic cemeteries have been interpreted as supporting high female status^15^. British archaeological evidence, however, is limited as Iron Age human remains are rare, with individuals perhaps predominantly cremated, excarnated or deposited in wetlands. The Durotriges tribe, who occupied the central southern English coast around 100 bc to ad 100, were one exception, depositing their dead in formal cemeteries of flexed inhumations (Fig. 1c and Supplementary Note 1). Interestingly, it is women who are more commonly associated with a greater number and diversity of prestige items in these burials, hinting at high status and perhaps a matrifocal society^2^.

The genomic variation of Iron Age Britons has been investigated^16–19^, but with limited data from single cemeteries that could clarify social customs relating to kinship and marriage. Genomic survey has contributed to debates on the spread of Celtic languages (Supplementary Note 1.6), with the Middle to Late Bronze Age identified as a candidate window for arrival based on the inference of large-scale migration to the island during this period, followed by substantial genetic isolation in the Iron Age^17^. However, the characterization of gene flow into Britain requires further refinement through haplotypic analysis and regional dissection. Here, we sequence 55 genomes from Durotrigian and other cemeteries at Winterborne Kingston (WBK), Dorset, along with two well-furnished female Durotrigian burials from Maiden Newton and Langton Herring^2,20^ (Supplementary Table 2 and Supplementary Note 1). These reveal a community characterized by female-line descent. When combined with data from other British Iron Age sites, our analyses find that matrilocality is widespread, reveal fine-scale genealogical networks that align with geographical boundaries and show a genomic footprint of Iron Age immigration on the south coast that is reflective of both contemporary Roman writing and archaeological datasets.

## Matrilocality in Durotrigian society

Excavations at WBK in coastal southern England have revealed considerable evidence for settlement, spanning the later Bronze Age, around 1000 bc, to the post-Roman period, around ad 500, including several small Durotrigian-type cemeteries from the later Iron Age^20^ (Fig. 1b and Supplementary Note 1). Genomic data were retrieved for all 55 skeletal samples taken from the site (Supplementary Table 1), with 40 achieving a coverage high enough for genotype imputation and robust identification of genomic segments that were identical by descent (IBD) between individuals^21^ (>0.3×; Methods and Supplementary Note 3). This revealed WBK to be the burial ground of a large kin group during the Durotrigian period of the site’s usage (around 100 bc to ad 100; Fig. 1b), with 30 of 40 individuals possessing at least one relative of approximately the seventh degree or closer (Supplementary Table 10; see Supplementary Note 4.3 for exact criteria). An additional four low-coverage members of this kin group were identified through allele-matching analysis.

Strikingly, more than two thirds (24/34) of the genetically identified kin belong to a rare lineage of mitochondrial haplogroup U5b1 (Fig. 1d) that has not been observed previously in ancient sampling and that has a frequency of only 3 × 10^−5^ in modern data^22^ (Supplementary Table 7 and Supplementary Note 2.4). The predominance of this single matriline is not skewed by an abundance of siblings, with only two pairs of sisters (all adults) observed (Fig. 1a). Additional downstream mutations distinguish four subclades in this haplogroup that are unique to WBK. Using one of the faster estimates of the mitochondrial DNA (mtDNA) mutation rate^23^ (4.72 × 10^−7^ mutations per site per generation), we estimate that at least 420 female births to lineage mothers would be required to result in this level of within-clade diversity (Supplementary Note 2.6), implying a long-term association between this haplotype and WBK. By contrast, we find that Y chromosome diversity is high (Fig. 1d and Supplementary Note 2.8), and runs of homozygosity (ROH) indicate that this was an outbreeding community (Supplementary Note 5.5). Theory, modelling and surveys of modern populations^24,25^ have demonstrated that such patterns are generated by matrilocal customs (that is, male-biased dispersal).

To confirm matrilocality at WBK, we carried out two types of simulation (Supplementary Notes 2.10 and 4.5). First, we modelled different rates of male and female migration between demes in a population and estimated the resulting uniparental haplotype diversity (*h*) (Methods and Supplementary Note 2.2). These simulations indicated an outward female migration rate close to zero and a male rate between 0.15 and 1 per generation. Second, we simulated the distribution of autosomal and X chromosome kinship coefficients in a seven-generation pedigree whose members practised alternately (1) patrilocality, (2) matrilocality or (3) mixed residence. Again, the observed data are consistent with matrilocality (Supplementary Note 4.5). The earliest incidence of the dominant mtDNA lineage is in two second-degree relatives (346 to 51 calibrated (cal) bc), with the last observation in the Roman period (cal ad 31 to 212), when British Celtic societies underwent radical changes (Fig. 1b). Accordingly, the latest family member was buried following a new funerary rite of extended inhumation (WBK36; cal ad 82 to 316).

## Marriage custom in an Iron Age community

We reconstructed the most parsimonious pedigree for the core kin group, which further confirms matrilocal traditions at WBK coupled with male mobility (Fig. 1a and Supplementary Note 4). We found only one patrilineal relationship greater than the first degree (WBK02 and WBK195), and we infer this to involve multiple partnerships with matriline women across generations. An adult woman (WBK31), her daughter (WBK22) and her adult granddaughters (WBK15 and WBK19) are all buried at the site, as well as an inferred matrilineal great-grandson (WBK12) of WBK31 through a different male partner. There is also one unusual case of a double relationship in our pedigree; from IBD segment length distributions, we can conclude that WBK17 is most likely the son of stepchildren whose parents’ marriage produced the sisters WBK34 and WBK40 (Supplementary Note 4.8).

**Extended Data Fig. 2:**
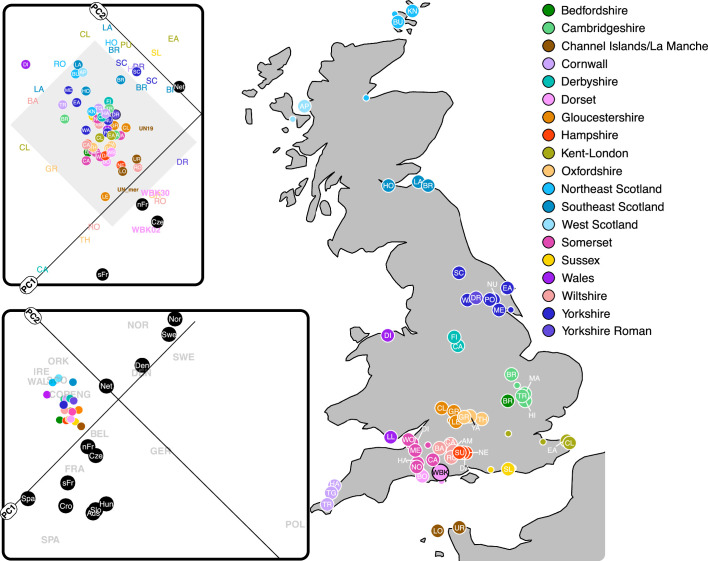
Projection PCA of Iron Age British and European genomes. The bottom panel shows median positions of modern European populations (grey text with first three letters of country), Iron Age continental populations (black circles with first three letters of country) and Iron Age British and coastal French populations (coloured circles). Iron Age continental French genomes have been split into northern (nFr) and southern (sFr) populations. The map provides a colour key for British and coastal French Iron Age populations (Supplementary Table 12). Sites with data for more than one individual are shown as large circles with two letter identifiers. The median values for these multi-individual sites are plotted in the top PCA panel. The grey box delimits the area within two standard deviations of the mean value for the British Iron Age population along PC1 and PC2. Outlying individuals that fall beyond this area are plotted using their two-letter site ID. Two outliers from the current study are plotted using their full IDs (WBK02 and WBK30). Two data points from Urville-Nacqueville^41^ on the Normandy coast are also highlighted (brown). One of these (UN19) places further away from French populations, in agreement with SOURCEFIND results.

**Extended Data Fig. 3:**
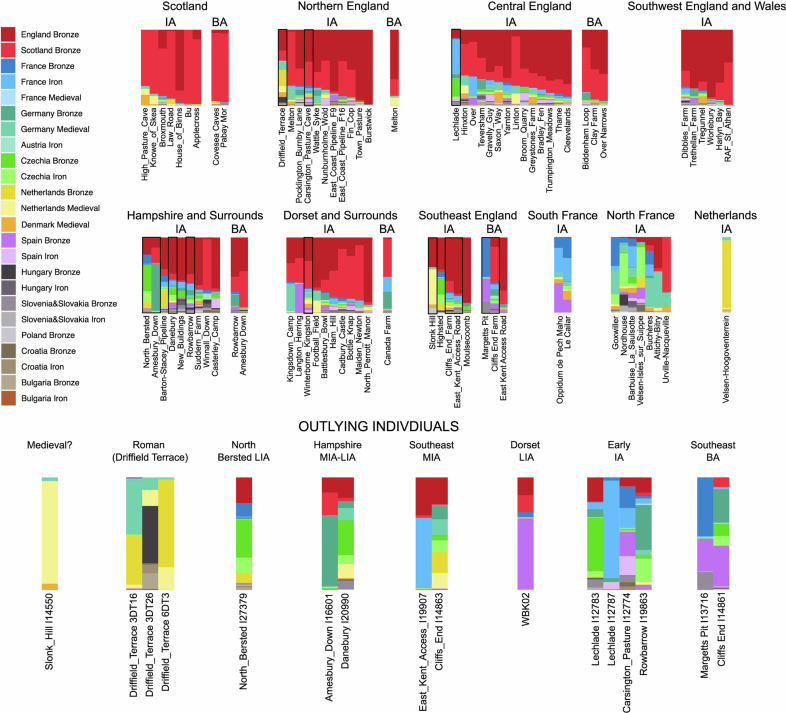
SOURCEFIND ancestry profiles for archaeological sites and outlying individuals. Surrogate and target individuals are listed with their population IDs in Supplementary Table 12. Raw values are available in Supplementary Table 17. The top rows show averaged profiles for Bronze and Iron Age archaeological sites in Britain, France and the Netherlands. British site profiles are outlined in black if the site contains one or more individual outliers (those possessing a level of British Early Bronze Age ancestry two standard deviations below the population mean). The bottom row shows ancestry profiles for these outlying genomes. We note that all Middle and Late Iron Age outliers derive from the channel core region. We identify two previously reported^17^ EEF outliers from Margetts Pit and Cliffs End Farm in Middle-Late Bronze Age Kent, who possess little to no British Early Bronze Age ancestry. (BA: Bronze Age, EIA: Early Iron Age, MIA: Middle Iron Age, LIA: Late Iron Age).

When we consider individuals dating to the Durotrigian period, we find that males show significantly lower levels of genetic relatedness with other individuals and are significantly overrepresented among non-matrilineage individuals (Fig. 1b). Six individuals, all male, show no detectable genetic connection to the WBK kin group (that is, they are not members of the dominant matriline and have no identified relatives), although they may still have been family members (for example, inward-migrating spouses or fostered children). Four of the six who were adult or adolescent at death were buried in typical Durotrigian fashion, three with grave goods comprising locally manufactured ceramic vessels, implying their integration in the community. When considering genetically related individuals, we find eight of ten family members who do not belong to the dominant mitochondrial haplogroup are male. We infer two marriages between these non-lineage men and lineage women, including the outlier WBK02 whose ancestry derives mainly from continental Europe (Extended Data Figs. 2 and 3).

We note that the co-burial of spouses is not typical of a society with strict emphasis on matrilineal descent, in which men will frequently visit or even reside with their matrilineal kin and are often buried alongside them rather than with their wives^26^. Indeed, the integration of husbands into their wives’ households can place strain on matrilineal systems in which nephews inherit from their maternal uncles (the avunculate)^27,28^. For this reason, matrilocality is thought to be more stable when there is less property for male kin to control. It is associated with societies in which wealth is concentrated in the land, which is typically abundant and extensively farmed and owned by women, and in which men are often absent (for example, because of warfare)^27,29,30^.

Interestingly, at WBK we infer five marriages in which both partners descend from the founding female (Fig. 1a), including three in which both members are direct descendants through the female line. However, these partners have no recent relatedness, as indicated by a lack of IBD sharing and lack of ROH in their offspring (Supplementary Note 5.5), and the matriline couples belong to different subclades. This suggests that the people of WBK had a deep knowledge of their own genealogies, which may have been used to guide marital arrangements among a pool of related groups in the local region. These patterns are consistent with modern matrilocal populations^31^ who typically show increased rates of local endogamy (for example, marriages of individuals from nearby villages or within the same village), which can allow men to retain influence in their natal group through geographical proximity.

## Matrilocality across Iron Age Britain

**Fig. 2:**
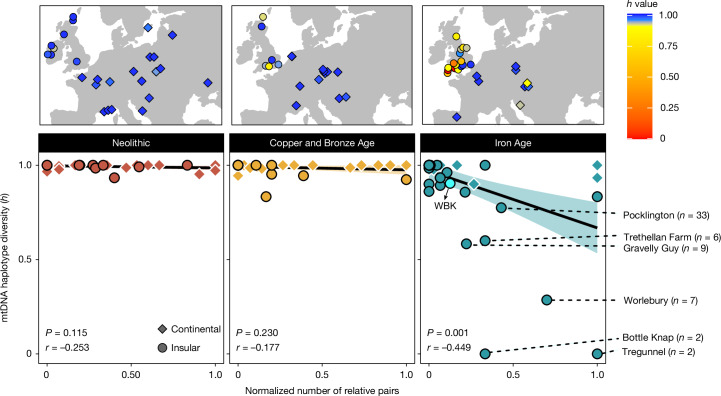
Reduced mitochondrial diversity in British Iron Age communities. Trends in mtDNA haplotype diversity (*h*) for archaeological sites with two or more individuals after pruning of first-degree pairs. Haplotype diversity is calculated as the probability that two randomly selected haplotypes are different (Methods). In the bottom panels, the *h* value is plotted against the normalized number of relative pairs seen for each site (1, all pairs are genetic relatives; 0, no pairs are genetic relatives; Supplementary Note 5.3). The shaded area represents the 95% confidence interval around the fitted line. There is a strong negative correlation between mtDNA diversity and the number of relatives present for Iron Age sites (Pearson correlation coefficient, *P* = 0.001, *r* = −0.449), which is not observed in previous periods of prehistory. When each period is further split into continental and insular (UK and Ireland) individuals (diamonds and circles), we find that the only significant correlation observed is for the British Iron Age (Pearson correlation coefficient, *P* = 5.853 × 10^−7^, *r* = −0.717). The top panels show the geographical distribution of these *h* values for sites with evidence of burial guided by kinship (at least one pair of genetic relatives present). Of the total 156 sites considered, 13 sites are less diverse than WBK: 12 from Britain and 1 from a Celtic La Tène period cemetery (320–180 bc) in Hungary^17^. The sample sizes for the *h* value and normalized relative pair estimation for all sites are presented in Supplementary Table 13.

To place the WBK community in context, we searched for reduced mitochondrial diversity as a signature of matrilocal practice through space and time in Europe (Supplementary Note 2.2 and Supplementary Table 13). We considered 156 archaeological sites (first-degree relatives removed) spanning from the Neolithic to the Iron Age and observed six outlying communities with extremely low levels of diversity (Fig. 2 and Extended Data Fig. 1), all from the English Iron Age: Worlebury (Somerset), Bottle Knap (Dorset), Gravelly Guy (Oxfordshire), Trethellan Farm and Tregunnel (Cornwall) and Pocklington (Yorkshire). We further observed that the 11 lowest diversity estimates come from British Iron Age populations, as well as one English Middle to Late Bronze Age site. By contrast, Y chromosome diversity is high (Supplementary Table 18 and Supplementary Note 2.9), and patterns of ROH imply that these were relatively large outbreeding communities^17^ (Supplementary Note 5.5). At Pocklington^17^, the second-largest British cemetery sample in the dataset, 28 of 33 individuals belong to one of three dominant mtDNA haplogroups, which, in a manner akin to WBK, can be divided into subclades defined by private mutations. Here, the main period of burial activity was between 400 and 50 bc, but the first observation of a dominant matriline pre-dates this in the Early Iron Age (I11033; 717–395 cal bc; Supplementary Table 12).

These results provide strong evidence that longevous matrilocal communities were widespread across the island through the Iron Age and may even have their origins in the preceding Bronze Age period. Analyses of Bell Beaker and Early Bronze Age cemeteries in Britain and Germany have produced evidence of patrilocality and emphasis on patrilineal descent^12,13,32^, which, if reflective of the broader social organization of this period in Britain, raises the interesting possibility of a patrilocal society transitioning to matrilocality. This is a relatively rare occurrence in ethnographic surveys, although these may not be indicative of conditions throughout most of human history^4,33^.

High mitochondrial diversity at a site may not solely reflect residence patterns but can also indicate an overall lack of biological relatedness among individuals; indeed, in Iron Age Britain, mtDNA diversity shows a significant (*P* = 5.85 × 10^−7^) inverse correlation with the normalized number of relative pairs identified using refinedIBD^21^ (Fig. 2). However, no similar reduction in mtDNA diversity is apparent for other prehistoric periods, despite the presence of multiple sites with high levels of biological relatedness (Fig. 2), implying that matrilocal practices were not widespread in Neolithic or Bronze Age Europe. By contrast, when we consider Y chromosome diversity in British Iron Age populations, no correlation with the number of relative pairs is identified (*r* = 0.06, *P* = 0.77; Supplementary Note 2.9).

## IBD segments reveal regional structure

**Extended Data Fig. 4:**
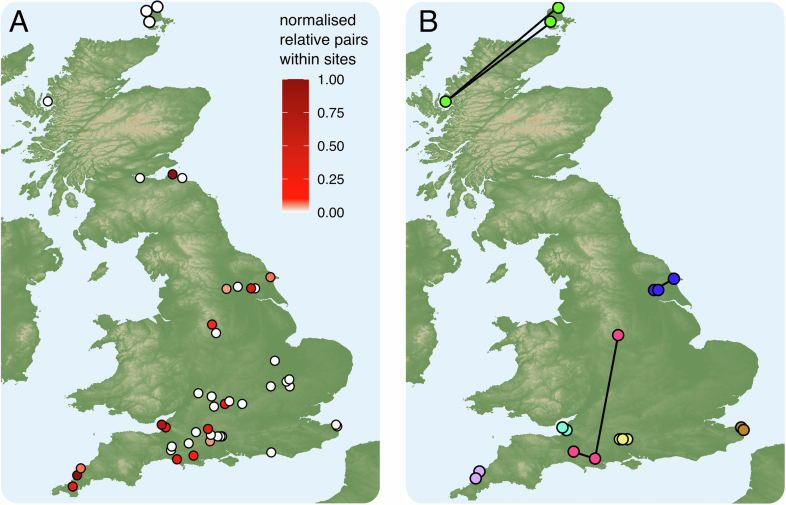
Relatives identified within and between sites in Iron Age Britain based on IBD sharing. Genetic relatives are defined as individuals who share ≥ 24 cM across at least three IBD segments that are 4 cM or longer (Supplementary Note 5.2). **a**, This shows the frequency of relative pairs identified within archaeological sites, where a value of one indicates that all pairs are relatives and a value of zero indicates no pairs are relatives. **b**, This shows archaeological sites that are linked by genetic relatives (Supplementary Table 14; common colours denote a set of linked sites). Most pairs of relatives are identified between neighbouring sites (2–40 km), but several long-distance relative pairs are also identified. An individual from Winterborne Kingston (WBK01) has two relatives at Carsington Pasture Cave in the midlands (red). Three Scottish samples from northern coastal sites also show a relationship (green).

**Extended Data Fig. 5:**
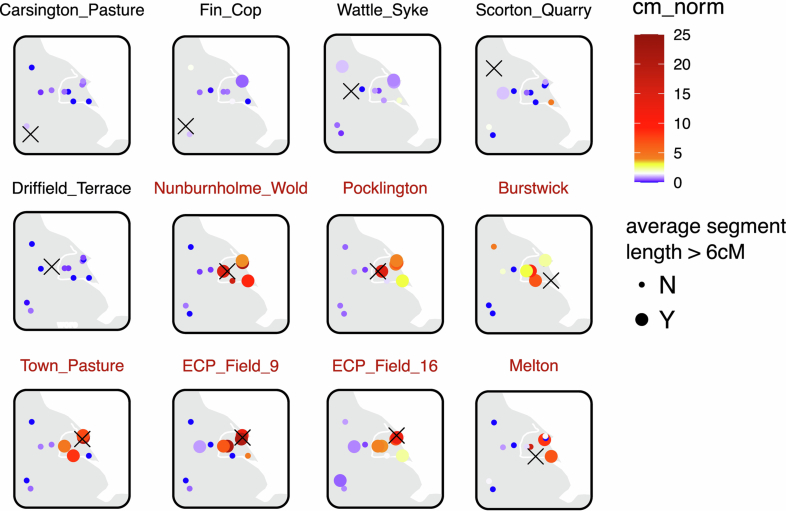
An IBD enclave in East Yorkshire. The normalised amounts of IBD sharing (cM) between 12 sites in northern England. Each panel shows pairwise values for a specific site (marked with an X) and the other 11. Sites highlighted in red text are all located west of the River Derwent (highlighted in white) and show excessive IBD sharing with one another. The Roman burials of Driffield Terrace^18^ show low amounts of IBD sharing with sites across the region, likely due to the individuals buried there being non-local in origin.

We found 30 instances of genetic relatives (more than 24 cM shared) between sites (most of which were between 2 km and 40 km distant), none of whom shared mtDNA haplotypes (Extended Data Fig. 4, Supplementary Note 5.6 and Supplementary Table 14). By contrast, 51% of within-site pairs share their mtDNA. For example, Dibbles Farm and Worlebury Hillfort on the Bristol Channel coast share eight relative pairs (30–55 cM IBD) and each site is dominated by a different matriline (Fig. 2 and Extended Data Fig. 1), suggesting that the movement was of male marriage partners. Similar patterns are seen in East Yorkshire, the region of the distinctive Iron Age Arras Culture associated with the Parisi tribe referenced by Ptolemy. We observe extreme levels of IBD sharing among all sites east of the River Derwent boundary, implying the existence of a cohesive social group in this territory (Extended Data Fig. 5). However, no shared mtDNA haplotype is observed between any of these East Yorkshire sites.

**Fig. 3:**
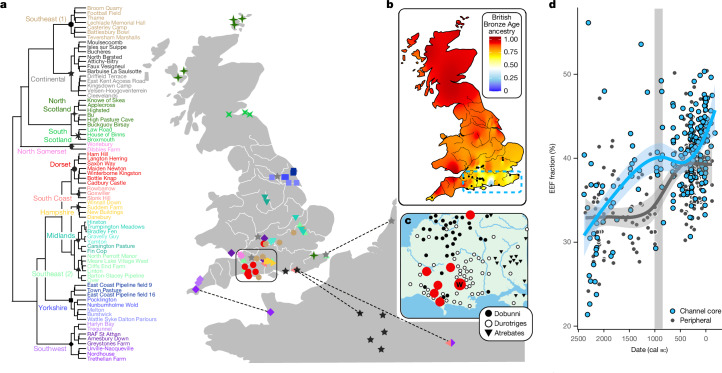
IBD communities in Iron Age Britain show fine-grained geographical structure and include connections across the English Channel. **a**, The clusters are based on the consensus of 100 runs of the Leiden algorithm on a weighted graph of IBD shared between archaeological sites and show geographical integrity. Twelve major clusters (defining nodes marked with symbols) are labelled on the basis of geographical affiliations, with further substructure within clusters emphasized using different colour shades. The cross-channel clusters are highlighted with dashed lines joining nearest geographical neighbours across the channel. **b**, An interpolated map showing the distribution of British Bronze Age ancestry across Iron Age Britain, based on average values generated using ChromoPainter NNLS^38^ and SOURCEFIND^37^ approaches. The lowest values are seen along the south-central coast. Sites with less than 75% contribution are marked in black. **c**, A close-up showing most of the sites from the Dorset cluster (red circles) placed within the regional distribution of Durotriges coin finds. WBK is denoted by ‘W’. The distributions are plotted according to refs. ^34,35^. **d**, The EEF ancestry proportion through time for the channel core region of continental influence (blue; outlined with dashed line in **b**) shows a Late Iron Age increase not observed in the sample from the rest of England and Wales (black). The channel core zone is east of longitude −2.8° (western edge of the Durotrigian zone) and south of latitude 51.5° (River Thames). The period between 1000 and 875 bc (grey rectangle) has been previously associated with an increase in EEF ancestry in southern Britain^17^. This window is populated mostly by high-EEF samples from the channel core, whereas data points directly preceding this window are mostly from the peripheral regions that retained a lower level of EEF ancestry throughout the Middle Bronze Age (Extended Data Fig. 7 and Supplementary Note 6.2).

To further characterize population structure in Iron Age Britain, we carried out Leiden clustering (Methods) on a weighted network graph of IBD sharing between archaeological sites (Fig. 3a and Supplementary Note 5.7). Consensus clusters were identified across 100 independent runs. These show clear geographical patterning; for example, subclusters in Scotland (greens), Yorkshire (blues), the Midlands (aquas) and the southwest (purples) all emerge. WBK is placed within a Dorset cluster (red), which maps onto the known distribution of later Iron Age ‘Durotrigian style’ coinage^34,35^ (Fig. 3c). Interestingly, several clusters encompass both continental and coastal British sites, pointing to cross-channel movements.

**Extended Data Fig. 6:**
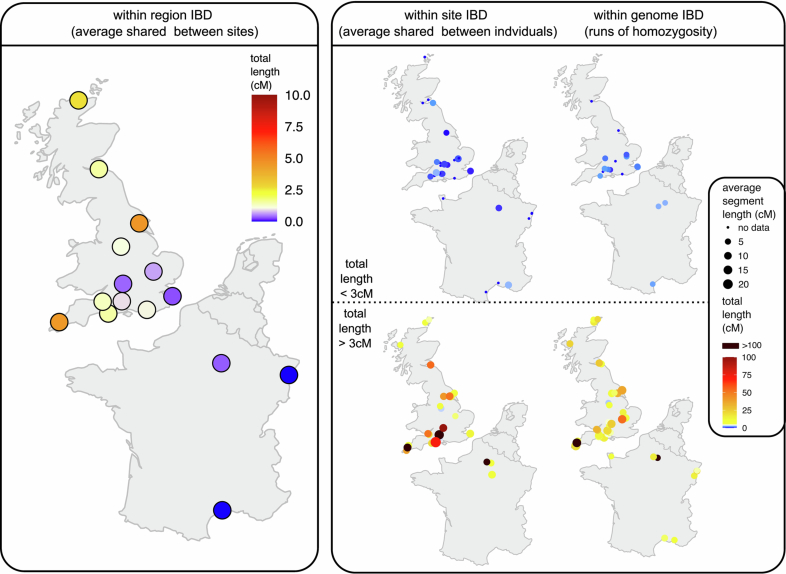
IBD sharing within genomes, sites and regions. For each measure, we calculate the average total amount of IBD shared (cM), represented using a colour scale. For within genome (i.e. runs of homozygosity) and within site comparisons we also show the average size of shared IBD segments. Within region sharing is calculated as the average amount of IBD shared (cM) between sites within a geographic region, defined based on present-day district divisions (see Supplementary Note 5.4, Supplementary Table 12). It is a more robust measure of relative population sizes than within site and within genome IBD sharing, as these latter can be confounded by kinship and cultural practices such as consanguineous marriage. We see lowest values for within region IBD sharing in France and the southeast of England. For within genome IBD sharing, we take the population average of the total length of runs of homozygosity for each archaeological site. If the average total length is above 3 cM we plot the site in the bottom panel. In Britain, sites with reduced runs of homozygosity (top panel) are concentrated in south central and southeastern regions indicative of larger population sizes. Within site IBD sharing (average length shared between individuals in a site) is plotted in a similar fashion.

Patterns of IBD segment sharing also reveal differences in population sizes across Britain and the continent (Extended Data Fig. 6). The south and east of England show markedly reduced levels of ROH and within-region IBD sharing, indicative of higher population densities and connectivity. These were very productive agricultural regions where the first proto-towns (*oppida*) of southern Britain emerged in the century before the Roman conquest of ad 43.

## Iron Age migration into southern England

An increase in continental ancestry components has been described for Iron Age genomes from the south of Britain (England and Wales)^17^ and has been interpreted as the result of large-scale movements into the island during and before the Late Bronze Age (around 1000 to 875 bc). This is detectable as a rise in Early European Farmer (EEF) ancestry (Supplementary Note 6.2). When we incorporate our data, we find a previously undetectable significant (Welch’s *t*-test, two-tailed, *P* = 0.0005) increase in EEF ancestry between the Early and Late Iron Age (from 39.7% ± 0.2% to 41.8% ± 0.5%), driven by genomes from southern regions along the central and eastern English Channel coast, including those from the Durotrigian territory (Fig. 3d and Supplementary Table 25). These regions emerged archaeologically as a core of unprecedented continental influence during the Middle Bronze Age, with cross-channel communities exhibiting parallel developments in disposal of the dead, settlement architecture and material culture over centuries, suggestive of high levels of population mobility^3^. Close cross-channel relations persisted throughout the Iron Age, when much of Britain seems to have developed a more regional and distinctively insular cultural footprint.

**Extended Data Fig. 7:**
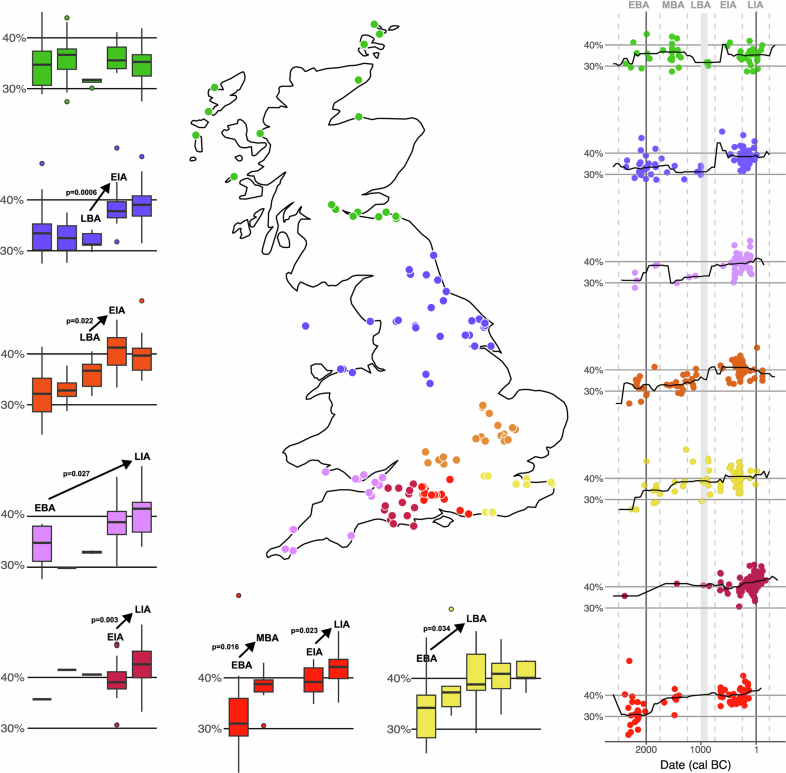
Early European Farmer (EEF) Ancestry through time in British regions. The percentage of EEF ancestry is shown on the y axis, estimated with qpAdm following the approach of Patterson et al. (2022)^17^. The map shows the location of Bronze and Iron Age sites used for analysis. No genomes post-dating AD 250 were included. Samples are grouped by geography, with the “channel core” zone south of the River Thames subdivided into western (dark red), central (red) and eastern (yellow) regions. On the right-hand side, EEF ancestry is plotted against the date estimate for each genome and a rolling average line is shown (window size: 500 years, step size: 50 years). On the left-hand side, boxplots (Tukey’s method) show the spread of values for different 500-year time bins for each region. These bins are demarcated with grey dashed lines on the rolling average plot. Time bin date ranges are labelled with respect to the approximate archaeological period they centre on (EBA: Earlier Bronze Age, MBA: Middle Bronze Age, LBA: Later Bronze Age, EIA: Earlier Iron Age, LIA: Later Iron Age). Significant changes (Welch’s *t*-test, two-tailed; p < 0.05) between bins (n > 4) are highlighted with arrows. The period between 1000-875 BC is highlighted with a grey rectangle in the rolling average plots. This period has been previously associated with a population-wide increase in EEF ancestry in southern Britain^17^.

When we split the genomic dataset into ‘channel core’ and ‘peripheral’ regions, we find that the rise of EEF ancestry during the Bronze Age was not a unitary process. Rather, the major increase in the channel core zone occurs across the Early to Middle Bronze Age, whereas a centuries-long lag is observed in the peripheral regions. For example, further regional division shows no increase in EEF ancestry in northern England from the Early Bronze Age until the Early Iron Age (around 750–400 bc; Extended Data Fig. 7).

The impact of continental gene flow specific to the channel core zone is visible in principal-components analysis (PCA) of modern and ancient western Europeans (Extended Data Fig. 2), as well as patterns of haplotype copying from continental populations, characterized using ChromoPainter^36^ (Fig. 3b). We used SOURCEFIND^37^ to decompose the ancestry of Iron Age genomes into contributions from Early Bronze Age British and continental groups and further validated our results using an alternative approach of non-negative least squares^38^ (NNLS) with a different panel of surrogates (Methods and Supplementary Note 6.3). Overall, we estimate an average contribution of 73% (estimated by SOURCEFIND; NNLS estimate: 75%) from the British Early Bronze Age (2500 to 1500 cal bc) to the English and Welsh Iron Age population (800 bc to ad 50). Although this value is larger than the estimate of a previous study^17^, which inferred a 50% long-term replacement rate for the gene pool, it is in agreement with the reported dilution of British- and Irish-specific R1b-L21 haplogroup Y chromosomes by one quarter^17^.

**Extended Data Fig. 8:**
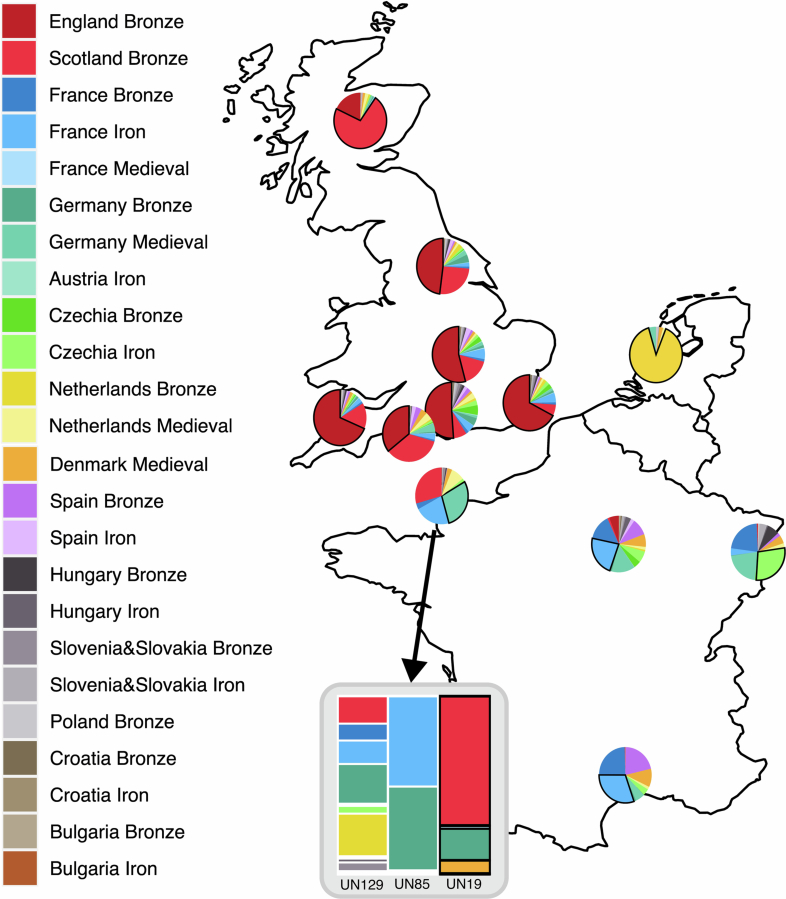
SOURCEFIND Ancestry Profiles for western Iron Age populations. Surrogate and target individuals are listed with their population IDs in Supplementary Table 12. Individual ancestry profiles for the French site of Urville-Nacqueville are also shown^41^, two of which (white outline) are below the coverage threshold to which the remainder of the dataset was subject. They are included here to demonstrate that the individual (UN19) with high levels of British Early Bronze Age ancestry is likely an outlier at the site, in agreement with the projection PCA analysis (Extended Data Fig. 2). Note that the French Iron Age samples used as a surrogate population are 1240k SNP capture data, while French target populations on the map are whole genome shotgun sequence data.

A sharp dip in Bronze Age continuity is seen along the channel coast (Fig. 3b and Extended Data Fig. 8). This is centred on Hampshire (SOURCEFIND estimate of 60%), a region traditionally associated with Belgic tribes that Caesar mentioned as having migrated from Gaul^3^. Both Hampshire and the neighbouring Durotrigian zone show independent and significant increases in EEF ancestry between the Early and Late Iron Age (Extended Data Fig. 7). Notably, the Durotrigian territory was home to a major port at Hengistbury Head, one of the focal points of intensifying cross-channel networks as Roman influence spread across Gaul^39^. With fewer samples for analysis, haplotypic data provide less resolution on fine-grained temporal trends but identify numerous genetic outliers in the Middle to Late Iron Age, all from the channel core region, which are not discernible when EEF ancestry alone is considered (Extended Data Fig. 3; see Supplementary Note 6.3 for further discussion of genetic outliers). These outliers include one of the most elaborate warrior burials known for Iron Age England (North Bersted on the channel coast; around 50 cal bc), which has been proposed, on the basis of isotopic signature and burial rite, to belong to a stream of cross-channel migrants, fuelled by Caesar’s conquest of Gaul^40^.

## Insular continuity

Regional continuity is strongest in Scotland, estimated at 92%, with contributions preferentially coming from the Scottish Early Bronze Age population (Extended Data Fig. 8 and Supplementary Note 6.3). Large components of Early Bronze Age ancestry are also seen in northern England (88%) and the southwest (78%). Outside of Britain, a single Netherlands Late Iron Age genome also shows some evidence of population continuity, deriving its ancestry almost entirely from the Netherlands Bronze Age population in SOURCEFIND analysis. By contrast, French populations show a diversity of components, mainly from French and German sources, but with large minor components of Czech Iron Age ancestry in the east and Spanish Bronze Age ancestry in the south, highlighting France’s position as a crossroads in the Celtic-speaking world. We note one French outlier from the coastal site Urville-Nacqueville^41^, which faces Dorset across the English Channel and contains Durotrigian-style flexed burials in shallow oval graves. This individual has an estimated 72% contribution from the British Bronze Age, implying that gene flow occurred in both directions across the channel.

## Conclusions

The diverse geography of Britain lends itself to regionality, which manifests across archaeological periods^3^. In its Iron Age we characterize fine-grained geographical genetic structure, shaped by natural territorial boundaries such as rivers. The peripheral regions—including Scotland, Cornwall, Wales and northern England—show signatures of insularity. The southern channel core is an exception, showing reduced genomic continuity with the British Early Bronze Age, sites with cross-channel IBD affinities, indications of larger population size and individuals with outlying ancestries. In this region, we see a Middle to Late Iron Age spike in EEF ancestry, indicative of substantial cross-channel movements that match textual and archaeological evidence for an intensification of contact and exchange, driven, at least latterly, by Roman expansion into Gaul.

The flow of genes across the channel through the Bronze and Iron Ages provides a wide window for the arrival of Celtic languages. Substantial components of continental ancestry are present in the channel core region by the Middle Bronze Age. However, it is probable that a second surge of EEF ancestry in the Iron Age would have influenced any version of insular Celtic already spoken in the channel region, and we note that the Celtic languages of southern Britain (Brittonic) and Gaul share a number of innovations not seen in more peripheral branches, such as the Goidelic languages of Ireland and Scotland^42^. Given the strong signatures of Early Bronze Age continuity in most British regions, any language introduction after this period would have probably been driven by a demographic minority, potentially an elite.

It is possible that the pervasive matrilocal traditions of Iron Age Britain were also introduced from the continent, but, notably, reduced mtDNA diversity is pronounced in our peripheral populations (Fig. 2 and Extended Data Fig. 1). Matrilineal succession has previously been proposed for continental Celtic societies, on the basis of the discovery of a likely avuncular relationship between two ‘princely’ burials of the Hallstatt elites in Central Europe^43^. Matrilineal institutions may also have been present in the British Iron Age, given that social units based on unilineal descent are common in large agricultural societies that practise unilocal residence^4^. However, the burial of male spouses at WBK suggests that, if matrilineal descent groups existed in this society, they were limited in their function^26^. We note that in matrilocal societies with a weak avunculate, mother–daughter–sister relationships are generally given more emphasis, with women tending to enjoy relatively higher status and control over property^27^.

Both matrilocality and matriliny are predicted by cultural factors that increase female involvement in subsistence labour and decrease paternity certainty^28,29,44–46^. External warfare can encourage both of these through male absence and has long been theorized to induce transitions to matrilocality through various mechanisms^45,47,48^, a hypothesis recently strengthened through quantitative modelling^49^. Matrilocality also predicts a history of migration into a new territory, which often is accompanied by frontier warfare^4,45^. The British Iron Age was debatably a time of high societal violence, indicated by the early proliferation of hillforts, weapons, human remains displaying violence-related injuries and instances of intergroup conflict recorded by Roman writers such as Julius Caesar and Tacitus^50–53^. Importantly, although matrilocality does not necessitate female political and social empowerment, it is strongly associated with these^4,27,54–56^ and resonates with Roman descriptions of Celtic women^1^. Although classical depictions of conquered peoples are often viewed with scepticism, we find here some truths in these writers’ appraisal of Iron Age Britain.

## Methods

### Data generation

We sampled 57 burials for DNA sequencing from three sites in Dorset^2,20,57–59^—WBK (*n* = 55), Langton Herring (*n* = 1) and Maiden Newton (*n* = 1). Petrous bones were preferentially sampled (*n* = 46), alongside tooth roots (*n* = 10) and a single phalanx. Sample processing took place in clean-room facilities dedicated to ancient DNA research at Trinity College Dublin. DNA extraction was carried out following various protocols^60–63^ detailed in Supplementary Table 4. DNA extracts were treated with USER enzyme to reduce post-mortem deamination lesions, and double-stranded libraries were created for Illumina sequencing^61,64^. Library aliquots were amplified using Accuprime Pfx Supermix (Life Technologies) with sample-specific index primers (Supplementary Table 5). Paired-end or single-end sequencing was carried out on MiSeq, HiSeq 2500 and NovaSeq 6000 platforms (Supplementary Table 5).

### Sequence data processing

Exact P7 index matches were required for demultiplexing, with up to two mismatches allowed in the P5 index for paired-end data. Adapters were removed from single-end data with cutadapt^65^ and from paired-end data with AdapterRemoval^66^. Paired-end reads with an overlap of 11 bp were collapsed. Singleton reads and collapsed reads that required quality trimming were discarded. Reads were mapped to GRCh37 with decoy contigs (hs37d5) using BWA software^67^ with non-default parameters -l 16500, -n 0.02 and -o 2. Reads were sorted with SAMtools^68^, polymerase chain reaction duplicates were removed with Picard Tools v.2.0.1 and indels were locally realigned using GATK software (v.3.7.0)^69^. Reads with a mapping quality below 25 and a read length below 34 bp were removed. Finally, we ‘soft-clipped’ the data by reducing the Phred quality scores of the two terminal base pairs at the 5′ and 3′ read ends to a score of 2. Comparative ancient genomic sequence data were downloaded and realigned from either unaligned FASTQ (when available) or BAM (aligned binary alignment map) files following the same pipeline (Supplementary Table 12).

### Uniparental markers

A detailed description of uniparental marker analysis is found in Supplementary Note 2. In brief, for mitochondrial haplotype calling, unfiltered read data aligned to GRCh37 were realigned to the Cambridge Reference Sequence for human mtDNA and subjected to the same downstream filters as described for GRCh37 alignments. Variants were called using BCFtools (v1.10.2)^70^, and the resulting VCF (variant call format) file was inputted into HaploGrep2 (ref. ^71^) to assign haplogroups based on Phylotree (Build 17)^72^. To estimate contamination, we calculated the fraction of minor alleles at HaploGrep-identified single-nucleotide variant sites present in the sample (Supplementary Table 7). Haplotype diversity (*h*) for archaeological sites was calculated as the probability that two randomly selected haplotypes were different^73,74^ (Supplementary Table 13). For Y chromosome haplotype calling, we relaxed several filters in our read processing pipeline: (1) we did not require an exact P7 index match; (2) we included singletons and collapsed reads that required quality trimming; (3) we filtered for a mapping quality above 20 and read length above 30 bp; and (4) we did not carry out soft-clipping. We used the Pileup tool from GATK (v.3.7.0)^69^ to extract base calls for positions in the International Society of Genetic Genealogy (ISOGG) database of Y chromosomal markers (version 15.73, 11 July 2020) and The Big Tree database (https://www.ytree.net/). Base calls below a quality of 30 were removed. The allelic state for each male sample at relevant markers was then assessed (Supplementary Table 9). Haplogroups used for within-site estimates of Y chromosome diversity in Britain are presented in Supplementary Table 18.

### Pseudo-haploid analysis

We used pseudo-haploid genotypes for PCA and quantification of EEF ancestry. We used the Pileup tool from GATK software (v.3.7.0)^69^ to extract base calls over single-nucleotide polymorphism (SNP) sites in the 1,240k panel^75^ for relevant genomes and selected one base call at random (base quality >30) for each site to generate pseudo-haploid genotypes. We merged 1,240k genotypes for 534 Iron Age individuals^17–19,41,61,76–80^ with a dataset of 5,326 modern individuals from western Europe^38,81^ and, using approximately 266,000 sites common to both datasets, projected ancient genomes onto a PCA plot of modern variation using smartpca (version 16000) from EIGENSOFT^82^. We quantified EEF ancestry in British Iron Age genomes following a previously described procedure^17^. In brief, the qpAdm tool^83^, implemented in the ADMIXTOOLS2 R package, was used to model British Bronze and Iron Age genomes as a mixture of western hunter-gatherer, EEF and steppe pastoralist ancestries (Supplementary Tables 12 and 15). Whole-genome sequence data, rather than targeted SNP capture, were used for source and reference outgroup populations. Source populations^61,76,79,84–87^ were a set of Mesolithic individuals from northwest Europe (*n* = 13), Yamnaya pastoralists (*n* = 6) and Early Neolithic Europeans from central and southeastern Europe (*n* = 9). Reference populations^79,84,88–90^ were a set of Mesolithic individuals from Latvia and Romania (*n* = 6), Afanasievo pastoralists (*n* = 4), Anatolian Neolithic farmers (*n* = 11) and 10 modern-day Mbuti individuals from the Congo region of Africa^91^. Further information on PCA and qpAdm analyses is provided in Supplementary Note 6.

### GLIMPSE imputation

We carried out genotype imputation on a dataset of 2,054 ancient individuals, including 42 individuals from the current study using GLIMPSE software^92^ (Supplementary Table 12). This included both whole-genome sequence (>0.1×) and targeted SNP capture (more than 300,000 calls across the 1,240k panel) datasets. After imputation, we further filtered for low-coverage individuals by extracting 1,240k panel positions and removing individuals for whom more than 40% of those positions had a genotype probability below 0.99. Stricter downstream filters were subsequently applied depending on the downstream analysis. To avoid any potential batch effects, we imputed each sample individually with GLIMPSE using the 1000 Genomes Project haplotype reference panel^93^. We used reference datasets and pipelines available on the software’s webpage (https://odelaneau.github.io/GLIMPSE/glimpse1/).

### IBD segment identification

Four datasets of GLIMPSE-imputed diploid genotypes (genotype probability >0.99) were subjected to IBD segment identification (Supplementary Table 12). To identify segments, each of the four datasets was subjected to further phasing and imputation using Beagle5 (ref. ^94^), followed by refinedIBD analysis (Supplementary Note 3). Different sets of variant sites were used as input into both Beagle5 and refinedIBD to test performance and maximize IBD segment retrieval. This resulted in 21 runs of refinedIBD in total, all carried out with default parameters. The outputted IBD segments were subsequently subjected to different merges and filters depending on the downstream application. Patterns of IBD segment sharing were characterized within (ROH) and between genomes, as well as within and between archaeological sites (Supplementary Note 5). We created a weighted graph of average IBD sharing between Iron Age sites in northwest Europe and performed hierarchical community detection using the Leiden algorithm^95^ implemented in the R package leidenAlg (v1.1.1)^96^. We ran the leiden.community function 100 times with different seeds and constructed a consensus tree from the output using the maximum clade credibility function available in the R package phangorn (v2.11.1)^97^.

### Pedigree construction

To reconstruct familial relationships at WBK, we used a combination of data types, including (1) uniparental markers; (2) autosomal coefficients of relatedness that were calculated using both allele-frequency-based methods and IBD segment sharing; (3) IBD1 and IBD2 segment numbers and lengths for genomes with more than 0.3× coverage, which were compared with distributions simulated using ped-sim^98^; (4) longest observed IBD segments within the genome; and (5) X chromosome IBD segment sharing. We determined the most likely genealogical relationships for pairs of relatives of first- to fourth-degree relatives (Supplementary Note 4), allowing us to construct the most parsimonious pedigree for the WBK kin group.

### Generating ancestry profiles with ChromoPainter

We used a dataset of 697 individuals^17–19,41,76–78,99–106^ from the European Bronze Age to medieval period for ChromoPainter^36^ analysis (Supplementary Table 12). This dataset had been previously subjected to Beagle5 imputation and phasing. We extracted 1,240k SNP sites and rephased these using SHAPEIT2 (v2.r837)^107^. Two separate panels of surrogate individuals were then selected and ChromoPainter was used to generate co-ancestry matrices summarizing the amount of haplotypic donations between pairs of surrogates following recommended guidelines. One panel (*n* = 332) was then subjected to fineSTRUCTURE clustering using a previously described maximum concordance tree-building method^38^. This panel was used to paint a set of British Iron Age genomes, whose ancestry was then decomposed into contributions from the identified fineSTRUCTURE clusters (*n* = 17) using NNLS regression. The second panel (*n* = 307) was grouped into populations based on archaeological era and geographical location, rather than fineSTRUCTURE cluster, and contained only targeted SNP capture data. This panel was used to paint a larger set of British Middle to Late Bronze and Iron Age genomes, as well as Iron Age genomes from France and the Netherlands. Target populations included both whole-genome sequence and SNP capture data. Ancestry profiles were then generated using SOURCEFIND^37^. SOURCEFIND was run using 50,000 burn-in iterations followed by 200,000 sample iterations, thinning every 5,000 iterations. We set the expected number of surrogates used to form the target as two, with a total number of four surrogates allowed to form the target in each iteration. We carried out 50 independent runs of the above procedure and extracted the estimates with the highest posterior probability in each run. The average of these 50 estimates (weighted by posterior probability) was then calculated for each individual. This provided us with a set of ancestry proportions for each genome. We observed a strong correlation between SOURCEFIND and NNLS results with respect to British Bronze Age haplotype contributions. Further details can be found in Supplementary Note 6.3.

### Data visualization

The R package ggplot2 was used for figure generation (https://ggplot2.tidyverse.org). Maps were generated using the R packages maps (10.32614/CRAN.package.maps) and mapdata (10.32614/CRAN.package.mapdata). For Extended Data Fig. 4, the retired rgeos package and raster package were used, with data from the public Database of Global Administrative Areas.

### Reporting summary

Further information on research design is available in the Nature Portfolio Reporting Summary linked to this article.

## Online content

Any methods, additional references, Nature Portfolio reporting summaries, source data, extended data, supplementary information, acknowledgements, peer review information; details of author contributions and competing interests; and statements of data and code availability are available at 10.1038/s41586-024-08409-6.

## Supplementary information

Supplementary Information Supplementary Notes 1–6, Figs. 1–35 and Tables 19–25.

Reporting Summary

Supplementary Tables Supplementary Tables 1–18.

## Data Availability

Aligned sequence reads are available through the European Nucleotide Archive under accession number PRJEB81465. Other relevant data are available from the corresponding authors on reasonable request.

## Code availability

This study made use of publicly available software, referenced throughout the main text and Supplementary Information.
