# Author Correction: Continental influx and pervasive matrilocality in Iron Age Britain

**DOI:** 10.1038/s41586-025-08679-8

**Published:** 2025-01-28

**Authors:** Lara M. Cassidy, Miles Russell, Martin Smith, Gabrielle Delbarre, Paul Cheetham, Harry Manley, Valeria Mattiangeli, Emily M. Breslin, Iseult Jackson, Maeve McCann, Harry Little, Ciarán G. O’Connor, Beth Heaslip, Daniel Lawson, Phillip Endicott, Daniel G. Bradley

**Affiliations:** 1 Department of Genetics, Trinity College Dublin, Dublin, Ireland; 2 Department of Archaeology and Anthropology, Bournemouth University, Bournemouth, UK; 3 Department of Life and Environmental Sciences, Bournemouth University, Bournemouth, UK; 4 School of Mathematics, University of Bristol, Bristol, UK; 5 Institute of Genomics, University of Tartu, Tartu, Estonia; 6 Department of Linguistics, University of Hawai‘i at Mānoa, Mānoa, HI USA; 7 DFG Center for Advanced Studies, University of Tübingen, Tübingen, Germany; 8 Éco-anthropologie, Musée de l’Homme, Paris, France

**Keywords:** Population genetics, Archaeology, Evolutionary genetics, Social anthropology

Correction to: *Nature* 10.1038/s41586-024-08409-6 Published online 15 January 2025

In the version of the article initially published, Phillip Endicott’s first affiliation was the “Department of Genomics...” and has now been amended to the “Institute of Genomics, University of Tartu, Tartu, Estonia” in the HTML and PDF versions of the article.

