# Supplementary material for: Continental influx and pervasive matrilocality in Iron Age Britain
Source: Nature. 2025 Jan 15;637(8048):1136–42. doi: 10.1038/s41586-024-08409-6 (PMC11779635; doi:10.1038/s41586-024-08409-6)
Supplement: Supplementary file 1 — Supplementary Notes 1–6, Figs. 1–35 and Tables 19–25. [file 41586_2024_8409_MOESM1_ESM.pdf]

nature portfolio

https://doi.org/10.1038/s41586-024-08409-6

Supplementary information

Continental influx and pervasive matrilocality in Iron Age Britain

In the format provided by the authors and unedited

Supplementary Notes

Continental influx and pervasive matrilocality in Iron Age Britain

S1. Archaeological and Linguistic Context **2**
1.1 Iron Age England 2
1.2 The Durotriges culture 2
1.3 The Durotriges Project: Winterborne Kingston, Dorset 3
1.4 Notes on Langton Herring 7
1.5 Notes on Maiden Newton 8
1.6 Insular Celtic 8
S2. Uniparental Marker Analysis **10**
2.1 Mitochondrial data processing and haplogroup classification 10
2.2 Estimating mitochondrial haplotype diversity within archaeological sites 11
2.3 Mitochondrial diversity of the Iron Age British population 12
2.4 The U5b1 haplogroup at Winterborne Kingston 12
2.5 Subclades of U5b1-a1c1 at Winterborne Kingston 13
2.6 Minimum number of germline transmissions in the Winterborne Kingston matriline 14
2.7 Other matrilineages present in the WBK kin-group 14
2.8 Y chromosome diversity at Winterborne Kingston 14
2.9 Y chromosome diversity in Iron Age Britain 15
2.10 Modelling the impact of sex-biased migration on uniparental diversity 19
S3. IBD Segment Retrieval for Imputed Ancient Genomes **23**
3.1 Dataset preparation 23
3.2 Imputation 25
3.3 IBD segment retrieval 25
3.4 Assessing IBD retrieval rates with parent-offspring pairs 26
3.5 IBD segment identification on the X chromosome 30
S4. Resolving Pedigree Relationships at Winterborne Kingston **32**
4.1 Coefficient of relatedness 32
4.2 A note on sample WBK195 34
4.3 Estimating the degree of relationship 34
4.4 IBD2 segment retrieval 40
4.5 X chromosome IBD sharing and simulations 40
4.6 Resolving 1st degree relationships 43
4.7 Resolving 2nd degree relationships 44
4.8 Resolving a 2nd degree double relationship 49
4.9 Resolving 3rd degree relationships 53
4.10 Resolving 3rd-4th degree relationships 63
4.11 Resolving more distant relationships 66
S5. RefinedIBD Analysis of Iron Age Britain **68**
5.1 Dataset preparation 68
5.2 Detecting relatives 68
5.3 Patterns of IBD sharing within archaeological sites 69
5.4 Population and community sizes in the British Iron Age 70
5.5 Incidences of inbreeding at Winterborne Kingston 71
5.6 Relatives within and between British Iron Age sites 74
5.7 IBD sharing between Iron Age communities in northwest Europe 80
S6. Population Structure and Admixture **83**
6.1 Projection PCA 83
6.2 qpADM 85
6.3 ChromoPainter, fineSTRUCTURE and SOURCEFIND 89
References **103**

S1. Archaeological and Linguistic Context

1.1 Iron Age England

The settlement pattern recorded for the English Iron Age (8th century BC – mid 1st century AD) is rich and varied, archaeological investigations revealing evidence for a diverse and complex society occupying a landscape of farming settlements, field-systems and monumental earthwork enclosures[1–6] all of which is consistent with a comparatively large and thriving population. The physical nature of this record, however, is contrasted by the relative paucity of human remains, burials being all but unknown for many regions of Britain[7,8]. The most accepted explanation for this is that the dead were perhaps disposed of in a variety of archaeologically undetectable ways, such as excarnation, total cremation or deposition in rivers, lakes or bogs. Two parts of Britain that stand out as the exception to the rule of a near total absence of an Iron Age burial rite, are both chronologically and geographically restricted[8], comprising eastern Yorkshire and central southern England (Dorset, Hampshire, Kent and Sussex)[7,9]. The reasons behind such distinctive disposal practices for the dead in these particular areas remain unknown, although it is to them that biological anthropology must turn if it is to contribute to an understanding of Iron Age life and society.

1.2 The Durotriges culture

The area today largely covered by the county of Dorset was, in the Late Iron Age and early Roman period (c. 100 BC – AD 100) occupied by a social group identified, by the second century AD writer Ptolemy, as the Durotriges (*Geographia* II, 2). Although we cannot be sure how real this identification was, and whether or not it reflected the reality of Iron Age life in this part of Britain, it is clear that, archaeologically speaking, the Durotriges were distinct from their immediate neighbours in terms of their coinage, pottery, settlement-forms and burial practices[10–13].

With regard to body disposal, the Durotriges practised inhumation, albeit with considerable variation in orientation and associations. The 'typical' Durotrigian burial is flexed, legs drawn to the chest in a foetal position, usually lying on the right side, head at the east facing north[6–8,14,15]. Grave goods are rare but, where found, usually comprise locally manufactured bead-rim bowls and handled tankards[8,14], as well as imported Samian and Gallo-Belgic wares of the early and mid-first century AD[7,12,16], female inhumations being associated with the greater diversity and number of prestige items[17]. Joints of meat, where located, may have been subject to a degree of gender selection, sheep / goat being more closely associated with male burials and pig more common with female[8,16]. Dress accessories often include simple copper alloy brooches, placed at the head or on the chest, bangles and rings[15,16,18–20]. In exceptional cases, more unusual forms of metalwork, such as decorated bronze mirrors and toilet sets, have been noted[16,17,19,21].

Durotrigian inhumations, in oval-shaped grave-cuts and stone-lined cists[8,14] have been found either as isolated single features or clustered together in small cemeteries[16,19,22,23]. Sometimes burials seem to have deliberately targeted earlier earthwork features, cemeteries being placed within the partially backfilled remains of abandoned monuments such as banjo enclosures[15,20] and hillforts[18,24,25].

1.3 The Durotriges Project: Winterborne Kingston, Dorset

In 2009, Bournemouth University established the Durotriges Project to examine the nature of Late Iron Age settlement and burial practice in central southern Britain and to assess the possible survival of prehistoric culture patterns into the Romano-British period. The focus of investigation to date has been a series of Iron Age, Roman and post Roman farmsteads, enclosures and cemeteries near Winterborne Kingston, to the east of Dorchester in Dorset[15,20,26,27] (Supplementary Figure 1). A series of human burial deposits have been found throughout the project, facilitating a diachronic analysis of burial treatment as well as providing observations on the changing nature and context of body disposal in the Late Iron Age[13].

The first phase of the project (2009-2013) involved the excavation of a Middle Iron Age banjo enclosure with associated settlement and Later Iron Age (Durotrigian) cemetery (Supplementary Figure 1). A second banjo enclosure was excavated between 2022-2023 with further burial activity discovered. The 2012-2013 season uncovered a small Later Roman cottage-style villa and a sub-Roman longhouse associated with multiple agricultural features (corn driers/malting ovens and storage pits). A small Late Roman or sub-Roman cemetery was found adjacent to the villa. In 2015-2016 an extensive roundhouse settlement area of Mid-Late Iron Age date was excavated, as well as Later Iron Age oval graves inserted into a Late Bronze Age ditch system. Relative pairs were found between the two Banjo enclosures and the Late Bronze Age ditch system. In 2017, a Durotrigian farmstead was discovered, occupied from the first century BC to the mid first century AD. There is evidence that this farmstead was abandoned relatively quickly in order to allow for more intensive agriculture in the Early Roman period. A single extended inhumation burial from the Roman period was found in this area (WBK36). This individual is an approximately 6th degree relative of an earlier individual buried in a Durotrigian fashion (WBK31; 104 cM shared).

Human burials

Between the first season of excavation (2009) and the time of the current analysis (2023), 50* articulated human burials had been excavated (Supplementary Table 2, Supplementary Figure 2), including 42 who had reached adulthood (here defined as having the majority of epiphyses fused and the third molar erupted). Of the eight sub-adults, five could be placed within the 'Child' category (3-12 years)[28], while

two were confirmed to be adolescents (14-16 years). Seven adult individuals could be described as buried in a 'Romanised' style, in extended supine positions and dated to approximately the 2nd to the 5th century cal AD. The remaining adult and adolescent burials, mostly dating between the 2nd century cal BC and the 2nd century cal AD, divide into three groups according to burial feature and location (Supplementary Table 19). Fourteen individuals were deposited in pits, interpreted as repurposed storage pits within the two banjo enclosures, sixteen individuals were buried in graves within or adjacent to the banjo enclosures (mainly banjo enclosure 1), whilst seven were placed in graves cut into an earlier Bronze Age ditch system. The adolescents were buried in the same style as the adult burials and were likely afforded the status of adults within their society at the time of their deaths.

The later Iron Age individuals buried in graves were deposited in flexed positions, mostly on their right sides. Grave inclusions consisted of pots, joints of meat and single brooches, on which basis these burials could be described as conforming to the Durotrigian style. The individuals placed in pits exhibited greater variability in positioning, whilst fewer had grave goods placed with them. Skeletal preservation was generally very good, both in terms of the survival of cortical surfaces[29] and with most burials suffering relatively limited fragmentation. As would be expected, the individuals buried at the greatest depths had suffered the least fragmentation and deterioration of cortical surfaces in the burial environment.

All samples with the exception of those excavated in 2023 were submitted for radiocarbon dating (Supplementary Table 3) yielding a range of dates spanning the Early Iron Age (775-515 cal BC) to post-Roman period (cal AD 268-544).

*This number excludes burials aged as infants /perinates. Further adult articulated inhumations were excavated during 2023 and 2024 which are also excluded from these figures.

	**Burial context**		
**Location**	Pit	Oval Grave	Extended grave
Banjo Enclosure 1 and 2	14	16	
LBA Ditch system		7	2
Square mortuary enclosure			5
**Total**	**14**	**23**	**7**

Supplementary Table 19. Adult burials by type and location.

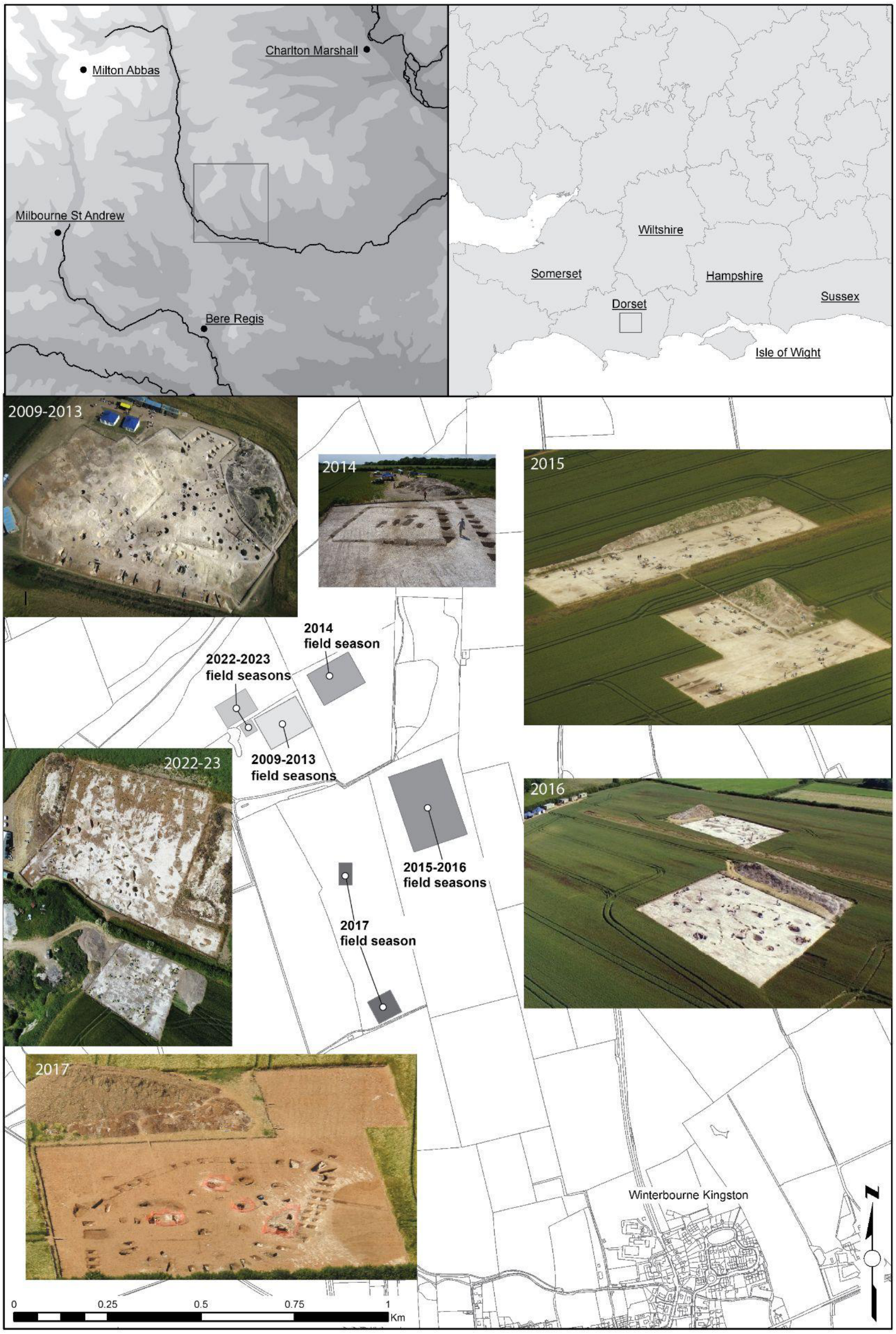

Supplementary Figure 1. Site map of Winterborne Kingston. Excavations have been ongoing since 2009. The 2009-2013 season (DBD09-DBD13) excavated Banjo Enclosure 1. The 2014 season (DBD14) excavated a set of Roman and sub-Roman features. The 2015-2017 seasons (DBD15-17) excavated a Late Bronze Age Ditch system. The 2022-2023 seasons (DBD22-23) excavated Banjo Enclosure 2. © Crown copyright and database rights 2024 Ordnance Survey (AC0000851941).

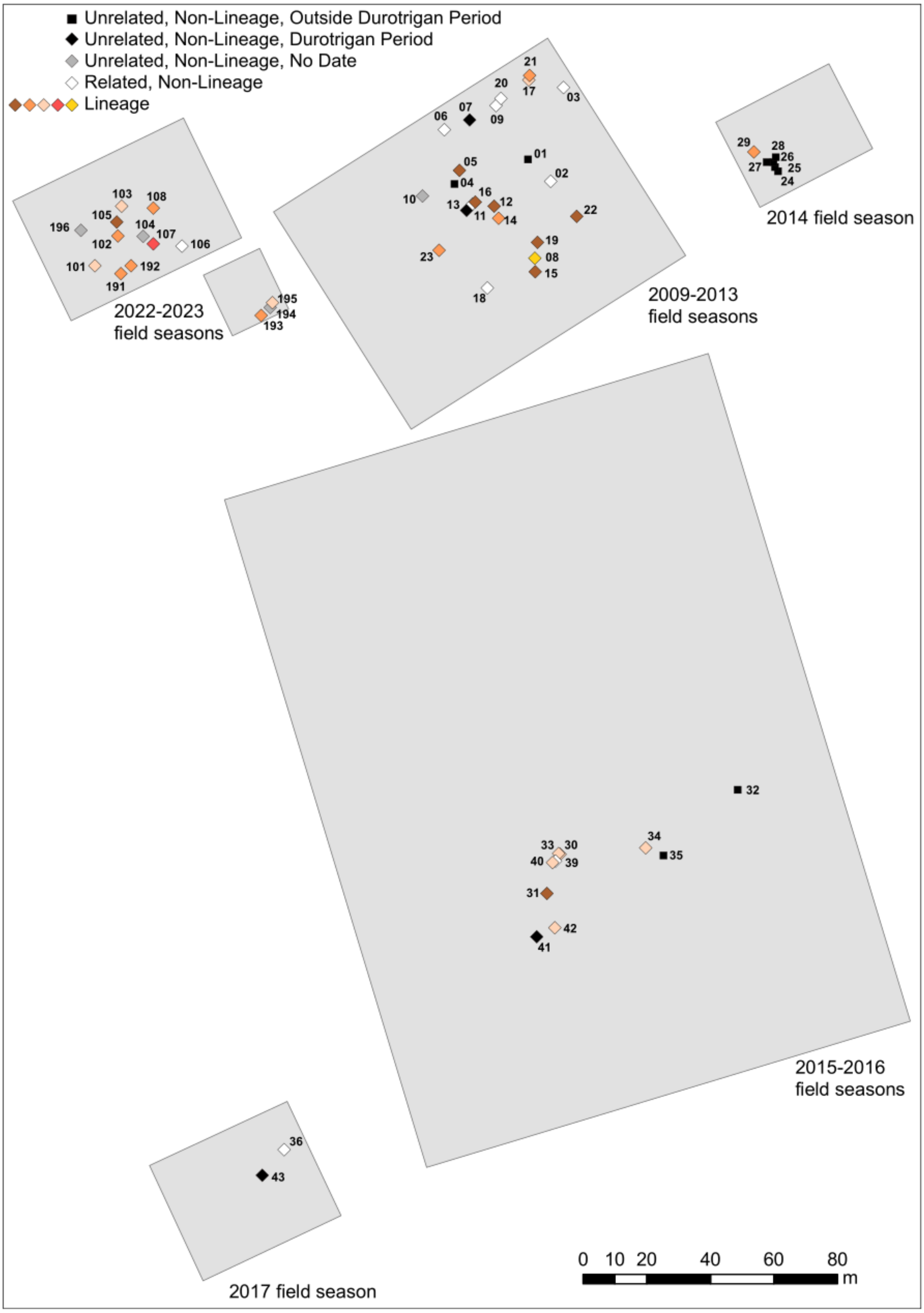

Supplementary Figure 2. Burial map of Winterborne Kingston. Graves are coloured based on time period, relatedness and mtDNA lineage. Only mtDNA haplotypes belonging to the dominant lineage are shown in colour, following the scheme provided in Figure 1. The WBK number is provided beside each grave.

1.4 Notes on Langton Herring

In April 2010 a metal-detectorist searching farmland at Langton Herring in south Dorset, England, came across a well-furnished, Late Iron Age Durotrigian burial, detected due to the presence of several metal artefacts. On noticing human remains to be present, the metal-detectorist notified the police, who requested assistance from Bournemouth University. A team from the university attended and were able to confirm the find as archaeological. The excavation that followed cleared a 1.5 m square area of ploughsoil to the geological natural, an olive-grey mudstone, exposing an oval-shaped grave, measuring 1.3m × 0.9 m. The grave contained an articulated human skeleton, lying in a flexed position on its left side, with the skull at the eastern end of the grave, facing south-west, in the direction of nearby Chesil beach. The artefact assemblage accompanying the body consisted of a silvered Roman coin, eight beads (three of stone, five made of glass), two bronze brooches, a spiral arm bracelet, a pair of tweezers and a bronze mirror with a decorated back plate.

The skeletal material recovered comprised the entire skeleton with the exception of a small number of hand and foot bones. Cortical surfaces were generally well preserved with no cracking or flaking and minimal root etching. The burial appeared to have lain undisturbed until the recent plough damage, although the majority of bones had fractured in the burial environment and so the material as excavated was highly fragmented with all of the larger and many of the smaller elements recovered as multiple conjoining fragments. There were no signs of vertebrate scavenging or subaerial weathering and the overall condition of the remains combined with the normal anatomical relationships of bones in the grave were consistent with a straightforward burial relatively soon after death.

The individual was assessed as a female, of gracile build, aged between 19-24, with an attained stature ranging between 156.82 and 163.92 cm. The individual lacked well developed muscle attachments (entheses). Signs of pathology were present in the form of deposits of woven bone/ subperiosteal new bone on 11 ribs, both distal tibiae and fibulae and several foot bones (the left calcaneus and metatarsal shafts of both feet). New bone deposits were also present in both maxillary sinuses. Whilst the latter are consistent with chronic sinusitis, the former widespread and symmetrical pattern would be more consistent with a systemic condition and may derive from Vitamin C deficiency. Stable isotopes of carbon and nitrogen obtained from a sample of bone indicated a terrestrially based diet, characterised by a high degree of animal protein. This latter point might further suggest this individual to have held relatively high status, as indicated by the well-furnished nature of the burial assemblage. Strontium and oxygen isotope values obtained from sampling tooth enamel indicated the individual to have spent her childhood in an area of chalkland. This result would be consistent with having originated in Dorset, although an origin elsewhere in a region with similar geology cannot be ruled out.

A combination of artefacts and radiocarbon dating (104 cal BC - cal AD 69) gives a range for the burial of *circa* AD 25 – 53. The grave goods themselves are of exceptional interest, representing an accumulation of artefacts acquired from diverse sources, deposited at a time of major cultural and societal change in southern Britain. The Copper alloy mirror is decorated in an insular, British version of the la Tène style. The handle had been replaced, indicating the item possibly to be old at the time it was buried. The coin was a silver-plated, copper-alloy Late Republican forgery of a denarius serratus, originally minted in Rome between 83 and 82 BC, and so was over a century old at the time of burial. The coin had been pierced and presumably worn as an amulet. The different coloured glass beads are possibly of continental origin. The full account of the Langton Herring burial is published in Russell et al. 2019[17].

1.5 Notes on Maiden Newton

In August 2016 a metal-detectorist searching farmland at Maiden Newton in central Dorset, England, came across a well-furnished, Late Iron Age Durotrigian burial, detected due to the presence of several metal artefacts. On noticing human remains to be present, the metal-detectorist notified the police, who requested assistance from Bournemouth University. A team from the university attended and confirmed the find as archaeological. The excavation that followed cleared a 1.5 m square area of ploughsoil to the geological natural, exposing an oval-shaped grave containing an articulated human skeleton, lying in a flexed position on its right side, with the skull at the eastern end of the grave, facing north-west. The accompanying artefact assemblage consisted of a copper alloy tankard with ornate handle, two copper alloy brooches (a large T-shape brooch of hinged-pin Colchester derivative type dated to the 1st or 2nd centuries AD and a long strip bow brooch dating to the mid-1st century AD), an iron bladed implement (scutching knife) similar to ones from a mid-1st century AD hoard at Hod Hill, Dorset, and a rounded pottery bowl of Late Iron Age black burnished ware.

The skeletal material, dated to 162 cal BC - cal AD 23, comprised the entire skeleton of an adult female, with the exception of a small number of hand and foot bones. Cortical surfaces were generally well preserved with no cracking or flaking and minimal root etching. The burial appeared to have lain undisturbed until the recent metal detectorist exposure. There were no signs of vertebrate scavenging or subaerial weathering and the overall condition of the remains combined with the normal anatomical relationships of bones in the grave were consistent with a straightforward burial relatively soon after death (further osteological analysis pending at time of writing).

1.6 Insular Celtic

The Insular Celtic languages are conventionally split into Goidelic (Irish, Manx, and Scottish Gaelic) and Brythonic (Welsh, Cornish, and Breton). The relationships between the insular and continental

branches of Celtic are unresolved. There are two competing subgroupings, in which the difference is the relationship of Gallic languages to Brythonic. The less accepted one has Transalpine Celtic and Brythonic/Brittonic as deriving from a common ancestor that is sister to Goidelic. The evidence is not robust for either configuration but most linguists side with the opposing phylogeny that has Transalpine Celtic as branching from the common ancestor prior to a division between Goidelic and Brythonic. The basis for this position is the dual flexional paradigm of verbs, and the grammaticalization of the PIE verbal adjective in *-tolā. In this version the similarities between Transalpine Celtic and Brythonic, such as the change from Proto-Celtic $*k^w$ into /p/, are considered to be either areal development of natural and repeatable phonological features, and/or derive from multiple waves of contact in both directions between Gaul and Britain[30,31].

On the question of the Celtic homeland and timing of expansion, linguists rely on written records and areal evidence from landscape. It is clear that the Brythonic dialects diverged in the fifth century CE and the Gaelic ones in the tenth century CE[32]. At the same time ancient Continental Celtic languages e.g. Gaulish, Galatian, Celiberian etc. were all dead by 500 CE. The lower bound for Celtic arriving to Ireland is the 1st Century CE dated by Celtic names attested in Ptolemy's Geography. From 400 BCE references to "Celts" in the Classical literature occur in connection to violent expansion into Italy, the Balkans, Greece and Turkey [32]. There are various references about the location of such peoples preceding this, including Herodotus, but it is unclear what they would mean by the term, and as to the accuracy of geographical locations, which ranged from Spain, southern France (hinterland of Marseilles) and the far west of Europe. Analysis of coins and inscriptions is undermined by the bias towards the Mediterranean and its early writing, but Celtic-looking placenames have a much higher density in Gaul and Britain. Importantly, we cannot be sure whether all peoples referred to as "Celts" by the classical authors were in fact Celtic-speaking, while the insular peoples of Britain and Ireland were never referred to by this term.

Various archaeological approaches have linked the expansion of Celtic languages to both the Hallstatt (Latest Bronze to Early Iron Age) and La Tène (Iron Age) cultures of western and central Europe. There is a good correlation between La Tène culture and Celtic speech in eastern Europe[33], but not in Spain or northern Italy. Earlier Urnfield groups, which preceded the Hallstatt culture, have also been linked to Celtic language expansion[34]. A longer association between Celtic and the Atlantic seaboard has also been suggested[35], although a dearth of maritime vocabulary in reconstructed proto-Celtic is suggestive of an inland speech community[36]. One recent model[37], places the emergence of Celtic somewhere in Gaul in the 2nd millenium BC, from whence it radiated "incrementally and unspectacularly" in the 1st millennium BC. It is noted that if Celtic expanded into regions which contained many minor languages, even a low number of Celtic speakers could induce a broader language shift if it possessed some social advantages.

S2. Uniparental Marker Analysis

2.1 Mitochondrial data processing and haplogroup classification

We assigned haplogroups for both the newly sequenced samples in this study, as well as a set of published ancient samples to allow for comparisons of haplotype diversity across different archaeological sites, time periods and geographic regions[34,38–56] (Supplementary Tables 12, 13). The majority of published studies provided raw read data or aligned read data with no mapping quality filter, allowing us to apply an identical processing pipeline to that implemented for the newly sequenced samples in this study (see below). Four studies provided reads aligned to the human reference genome with a mapping quality filter applied[45,46,54,56]. This results in the removal of mitochondrial reads in regions that show homology with nuclear DNA of mitochondrial origin (nuMTs), as they map to multiple locations. Thus, we could not securely assign haplogroups for these samples and instead used the haplogroup information provided by each publication. This may have the effect of artificially reducing haplotype diversity estimates, as samples placed in the same Phylotree haplogroup will be treated as identical, even though they may possess distinct haplotypes characterised by additional mutations.

Data was processed as follows. Endogenous reads aligned to some version of the human reference genome (study dependent), with no mapping quality filter applied, were realigned to the Cambridge Reference Sequence for the human mitochondrial DNA (rCRS). Alignment parameters and downstream filtering steps were the same as described for mapping to the human reference genome (see Methods). Variant calling was carried out using BCFTOOLS v1.10.2[57]. The mpileup tool (non-default parameters: -B, -Q30, -a FORMAT/AD,FORMAT/DP) was used in conjunction with the multiallelic calling model (bcftools call -m) with ploidy set to 1. The resulting VCF was provided as input into Haplogrep2[58] and haplogroups were assigned using the phylogenetic tree provided by Phylotree (Build 17)[59].

This VCF was also used to estimate mitochondrial genome coverage and contamination. To estimate contamination, we calculate the fraction of minor alleles at haplogrep-identified single nucleotide variant sites present in the sample. The average of this value across sites gives a conservative estimate of mitochondrial contamination, as minor alleles may also be the result of true heteroplasmy, sequencing error, alignment error and post-mortem damage.

Results for the newly sequenced data are shown in Supplementary Table 7. We found contamination estimates ranged from 0-8.14% with a median value of 1.04%. Haplogrep quality scores were above 0.88 for all assignments. Additional polymorphisms present in samples but not associated with their assigned Phylotree haplogroup are also shown in Supplementary Table 7. We exclude variants in the

homopolymer stretches at 302-315 bp and 3105-3109 bp as the length of the cytosine stretches here are highly polymorphic. We also exclude variants at position 16519, as the mutation rate at this site is disproportionately fast relative to the rest of the mitochondrial genome[60].

2.2 Estimating mitochondrial haplotype diversity within archaeological sites

Low levels of mitochondrial diversity in a community may reflect matrilocal practices. To compare the mitochondrial diversity seen at Winterborne Kingston to other communities, we grouped samples by archaeological site and period (Neolithic, Copper-Bronze Age and Iron Age). We removed ancient individuals with a mitochondrial genome coverage below 3x or a Haplogrep2 quality score below 0.85. We also removed one member from each pair of 1st degree relatives in the dataset, as multiple sibling or mother-offspring pairs can decrease mitochondrial diversity without being informative on broader social organisation.

Haplotypes were defined using both the haplogroup provided by Haplogrep2 and the list of additional mutations identified in the sample but not present in Phylotree (Build 17). Indel variation was not considered. We also excluded highly mutable sites by removing mutations observed across three or more different haplogroups. Haplotype diversity (*h*), defined as the probability that two randomly selected haplotypes are different[61,62], was then calculated as follows:

$$h = \frac{n}{n-1}\left(1 - \sum_i p_i^2\right)$$

Here, *p* is the frequency of each of the *i* distinct haplotypes in the sample and n is the sample size.

We note that a lack of mitochondrial diversity within a given archaeological site during a particular period may not necessarily reflect no practices (e.g. patrilocality). Such an observation could be due to an overall lack of biological relatedness among the individuals sampled. For example, some sites may have been used by different communities over long time spans or burial may not have been guided by kinship (e.g. mass graves resulting from violence or disease). To account for this, we plotted haplotype diversity against the normalised number of relative pairs (*rel_norm*) identified using refinedIBD (Figure 2; Supplementary Note 5.3). 1st degree relatives were not filtered in this case, as they are informative on a site's usage. The results are displayed in Supplementary Table 13.

2.3 Mitochondrial diversity of the Iron Age British population

We also estimated haplotype diversity within the Iron Age British population as a whole, using the same approach as described above. We found mitochondrial diversity to be extremely high (*h*-value=0.994). We identified 227 distinct haplotypes in the population of 322 individuals. These are defined using the

Phylotree haplogroup assigned by Haplogrep2 alongside additional mutations outside of highly mutable sites (see above). We found 28 haplotypes that occurred more than once within archaeological sites and 18 that occurred in multiple archaeological sites. Sixteen haplotypes occurred in two archaeological sites, while only two haplotypes (H2a1 and H7b with no additional mutations) were observed in three or more sites.

2.4 The U5b1 haplogroup at Winterborne Kingston

Approximately 50% of the samples from Winterborne Kingston were placed in the same mitochondrial haplogroup, assigned as U5b1+16189+@16192 by Haplogrep2. Haplogroup U5b is common in Britain today. It is at a frequency of 4% in the UK Biobank, the seventh most frequent major clade observed in the dataset[63]. However, the specific haplotype observed at Winterborne Kingston is very rare today, albeit geographically widespread in Northern Europe.

The Winterborne Kingston U5b1+16189+@16192 haplotype is defined by four additional mutations: 93G, 15607G, 15928T and 16362C. To the authors' knowledge, this haplotype has not been reported for any ancient individual in the literature (approximately 6,200 samples considered). We also queried publically available modern databases from GenBank and YFull. We first considered 61,168 human mtDNA sequences from GenBank, made available through MitoMap[64]. We found 614 individuals belonged to U5b1, of whom 33 were assigned as U5b1+16189+@16192. Of these 33, five individuals possessed the 15928T and 16362C mutations, two of whom also possessed the downstream 15607G and 93G mutations. These two samples were taken from individuals in France (KF451059.1) and Poland (MH120619.1).

The public genealogical database from YFull (www.yfull.com; v11.04.00) lists five individuals who carry the 15607G mutation and defines this haplogroup as U5b1-a1c1, dating its formation to 6300 BP. These five include the aforementioned KF451059.1, an individual from Finland and three of unknown location. Given the rarity of U5b1-a1c1 in modern and ancient Europeans, we conclude that its high frequency in the Winterborne Kingston population is due to a founder event, with very recent shared female-line ancestry among these individuals. We note that inbreeding coefficients are low in the population (Supplementary Note 5.5), ruling out a scenario whereby this haplogroup rose to high frequency due to demographic bottleneck. Rather, its dominance at the site must reflect the kinship system of this community and matrilocal practices.

2.5 Subclades of U5b1-a1c1 at Winterborne Kingston

We further find four distinct subclades of U5b1-a1c1 present at Winterborne Kingston, each defined by a specific mutation: 16182C (n=7), 14544A (n=9), 151T (n=1) and 13011T (n=1). We note that two of

these positions are in the faster evolving hypervariable regions of the mtDNA (151T and 16182C). We failed to identify any U5b1+16189+@16192 individuals in GenBank or YFull who possess the 14544A, 13011T or 151T mutations. This suggests that these are private mutations that arose within the Winterborne Kingston population and implies a long-term association between this haplogroup and the site.

The 16182C mutation forms the start of a homopolymeric cytosine sequence and thus requires additional validation, as this region is more prone to alignment error (Supplementary Table 8). For each sample, we extracted reads (length > 34bp) that covered the region 16175-16190 bp and found the proportion of reads that carried the motifs: CATC**AAAA**CCCCC; CATC**AAA**CCCCC and CATC**AA**CCCCC. In the majority of U5b1-a1c1 samples, the dominant motif (>75% of reads) was CATC**AAA**CCCCC, indicating a A->C mutation at position 16183. An A-deletion is also possible, given the homopolymeric cytosine sequence that follows which is highly variable in length.

In eight U5b1-a1c1 samples the CATC**AA**CCCCC motif was dominant. This group consisted of two sisters (WBK15 and WBK19), their mother (WBK22) and maternal grandmother (WBK31), a 3rd degree relative of the grandmother (WBK12), and a more distant relative of the grandmother, WBK16, as well as the son of WBK16 (WBK05). It also included WBK195, who is a ≥5th degree relative of WBK12 and WBK16. This implies an additional A->C mutation at position 16182 or additional A-deletion in a shared maternal ancestor of these samples. We note that WBK195 only had one read covering the relevant mutation. When we relaxed our read length filter to 30bp, we observed another read carrying the CATC**AA**CCCCC motif. However, given the low coverage and highly mutable nature of the site, this result should be taken with caution.

Finally, we note that for one sample (WBK30) only two reads covered the region but both carried the motif CATC**AAAA**CCCCC. However, it is likely that these reads are misaligned or contaminants, as neither carries the downstream 16189C mutation that defines the U5b1+16189+@16192 haplogroup. When two additional reads that do not cover the entire 16175-16190bp region are considered, we observe a CATC**AAA**CC motif, indicating that this sample is also positive for 16183C, but not 16182C.

Only one sample was of a coverage low enough for read data to be missing over relevant haplogroup-defining sites (WBK102; 2.11x). However, it could still be securely assigned to the dominant matrilineage at Winterborne Kingston and further placed into the 14544A subclade, with three reads covering the defining mutation.

2.6 Minimum number of germline transmissions in the Winterborne Kingston matriline

Two widely used estimates of the germline mtDNA mutation rate are 2.7×10^{-7} and 4.72×10^{-7} mutations per site per generation[65,66]. Respectively, these predict either 0.0045 or 0.0078 new mutations per genome per generation. This translates into approximately one de novo mutation every 130-220 births. Thus, we estimate that between 420-880 female births to matrilineage mothers would be required to produce the observed diversity of haplogroup U5b1-a1c1 at WBK, given the presence of four likely de novo mutations. If we assume the founder lived *circa* 225 BC, then we would expect 510 mother-daughter transmissions by 10 BC (median probability for kin-group samples) in the population under the following conditions:

1. Each U5b1-a1c1 woman had two daughters survive to adulthood on average (fertility rate of 6 children per woman, with two thirds reaching adulthood).
2. No outward female migration.
3. Generation time 27 years.

2.7 Other matrilineages present in the WBK kin-group

We carried out an additional *h*-value calculation for the WBK population, where we considered individuals who did not belong to the U5b1 haplogroup, but still had relatives (≤ 6th degree) at the site. Among these ten individuals we observe that mtDNA diversity is still relatively low (*h*-value: 0.889), with seven individuals sharing the same haplotype with at least one other. This suggests that additional matrilineal groupings were present at WBK or surrounding communities.

2.8 Y chromosome diversity at Winterborne Kingston

To maximise the amount of data available for Y chromosome haplotype calling at WBK, we relaxed several filters in our read processing pipeline: (1) We did not require an exact P7 index match. A total of two mismatches were allowed across the P5 and P7 index sequences. (2) We included singletons and collapsed reads that required quality trimming. (3) We filtered for a mapping quality above 20 and read length above 30bp. (4) We did not carry out soft-clipping. We used the Pileup tool from GATK v.3.7.0[67] to extract base calls for positions in the ISOGG database of Y chromosomal markers (Version: 15.73, Date: 11 July 2020). We also generated base calls for positions in the "The Big Tree" (ytree.net) database, which provides a draft phylogenetic tree of haplogroup R1b-P312 (Version: March 29, 2021). Base calls below a quality of 30 were removed. Using these markers, we assigned a haplogroup to each sample based on the ISOGG tree, as well as a larger phylogenetic tree reported by YFull (www.yfull.com; v11.04.00). Supporting terminal mutations for YFull clades are provided in Supplementary Table 9. We also provide the TMRCA and the time formed for each YFull clade assigned to our samples, as reported by YFull, which utilises both modern and ancient samples in its phylogenetic inference.

A total of 29 samples were placed within haplogroup R1b-M269, the dominant haplogroup of Western Europe. Ten of these were of a coverage too low to allow for any meaningful downstream assignment. Additionally, two samples placed within the same clade of haplogroup G2a (G2a-Z726), while two placed in different branches of haplogroup I2a1 (I2a1a-Z105 and I2a1b-Y3684).

Of the nineteen R1b-M269 samples with sufficient coverage, eighteen placed within the three major clades of R1b-P312: three in R1b-U152, three in R1b-D27 and twelve in R1b-L21. These three lineages have frequently been associated with historically Celtic-speaking populations in western Europe[68,69] The final sample, a Sub-Roman period individual, placed in a less common branch of R1b-M269, R-S1141.

2.9 Y chromosome diversity in Iron Age Britain

We complied Y chromosome haplogroup assignments for published data from British Iron Age populations[34,41,46,47,50,70]. In the first instance, we took the assignment provided by the publication. These were further subject to sanity checks by implementing the pipeline described in Supplementary Note 2.8. Assignments are summarised in Supplementary Table 18.

Haplogroups were assigned for a total of 203 samples, which fell into the R (n=183), I (n=12), G (n=7) and J (n=1) branches of the human Y chromosome tree. The individual belonging to haplogroup J is a previously identified Middle Eastern outlier from Roman York [47]. The other haplogroups identified are discussed briefly below.

- **Haplogroup I:** Six samples fall within haplogroup I2-L1195, a common haplogroup in Neolithic Britain and Ireland [40]. Given the long-term association of this haplogroup with the islands, it is unsurprising that the six samples that carry it are widely distributed across England and Scotland.

  Three samples belong to haplogroup I2-L160. This haplogroup has not been observed in earlier populations of Britain and Ireland and reaches highest frequencies in populations of the Pyrenees and Sardinia today [71]. Two samples fall within haplogroup I2-S9403, which also has not been observed in pre-Iron Age populations in Britain. One sample falls within haplogroup I1, associated with the Nordic countries.

- **Haplogroup G:** Seven samples fall within the G-Z725 branch of haplogroup G2a, which has not been observed prior to the Iron Age in Britain. It has been previously associated with Central European populations[72].

- **Haplogroup R:** A total of 30 samples were too low in coverage to allow for any meaningful assignment downstream of haplogroup R1b-M269. Of the remaining 153 samples belonging to haplogroup R, 148 were assigned to R1b-P312. Of the remaining five samples, three were assigned to R-U106. R-U106 is not seen in Bronze Age populations from Britain and is associated with Germanic-speaking populations [73]. Notably, two of the R-U106 samples are from a Roman site in York, where genetic outliers with continental ancestry have already been detected[47]. The two other samples (WBK24, I7632) were assigned to rare branches of R1b-L51.

  Of the 148 samples assigned to R1b-P312, 13 could not be placed in a downstream clade. Of the remainder, 109 placed in R1b-L21, eleven in R1b-DF27, eleven in R1b-U152, two in R1b-Z30597, one in R1b-L238 and one in R1b-DF19. L238 and DF19 are very rare in modern populations[74]. Haplogroups R1b-DF27 and R1b-U152 are associated with Iberian and Alpine regions respectively[74], while R1b-L21 is found at peak frequencies in Britain and Ireland today. Ten of our R1b-L21 samples could not be placed in a downstream clade, while the remainder were placed in R1b-DF13 (n=88) and R1b-DF63 (n=11).

Estimates of Y chromosome diversity in ancient populations can be confounded by low coverage and by the use of markers ascertained in modern populations. Both can work to deflate diversity estimates as (1) low coverage samples are typically assigned to upstream clades that are at high frequency in the population (e.g. R1b-P312), and (2) within-clade diversity in prehistoric populations may have been lost in modern populations. To address these issues, we identified the most common major clade of haplogroup R in our dataset, which was R1b-DF13, and removed all samples that could not be placed in a downstream branch of R1b-DF13 or placed in a branch directly ancestral to R1b-DF13 (i.e. samples assigned to R1b-L21, R1b-P312, R1b-M269).

To estimate overall Y chromosome diversity in the British Iron Age population, we then assigned our remaining samples to the most upstream branch of their haplogroup that is represented in the dataset. For example, samples placing in haplogroups G2a2b2a1a1b1a1a, G2a2b2a1a1b1a1a2, G2a2b2a1a1b1a1a2a, G2a2b2a1a1b1a1a2a1a1 and G2a2b2a1a1b1a1a2b, will all be assigned to haplogroup G2a2b2a1a1b1a1a, which is the most upstream branch represented. Finally, we removed one member of all 1st degree pairs. We calculated a *h*-value (see Supplementary Note 2.2) of 0.945 for the British population, indicating a high level of diversity. We note this value is likely an underestimate, given the issues of ascertainment and low coverage mentioned above.

To estimate Y chromosome diversity within archaeological sites, we made use of additional information to improve the accuracy of our estimates. If a sample placed in a clade directly ancestral to another at the site, we checked whether the sample possessed the ancestral allele at markers defining the downstream clade. If so, the samples are coded as having different haplogroups. If not, they are assigned the same haplogroup. While this approach again may artificially deflate diversity values, we find that *h*-values are generally very high across British Iron Age sites (Supplementary Figure 3).

Three sites have values below the island-wide estimate of 0.945. These include two sites (Suddern Farm and Applecross) where no relatives were identified by refinedIBD. In all three cases, the deflation in the diversity value is caused by a single pair of matching haplogroups, all of which formed in the Early Bronze Age. Thus, it is not clear whether these pairs are truly recently related along the male-line.

To further explore the relationship between Y chromosome diversity and relatedness at British Iron Age sites, we followed a similar procedure to our analysis of mtDNA diversity and plotted *h*-values against the normalised number of relative pairs (*rel_norm*) identified using refinedIBD (Figure 2; Supplementary Note 5.3). No correlation was observed (Supplementary Figure 3). Y chromosome diversity at sites with high levels of biological relatedness (>0.25) is similar to the island-wide diversity estimate, implying low levels of male-line relatedness within kin groups and relatively high levels of outward male migration. Y chromosome diversity was also compared to mtDNA diversity, with no correlation observed.

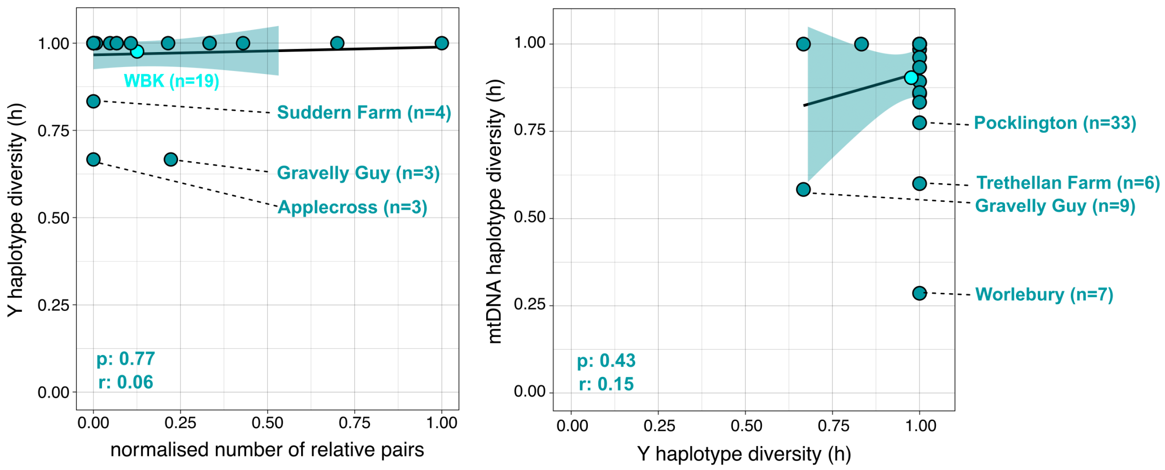

Supplementary Figure 3. Trends in Y chromosome diversity in British Iron Age sites. Haplotype diversity (*h*) was calculated for archaeological sites with two or more individuals following pruning of 1st degree pairs. In the left-hand panel, Y chromosome diversity is plotted against the normalised number of relative pairs seen for each site (1=all pairs are genetic relatives, 0=no pairs are genetic relatives; Supplementary Note 5.3). No correlation is observed, with Y chromosome values staying consistently high across varying values of normalised number of relative pairs. In the right-hand panel, Y chromosome diversity is plotted against mtDNA diversity. Again, no correlation is observed. Outlying sites with lower Y chromosome diversity (left-hand) and mtDNA diversity (right-hand) are highlighted with the sample size used for *h*-value estimation given. WBK is also highlighted.

2.10 Modelling the impact of sex-biased migration on uniparental diversity

The community buried at Winterborne Kingston during the Durotrigian period showed a high level of Y chromosome diversity and a low level of mtDNA diversity, as estimated by the *h*-value. This implies male-biased migration. To estimate the rates of male and female migration required to produce the observed diversity, we used an in-house python script to model the movement of males and females between demes in a population.

The adjustable parameters were:

1. The number of demes (D)
2. The population size of each deme (N)
3. The number of generations (G)
4. The male and female migration rates (M, F)

The fixed parameters were:

1. The number of adult offspring a couple produces is drawn from a normal distribution with a fixed mean of 2.5 and standard deviation of 1. The number sampled from the distribution is rounded to the nearest integer.
2. Demes were required to maintain a minimum of 25 males and 25 females.
3. Sample size for estimating diversity within a deme. We calculated the mtDNA and Y chromosome diversity for demes (*h*-value) using sample sizes of 34 and 17 respectively. These values were chosen to match the number of genetically related WBK individuals with mtDNA and Y chromosome haplotype information.

The simulation works in the following manner.

1. A set of founding individuals is generated, each with a unique uniparental haplotype to reflect the high levels of mtDNA and Y chromosome diversity seen in the British population at large.
2. A round of random mating occurs within demes. A set of N/2 couples is created, rounded to the lowest integer, where N is the population size of the deme. These couples are assigned a set number of offspring, based on the random sampling of a normal distribution. These offspring are randomly assigned a sex.
3. A round of migration occurs between demes. A set of females and males are selected at random from the total population pool to be moved between demes. The number of males and females to be selected for migration is calculated by multiplying the migration rate by the total population size. The destination deme is selected at random for the migrating individuals with the source deme excluded as a possible destination.

4. Steps 2 and 3 are repeated for a set number of generations. Following this, one deme is selected at random. From this deme, a random sample of 34 mtDNAs and 17 Y chromosomes is selected and a *h*-value estimated.

We ran simulations for six different combinations of deme numbers, deme size and generation numbers, varying the rates of male and female migration between 0 and 1. Fifty independent simulations were run for each set of parameters. We compared the resulting *h*-value distributions to those observed at WBK during the Durotrigian period. We estimated two different *h*-values for the Durotrigian population, one based on all individuals and one based on individuals with at least one genetic relative detected. We note that the mtDNA *h*-values for these sample sets are lower than those presented for WBK in Figure 2 and in Supplementary Table 13, as WBK individuals from the Sub-Roman and Early-Middle Iron Age were retained in that analysis.

For both *h*-values and across parameter sets we found our data to be consistent with an outward female migration rate close to zero and a male rate between 0.15-1 (Supplementary Figures 4, 5).

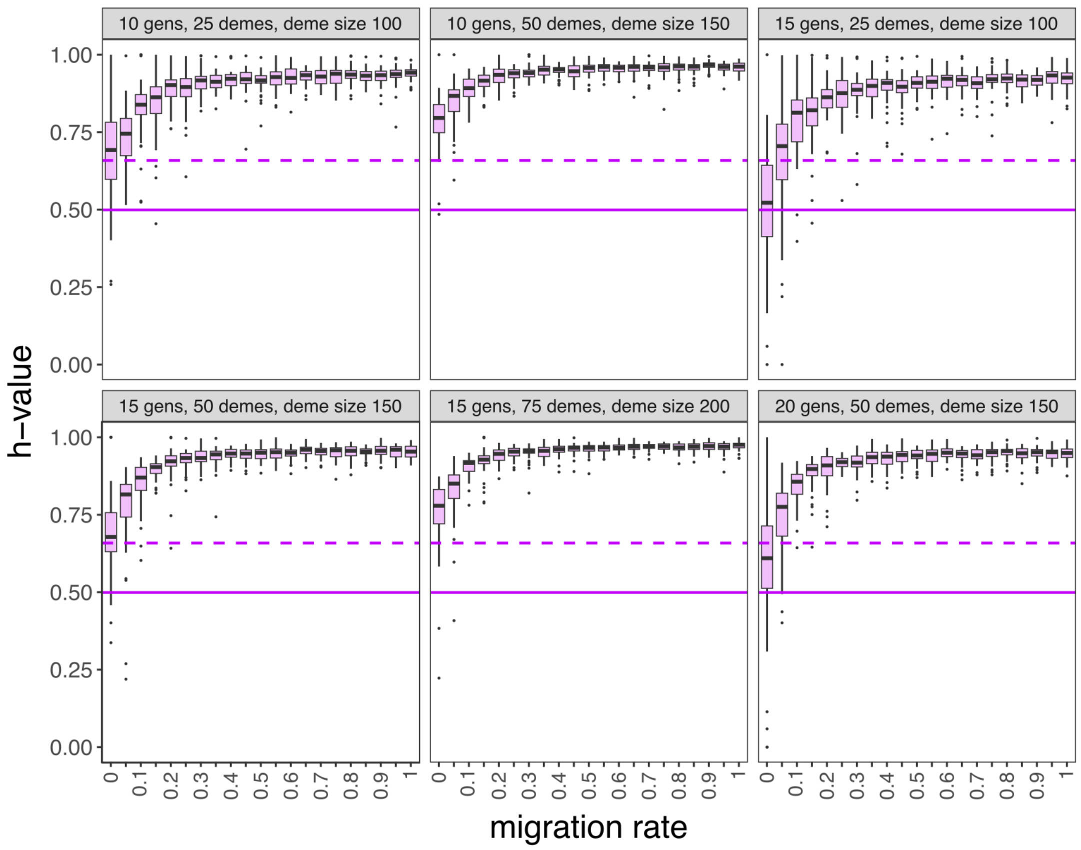

Supplementary Figure 4. The impact of the female migration rate on mtDNA diversity. Simulations are shown for six different combinations of deme numbers (25, 50, 75), deme sizes (100, 150, 200 individuals) and generations (10, 15, 20). For each migration rate, 50 simulations were run. The final *h*-value for a randomly selected deme in each simulation was calculated using a subsample of individuals equal to the sample size of the WBK kin group (n=34). We compare simulated results to two different *h*-values for the population buried at WBK during the Durotrigian period, one based on all individuals (dotted line) and one based on individuals with at least one genetic relative detected (dashed line).

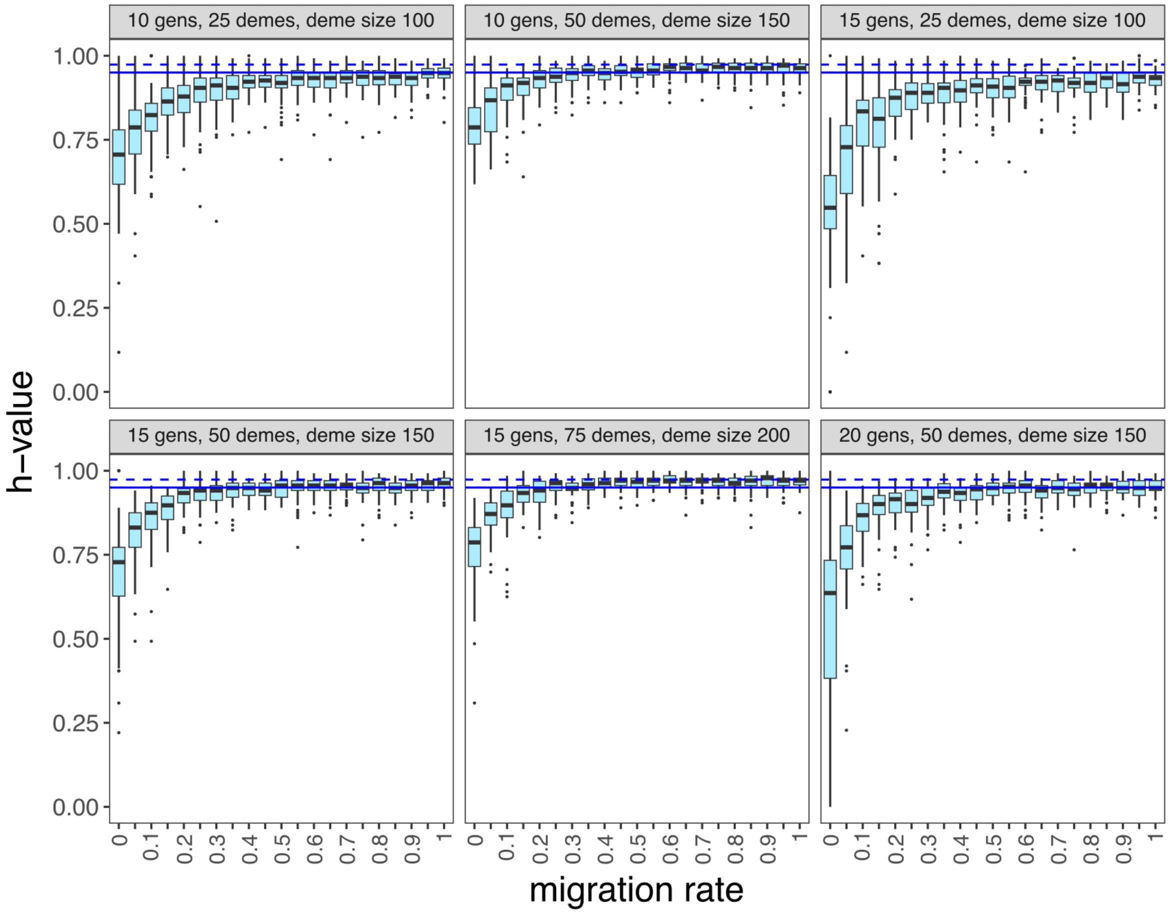

Supplementary Figure 5. The impact of the male migration rate on Y chromosome diversity. Simulations are shown for six different combinations of deme numbers (25, 50, 75), deme sizes (100, 150, 200 individuals) and generations (10, 15, 20). For each migration rate, 50 simulations were run. The final *h*-value for a randomly selected deme in each simulation was calculated using a subsample of males reflective of the sample size of the WBK kin group (n=17). We compare simulated results to two different *h*-values for the population buried at WBK during the Durotrigian period, one based on all individuals (dotted line) and one based on individuals with at least one genetic relative detected (dashed line).

S3. IBD Segment Retrieval for Imputed Ancient Genomes

Four datasets of GLIMPSE imputed diploid genotypes (GP > 0.99) were subject to IBD segment identification (Supplementary Table 12; Supplementary Figure 6). GLIMPSE imputation[75] was carried out on each ancient sample individually prior to being merged into these datasets (see Methods). To identify segments, each of the four datasets was subject to further phasing and imputation using Beagle5[76], followed by refinedIBD analysis[77]. Different sets of variant sites were used as input into both Beagle5 and refinedIBD to test performance and maximise IBD segment retrieval.

3.1 Dataset preparation

WGS1 and WGS+SNPCAP1

The first two datasets consisted of individuals from seven studies[34,39,41,43,46,47,70] that surveyed genetic variation in European Iron Age populations, as well as the new samples sequenced in the current study. We note that additional individuals from these studies (mostly Bronze Age and Medieval Europeans) were retained at this juncture in an attempt to improve Beagle5 phasing and imputation accuracy through increased target sample size. One dataset contained European data generated through both whole genome shotgun sequencing and the 1240k SNP capture (WGS+SNPCAP1), while the other only contained whole genome shotgun data from Britain and Ireland (WGS1).

WGS+SNPCAP2

The third dataset (WGS+SNPCAP2) contained a wider sampling of European individuals through space and time for use in mtDNA diversity analysis (see Supplementary Note 5.3). These were sourced from 18 studies that sampled multi-individual archaeological contexts in Europe[38,40,42,44,45,48–56,78–81]. Three of these studies[51,56,79] included individuals from the same archaeological contexts as samples from WGS+SNPCAP1[34,47,70]. Individuals from these contexts were included in WGS+SNPCAP2 and re-imputed to allow for co-analysis. Please refer to Supplementary Table 12 for further information.

WGS+SNPCAP3

The final dataset (WGS+SNPCAP3) was used for Chromopainter analysis (Supplementary Note 6.3) and included a combination of individuals from WGS+SNPCAP1 and WGS+SNPCAP2 spanning the European Bronze Age to medieval period (Supplementary Table 12). For this dataset, IBD segment retrieval was only used to filter related individuals from ChromoPainter analysis.

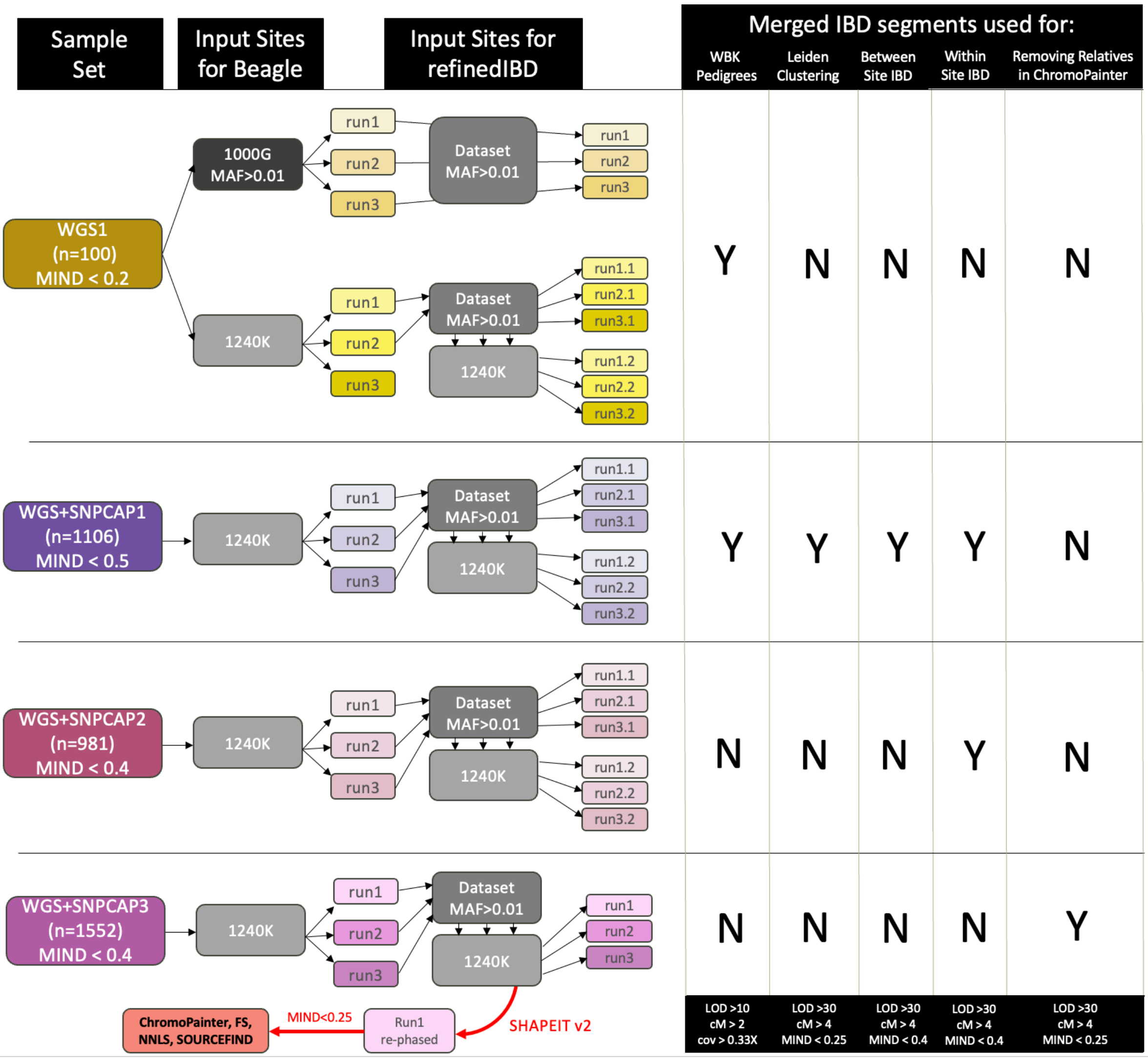

Supplementary Figure 6. IBD segment calling pipeline. Four different datasets (Supplementary Table 12) were subject to IBD segment detection. The WGS1 dataset was further split into two different inputs based on variant sites used. Three independent runs of Beagle phasing and imputation were carried out on each input. The outputs were subject to further variant site filters prior to running refinedIBD. IBD segments across runs were merged and subject to different filters depending on the downstream application (right-hand table). One run of WGS+SNPCAP3 was subject to SHAPEIT phasing and ChromoPainter analysis.

3.2 Imputation

SNP sites and individuals were subject to filters for all datasets. Two different filter sets were used for the WGS1 dataset, while a single filter set was applied to WGS+SNPCAP1, WGS+SNPCAP2 and WGS+SNPCAP3. Each filtered dataset was subject to three independent rounds of Beagle phasing and imputation, as recommended by the creators of the refinedIBD software. The 1000 Genomes Project phase 3 (1000GP) reference panel[82] and genetic maps were downloaded from the Beagle5 website:

http://faculty.washington.edu/browning/beagle/beagle.html

This totalled to 15 Beagle runs - three for WGS+SNPCAP1, three for WGS+SNPCAP2, three for WGS+SNPCAP3 and six for WGS1 (Supplementary Figure 6). More details on the filters applied to each dataset prior to phasing and imputation are given below.

WGS and SNP Capture

For the three datasets containing SNP capture data, we used autosomal positions from the 1240k SNP capture as input into Beagle5. Applying a GP > 0.99 filter to the GLIMPSE imputed genotypes, left a range of missingness values across samples dependent on coverage. For WGS+SNPCAP1, we required a minimum individual missingness (MIND) below 0.5, leaving 1106 individuals for analysis. For WGS+SNPCAP2 and WGS+SNPCAP3, we made this threshold stricter, requiring a MIND below 0.4 prior to Beagle imputation, leaving 981 and 1552 individuals for analysis respectively.

WGS

For the WGS dataset, we used two different sets of filtered sites as input into Beagle.

1. We extracted biallelic autosomal SNP sites with a global MAF > 0.01 in the 1000G Phase 3 dataset prior to Beagle imputation. Any individual with a MIND < 0.2 across these sites was removed. This left a total of 100 individuals and 12,057,261 sites for phasing and imputation.
2. We further extracted the autosomal positions from the 1240k SNP capture from the above set of filtered sites and individuals.

3.3 IBD segment retrieval

Following Beagle phasing and imputation, biallelic SNPs with a MAF above 0.01 in each run were extracted. This left approximately 8 million sites in each run for refinedIBD analysis, which was carried out on the WGS1, WGS+SNPCAP1 and WGS+SNPCAP2 datasets. We also carried out additional refinedIBD runs where we further restricted the input sites to the 1240k positions. This was only done for datasets where 1240k positions were used as input into Beagle. For WGS+SNPCAP3, refinedIBD

was only carried out on this 1240k filtered dataset. This resulted in a total of 21 runs of refinedIBD, all carried out with default parameters. The outputted IBD segments were subsequently subject to different merges and filters depending on the downstream application (Supplementary Figure 6).

3.4 Assessing IBD retrieval rates with parent-offspring pairs

To assess the performance of our IBD segment detection pipeline, we examined the output for eleven parent-offspring pairs from the British Iron Age present in our dataset, eight generated through whole genome sequencing and three through 1240k capture. For each pair, we would expect to retrieve a single IBD1 segment for each chromosome, running the entire length of that chromosome.

The eleven parent-offspring pairs were included in both the WGS1 and WGS1+SNPCAP1 datasets (see previous section). These datasets were subject to different variant site filters before and after Beagle phasing and imputation (Supplementary Figure 6), resulting in five unique datasets for input into refinedIBD, with three replicates of each.

To investigate the impact of sample set and variant sites used, we considered IBD segments from each of the five unique datasets separately, merging segments across the three replicates for each dataset. Before merging segments from replicate runs, we removed small gaps between IBD segments and updated their LOD scores using the merge-ibd-segments.17jan20.102.jar program (default parameters) available on the software's website. These segments were then filtered for a LOD score of 10 or above.

The union of segments across the three replicate runs was then determined with bedtools[83] and centimorgan positions added using an in-house script. We then followed the authors' recommendations for accurate detection of long segments of IBD using refinedIBD and further removed any short gaps (< 2cM) between IBD segments [77]. We also removed short gaps at thresholds of 4cM and 6cM to test performance.

Supplementary Table 20 displays the total number of IBD segments detected for each parent-offspring pair from the British Iron Age. The distributions of these IBD segments across chromosomes 1, 2 and 3 are also shown in Supplementary Figure 7. The main findings are summarised below.

1. **Quality outweighs quantity for input samples and variant sites:** We found the best results were obtained when the 1240k sites were inputted into refinedIBD, rather than a larger set of 8 million sites (dataset MAF >0.01). The 1240k sites were also preferable as input into Beagle5, outperforming the 1000GP MAF >0.01 sites when other variables were held constant (sample set and refinedIBD input sites). This is likely due to higher levels of genotyping and phasing error for 1000GP MAF >0.01 sites. We also found that IBD segment retrieval was improved

for WGS samples when they were imputed alone, rather than within a larger dataset including samples generated through 1240k capture. These results suggest that quality should take precedence over quantity with respect to both the sample set and variant sites used for phasing, imputation and refinedIBD analysis.

2. **Increasing the gap removal threshold to 4cM improves results**: We found a 2cM threshold for merging short gaps between segments was too small to retrieve complete IBD segments for each chromosome for parent-offspring pairs. For the best performing dataset (WGS1→1240k Beagle→1240k refinedIBD), an average of 36 IBD1 segments were identified between parent-offspring pairs at the 2cM threshold. Increasing the threshold to 4cM and 6cM reduced this average to 25 and 23 segments respectively.

3. **Low coverage and 1240k capture samples perform poorly**: The best performance for 1240k capture data was achieved when 1240k sites were used as refinedIBD input and the 6cM threshold was applied (63-120 IBD segments returned). In contrast, the same parameters returned 22-31 IBD segments for shotgun sequence data. The fragmentation of IBD segments between 1240k parent-offspring pairs is visible in Supplementary Figure 7. We also note the effect of sample coverage on IBD detection accuracy, with the poorest performing shotgun pairs containing the lower coverage samples WBK30 (0.44x) and WBK17 (0.76x). The poorest performing 1240k capture pair included the individual with the lowest number of 1240k sites with read coverage (I16416 with 403,740 sites).

Sample Set			WGS+SNPCAP						WGS									Union Across Runs		
Input Beagle sites			1240k						1240k						1000G wmaf01					
Input RefinedIBD sites			1240k			dataset maf01			1240k			dataset maf01			dataset maf01					
**Pair**	**Cov1**	**Cov2**	**2cM**	**4cM**	**6cM**	**2cM**	**4cM**	**6cM**	**2cM**	**4cM**	**6cM**	**2cM**	**4cM**	**6cM**	**2cM**	**4cM**	**6cM**	**2cM**	**4cM**	**6cM**
I12792+ I12793	667k	761k	143	97	68	236	139	78	-	-	-	-	-	-	-	-	-	168	107	69
I16416+ I16503	404k	784k	160	136	120	218	172	139	-	-	-	-	-	-	-	-	-	209	166	134
I19869+ I19870	745k	753k	144	95	63	238	130	76	-	-	-	-	-	-	-	-	-	166	94	56
WBK05+ WBK06	1.84x	1.16x	**29**	**22**	**22**	56	**27**	**22**	31	**25**	**22**	60	**25**	**23**	243	111	66	**23**	**22**	**22**
WBK05+ WBK16	1.84x	1.26x	34	**25**	**23**	60	**26**	**24**	32	**25**	**24**	57	**25**	**23**	261	117	66	**26**	**23**	**22**
WBK15+ WBK22	10.0x	0.84x	41	30	26	79	38	**27**	33	**23**	**23**	65	32	**26**	295	158	95	**28**	**22**	**22**
WBK15+ WBK30	10.0x	0.45x	65	37	31	128	48	33	41	**27**	**24**	80	33	**26**	301	191	125	33	**23**	**22**
WBK17+ WBK14	0.76x	0.90x	60	39	30	114	49	33	49	34	**26**	95	42	**27**	279	141	87	40	**25**	**23**
WBK19+ WBK22	1.83x	0.84x	36	**25**	**22**	59	36	26	31	**25**	**22**	54	28	**24**	236	106	63	**26**	**22**	**22**
WBK30+ WBK19	0.45x	1.84x	55	34	29	91	45	30	34	**27**	**24**	77	35	**27**	289	189	110	35	**27**	**24**
WBK31+ WBK22	0.78x	0.84x	43	**24**	**23**	74	37	25	32	**24**	**22**	69	34	**23**	253	125	74	30	**23**	**23**

Supplementary Table 20. Number of autosomal IBD segments detected between parent-offspring pairs for different datasets. An optimal value of 22 segments is expected. Values between 22-25 are shown in bold. Beagle5 was run independently three times on three distinct datasets. Two different site filters were applied prior to refinedIBD analysis (dataset MAF >0.01 or dataset MAF >0.01 + 1240k). IBD1 segments were merged across the three replicate runs and gaps between segments were merged using a 2cm, 4cm and 6cm threshold. The best performance for WGS data was seen for WGS_1240k with 1240k sites inputted into refinedIBD. Coverages are given as number of thousand sites covered in the 1240k panel for SNP capture samples and whole genome coverage (x) for shotgun sequenced samples.

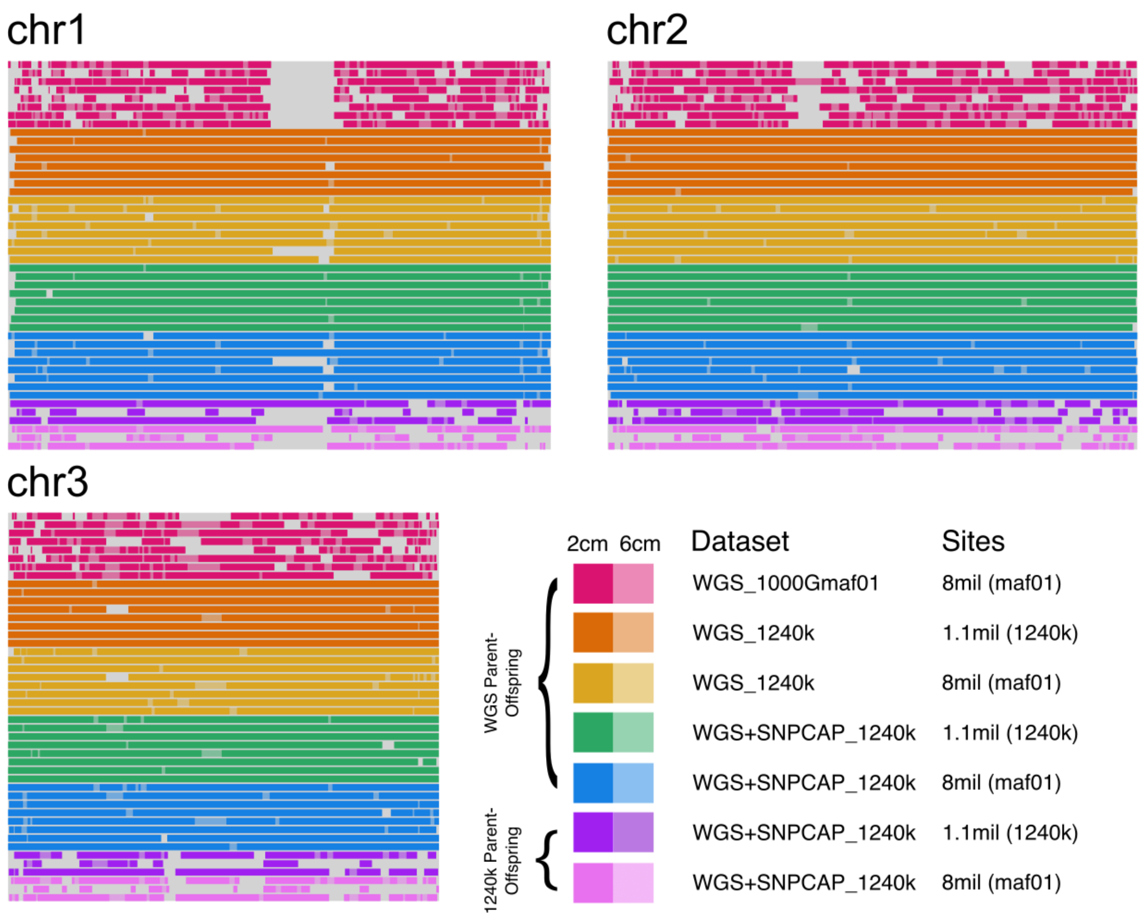

Supplementary Figure 7. IBD segment retrieval for parent-offspring pairs. IBD segments are plotted with respect to physical position on the chromosomes (bp). Beagle5 was run independently three times on three distinct datasets (WGS_1000Gmaf01, WBK_1240k, WBK+SNPCAP_1240k). Two different site filters were applied prior to refinedIBD analysis (a dataset MAF >0.01 or dataset MAF >0.01 + 1240k). For the WGS+SNPCAP_1240k dataset, different colours are assigned to the eight WGS pairs (green and blue) and three SNPCAP pairs (pink and purple). Dark colours represent IBD1 segments retrieved using a 2cM threshold, with lighter colours indicating the extent of IBD sharing when a 6cM threshold is used.

3.5 IBD segment identification on the X chromosome

For X chromosome IBD segment retrieval, we used the same samples present in the WGS1 (n=100) and WGS+SNPCAP1 (n=1106) datasets after MIND filtering. However, GLIMPSE imputed diploid genotypes were only used for female samples (GP>0.99). For males, haploid calls were generated by randomly choosing a single base (BQ>30) covering the position. These data were merged and sites in the pseudoautosomal regions of the X chromosome removed.

As for autosomal analysis, we tested the performance of different sets of variant sites used as input for Beagle and refinedIBD (Supplementary Table 21). A total of nine unique datasets were used as input for Beagle, with three replicate runs carried out on each (27 runs total). These included transversions-only datasets to reduce the confounding effects of post-mortem damage on male haploid calls. Further site filters were applied prior to running refinedIBD, resulting in 63 refinedIBD runs total.

X chromosome IBD segments were treated similarly to autosomal segments. Before merging segments from replicate runs, we removed small gaps between IBD segments and updated their LOD scores using the merge-ibd-segments.17jan20.102.jar program (default parameters) available on the software's website. These segments were then filtered for a LOD score of 10 or above. The union of segments across the three replicate runs was then determined with bedtools[83]. However, we did not carry out any further removal of short gaps for the X chromosome, as was done for the autosomes. Instead, to test performance, we simply calculated the proportion of base pairs in IBD for each parent-offspring pair from the British Iron Age. Only shotgun sequence pairs were considered. No IBD segments were found for the single father-son pair (WBK05 and WBK06), as to be expected.

A high variance in IBD retrieval is seen between different Beagle5 runs for the X chromosome. The worst performing datasets were those that used 1000GP MAF > 0.01 sites with both transitions and transversions retained. Here, shared IBD segments were found to cover only 1-22% of the X chromosome, with pairs containing a male individual performing substantially worse (0-2%). The best performing datasets were those that used 1240k sites as input into Beagle5. As was seen for autosomal tests, shotgun sequenced samples performed significantly better when imputed alone using 1240k sites as input, rather than alongside 1240k capture samples. The best performing Beagle run recovered IBD1 segments for 81-96% of the X chromosome.

When we merged segments across independent runs, we achieved near complete IBD coverage of the entire X chromosome for all pairs (97-100%)

Sample Set	Input Beagle sites	Input refined IBD sites	WBK05+ WBK16	WBK14+ WBK17	WBK19+ WBK30	WBK15+ WBK30	WBK15+ WBK22	WBK19+ WBK22	WBK22+ WBK31
		dataset maf01	0.02	0.01	0.01	0.01	0.06	0.20	0.12
	1000G wmaf01	dataset maf01 (tvs)	0.13	0.05	0.06	0.08	0.14	0.29	0.21
		dataset maf01	0.17	0.14	0.18	0.10	0.24	0.38	0.32
	1000G wmaf01 (tvs)	dataset maf01 (tvs)	0.24	0.22	0.23	0.13	0.28	0.49	0.41
		dataset maf01	0.04	0.03	0.04	0.03	0.15	0.33	0.20
	Dataset maf05	dataset maf01 (tvs)	0.19	0.09	0.16	0.10	0.21	0.38	0.27
		dataset maf01	0.37	0.24	0.19	0.21	0.32	0.50	0.50
	Dataset maf05 (tvs)	dataset maf01 (tvs)	0.41	0.30	0.28	0.25	0.40	0.57	0.59
		dataset maf01	0.69	0.57	0.45	0.49	0.56	0.61	0.57
		dataset maf01 (tvs)	0.80	0.73	0.55	0.54	0.63	0.67	0.68
	1240k	1240k	0.82	0.66	0.53	0.55	0.57	0.60	0.69
		dataset maf01	0.74	0.68	0.52	0.52	0.59	0.59	0.61
		dataset maf01 (tvs)	0.79	0.64	0.55	0.59	0.63	0.63	0.62
WGS+SNPCAP (1106 samples)	1240k tvs	1240k	0.86	0.72	0.60	0.61	0.53	0.53	0.58
		dataset maf01	0.01	0.01	0.00	0.01	0.12	0.21	0.20
	1000G wmaf01	dataset maf01 (tvs)	0.11	0.04	0.08	0.11	0.17	0.32	0.32
		dataset maf01	0.16	0.18	0.14	0.17	0.19	0.40	0.34
	1000G wmaf01 (tvs)	dataset maf01 (tvs)	0.21	0.28	0.18	0.25	0.27	0.47	0.49
		dataset maf01	0.88	0.80	0.81	0.83	0.90	0.96	0.93
		dataset maf01 (tvs)	0.88	0.80	0.81	0.83	0.90	0.96	0.93
WGS Only (100 samples)	1240k	1240k	0.89	0.78	0.70	0.69	0.77	0.79	0.93
Totals			0.99	0.97	1.00	0.97	0.98	0.98	0.98

Supplementary Table 21. Proportion of X chromosome IBD sharing detected between shotgun sequenced parent-offspring pairs for different datasets. Beagle5 was run independently three times on nine distinct datasets. Two different site filters were applied prior to refinedIBD analysis (dataset MAF >0.01 or dataset MAF >0.01 with transition sites removed). IBD segments were merged across the three replicate runs and gaps between segments were merged using a 2cm, 4cm and 6cm threshold. The best performance for WGS data was seen for WGS_1240k with 1240k sites inputted into refinedIBD.

S4. Resolving Pedigree Relationships at Winterborne Kingston

To reconstruct familial relationships at Winterborne Kingston we used a combination of data types:

1. mtDNA and Y chromosome haplogroup sharing (Supplementary Note 2)
2. Autosomal coefficients of relatedness that were calculated using both allele-frequency-based methods and identical-by-descent (IBD) segment sharing
3. IBD1 and IBD2 segment numbers and lengths
4. Longest observed IBD segments within the genome
5. X chromosome IBD segment sharing

These data are summarised in Supplementary Table 10 and discussed in turn below.

4.1 Coefficient of relatedness

Two methods were used to calculate the coefficient of relatedness (r) between each pair of Winterborne Kingston individuals i and j: an allele frequency based approach[84], which provided an r^{α} estimate, and an IBD segment-based approach, which we refer to as the r^{β} estimate[77]. The latter approach has previously been found to be one of the most accurate methods for relatedness estimation[85], inferring 7th degree relatives correct to within one degree of relatedness for over 76% of pairs in a large modern dataset using approximately half a million SNPs.

Allele matching

First, we calculated the coefficient of relatedness using an allele-pair matching method[84], which we refer to here as r^{α}. For the Winterborne Kingston samples, we called pseudo-haploid genotypes at biallelic autosomal SNP sites with a global minor allele frequency (MAF) > 0.01 in the 1000G Phase 3 dataset. We further filtered the dataset for sites with a minor allele count above 3 in our newly sequenced samples. We then used the --genome function from PLINK[86] to calculate the proportion of alleles that are identical by state (IBS) for each pair of individuals (DST value output from PLINK). This value can be denoted as M_{ij}. We then calculated the median proportion of alleles that are identical in state for all pairs of individuals in the dataset, denoted as M_s. The coefficient of relatedness between each pair of individuals, which we denote as r^{α}_{ij}, was then defined as follows:

$$r_{ij}^{\alpha} = \frac{M_{ij} - M_s}{1 - M_s}$$

For sample pairs where only allele matching estimates of the coefficient of relatedness were available, we did not attempt to infer relationships beyond the 3rd degree and only considered pairs with at least 50,000 shared sites for comparison.

IBD segment sharing

The coefficient of relatedness (r^{β}) was also calculated by taking the proportion of the genome in centimorgans (cM) that is shared between pairs of individuals. Only individuals with a genomic coverage above 0.3x were considered, given the poorer performance of lower coverage samples in our IBD retrieval tests (Supplementary Note 3).

Estimations of r^{β} for Winterborne Kingston individuals, as well as other Iron Age populations, had previously been calculated using the WGS+SNPCAP1 dataset, which contained the union of IBD segments from across 6 refinedIBD runs (Supplementary Note 5.2; Supplementary Table 14). This identified 108 putative pairs of relatives at the site of approximately the 8th degree or higher. To further investigate these relationships, we carried out an additional 9 refinedIBD runs on a panel of whole genome sequenced individuals (Supplementary Note 3; Supplementary Figure 6). By taking the union of autosomal segments from across this total of 15 refinedIBD runs, we improved our ability to retrieve intact IBD1 segments that spanned the length of each chromosome for parent-offspring pairs (Supplementary Note 3).

Calculation of r^{β} deviated slightly from the approach described in Supplementary Note 5.2. Here, our main aim was not to detect relatives, but to retrieve intact IBD1 and IBD2 segments between known relative pairs of approximately the 7th degree or higher. Thus, we used a more relaxed LOD score and segment length filter. We also removed short gaps between segments, as recommended by the software's authors when accurate detection of long IBD segments is required[77].

1. For IBD1 regions, small gaps were removed between IBD segments in each of the fifteen runs using the merge-ibd-segments.17jan20.102.jar program (default parameters) and segments filtered for a LOD score of 10. The union of these segments for each pair of individuals across all fifteen runs was then determined with bedtools[83] and centimorgan positions added using an in-house script.

2. For IBD2 regions, we did not merge gaps to begin with. Instead, we filtered for a LOD score of 10 and extracted regions where individual pairs shared two IBD segments within the same run (i.e. IBD2 regions). We then took the union of these IBD2 regions across the fifteen runs using bedtools[83] and added centimorgan positions with an in-house script. Given the higher false positive rates for IBD2 regions, we removed IBD2 segments below one cM in length at this juncture.

Finally, for both IBD1 and IBD2 segments, we removed short gaps (< 4cM) and then removed any remaining segments below 4cM in length. The coefficient of relatedness (r^{β}) was then calculated using the following formula:

$$r_{ij}^{\beta} = \frac{IBD1_{ij} + IBD2_{ij}}{2 \times total\ genome\ length}$$

Here, the total genome length considered was 3545.83 cM.

For posterity, we also calculated r^{β} for segment length thresholds of 2cM and 8cM (Supplementary Table 10).

4.2 A note on sample WBK195

Samples WBK191-195 were excavated in the summer of 2023 and were not sequenced until after IBD-sharing analysis was completed. Allele-matching analysis revealed WBK195 to have multiple relatives fitted within the pedigree. To further validate our findings, we replicated the WGS1 and WGS+SNPCAP1 refinedIBD runs exactly (Supplementary Notes 3.1-3.3), including sample WBK195. We also replicated the X chromosome IBD analysis (Supplementary Note 3.5) with this sample included. We note that the version of WBK195 used for pseudo-haploid analysis is of lower coverage and WBK195 was not included in haplotypic analyses detailed in Supplementary Notes 5 and 6.

4.3 Estimating the degree of relationship

The degree of a relationship can be defined using the length of the shortest ancestral path (or paths) joining two individuals in a pedigree, measured in parent-child links. A parent and child will have a 1st degree relationship (one parent-child link). Parents will share the entirety of their autosomal genome in IBD1 with their offspring (i.e. half of their total autosomal DNA). Siblings can also be considered 1st degree relatives, as they are linked by two paths of two parent-child links. This could be conceived as a double 2nd degree relationship, which is the equivalent of a 1st degree relationship. However, as siblings are linked through two different ancestral paths, they are expected to have a different IBD sharing profile than parents and offspring. Specifically, their IBD segments will not often encompass whole chromosomes, as multiple rounds of recombination have occurred, and they are expected to share IBD2 regions (summing to approximately a quarter of the genome), as both individuals are related through both their parents (Supplementary Figure 8).

The expected amount of IBD shared between individuals is halved with each degree of distance, from which an expected coefficient of relatedness can be calculated (1st: 0.5, 2nd: 0.25, 3rd: 0.125, etc.). This manifests as fewer and shorter IBD segments shared with each degree of distance. However,

recombination introduces stochasticity and thus the ranges of observed relatedness coefficients can overlap between different degrees of relationship. These overlaps become larger with each degree of distance. For this reason, when estimating the degree of a relationship, it is useful to consider the number and average length of IBD segments shared between a pair of relatives, rather than just the total amount of IBD shared. IBD length distributions can also be useful in distinguishing between different forms of 2nd and 3rd degree relationship. This is due to variation in the number of meioses that separate different types of relative and the higher recombination rate in female gametes. For example, on average a paternal grandchild and father will share fewer IBD segments than an avuncular pair, but these segments will be longer on average than those of an avuncular pair.

Taking the above considerations into account, we estimated the degree of relatedness for each pair of individuals in two different ways.

1. Relatedness coefficient thresholds derived using powers of 2

These are non-overlapping upper and lower thresholds for defining the degree of the relationship from the relatedness coefficient[87]:

- 1st: 0.3536-0.7072
- 2nd: 0.1768-0.3536
- 3rd: 0.0884-0.1768
- 4th: 0.0442-0.0884
- 5th: 0.0221-0.0442
- 6th: 0.0110-0.0221
- 7th: 0.0055-0.0110

These thresholds are generally consistent with simulations and were utilised in a benchmarking study of relatedness inference methods, which demonstrated the high accuracy of the approach[85]. We used the r^{β} value where possible (4cM segment length threshold), to classify each pair up to the 7th degree. This resulted in a single degree value estimated for each pair (Supplementary Table 10). However, we stress this value is an estimate. For low coverage pairs, the r^{α} value was used up to infer relatives to the 3rd degree, but not beyond.

2. Simulated IBD length distributions

The above thresholds do not account for overlaps in relatedness coefficient ranges for different degrees of relationship. To account for this, we used ped-sim to simulate relationships of different degrees[88] with the sex-specific genetic maps[89] and interference parameters[90] available on the software's website (see Supplementary Table 11 for summary statistics). We simulated all possible simple pedigree relationships from 1st to 4th degree. For relationships beyond the 1st degree, we consider a simple relationship one of the following:

- direct ancestor-descendent
- a single ancestral path through one shared ancestor (half-siblings, half-cousins, half-avuncular)
- two ancestral paths that converge on a pair of full siblings (full cousins, avuncular).

For 5th to 8th degree, we restricted simulations to all possible simple male- and female-line relationships, as it was not practical to model all possible genealogical paths. Importantly, as there is a higher recombination rate in female gametes, male- and female-line relationships represent the expected extremes of 5th to 8th degree autosomal IBD sharing. For each simulated relationship we ran 1000 replicates. We then plotted the total number of IBD1 segments against the total length of the genome in IBD1 (cM) for each pair, applying a 4cM segment length filter (Supplementary Figure 9). As expected, we observe overlaps between the simulated length distributions for different degrees of relatedness. These distributions were then compared to our data from Winterborne Kingston, allowing us to identify the range of relatedness degrees that fit the observed IBD sharing for each WBK pair of related individuals. Only WBK pairs with an $r^{\beta} > 0.0055$ (4cM length threshold) were considered. This allows us to classify ambiguous relationships accordingly (e.g. 3rd-4th rather than simply 4th degree). These classifications are presented in Supplementary Table 10 and Supplementary Figure 9.

We find all WBK pairs fall within simulated ranges, a testament to the accuracy of our pipeline for IBD segment retrieval. We further assess the possibility that our results are skewed by a large number of short false positive segments by considering the length distribution of IBD segments between two pairs of WBK siblings. We plot the expected distribution of IBD1 segment lengths for siblings (1000 simulations) and the observed distribution for two WBK sister pairs and find no inflation in short segments in our observed data (Supplementary Figure 10).

Final set of relatives

From the combined approaches described above we identified 114 pairs of relatives at WBK, ranging from approximately the 1st-7th degrees. This included 46 pairs of close relatives at WBK - 11 pairs of 1st, 8 pairs of 2nd and 27 pairs of 3rd-4th degree relatives. We also identified 68 pairs of more distant relatives - 32 pairs of 4th-7th degree relatives and 36 pairs of the 5th degree or beyond (Supplementary Table 10). These comprised 34 individuals from WBK. All of those with direct radiocarbon dates placed within the Durotrigian period of the site's usage. We note our allele matching and IBD approaches (740 pairs subject to both) show excellent concordance for close relatives. All relatives classified as 3rd degree and 4th degree with allele matching fall within the observed IBD ranges for those degrees.

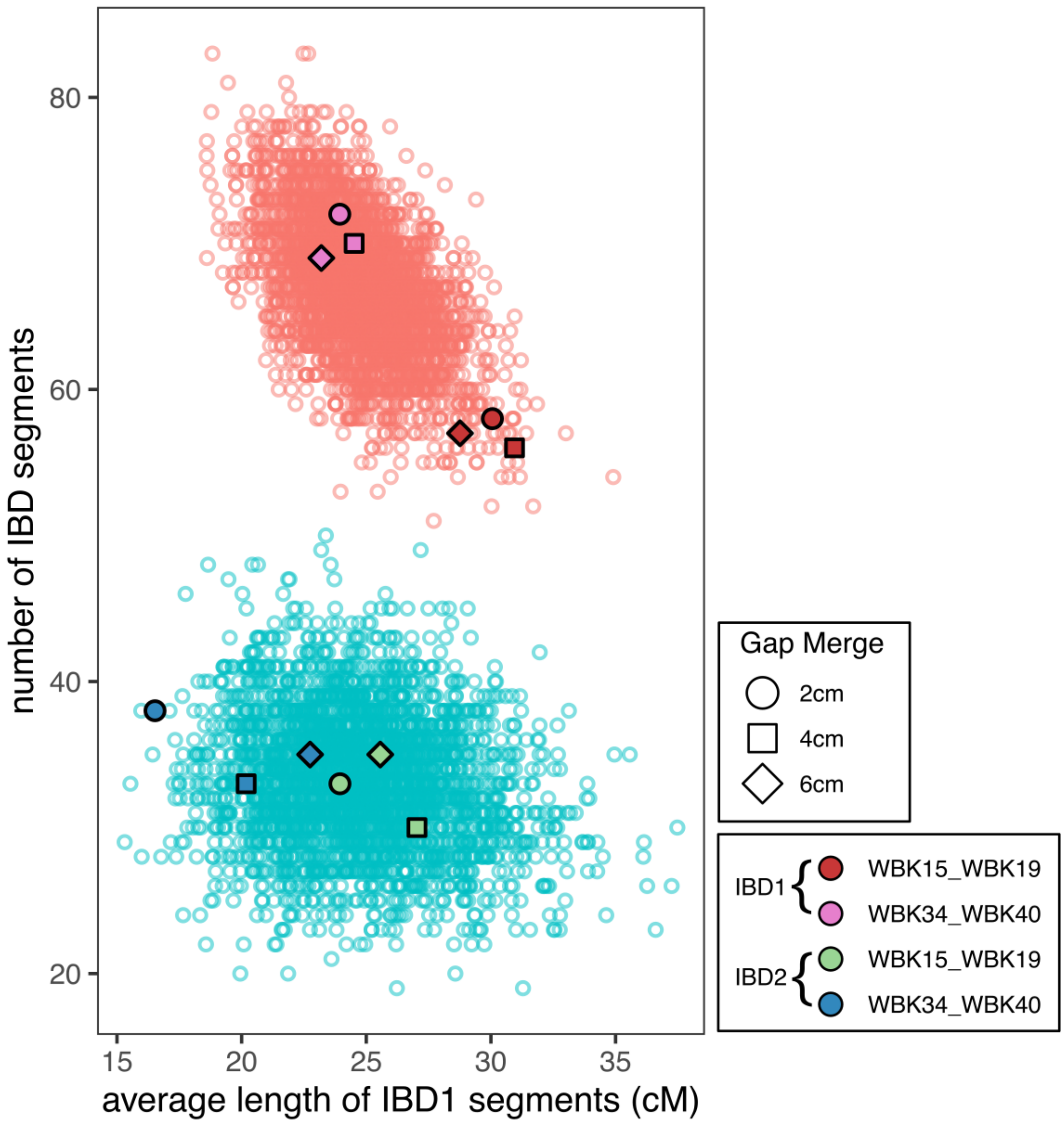

Supplementary Figure 8. Accuracy of IBD1 and IBD2 retrieval for WBK sibling pairs. The number of autosomal IBD segments is plotted against the average segment length (cM) for 3000 simulated sibling pairs (hollow circles) and observed data for two pairs of sisters from Winterborne Kingston (black outline). IBD1 segments are represented in red/pink and IBD2 in green/blue. Results for three different gap removal thresholds are plotted for the sisters (<2cM, <4cM and <6cM).

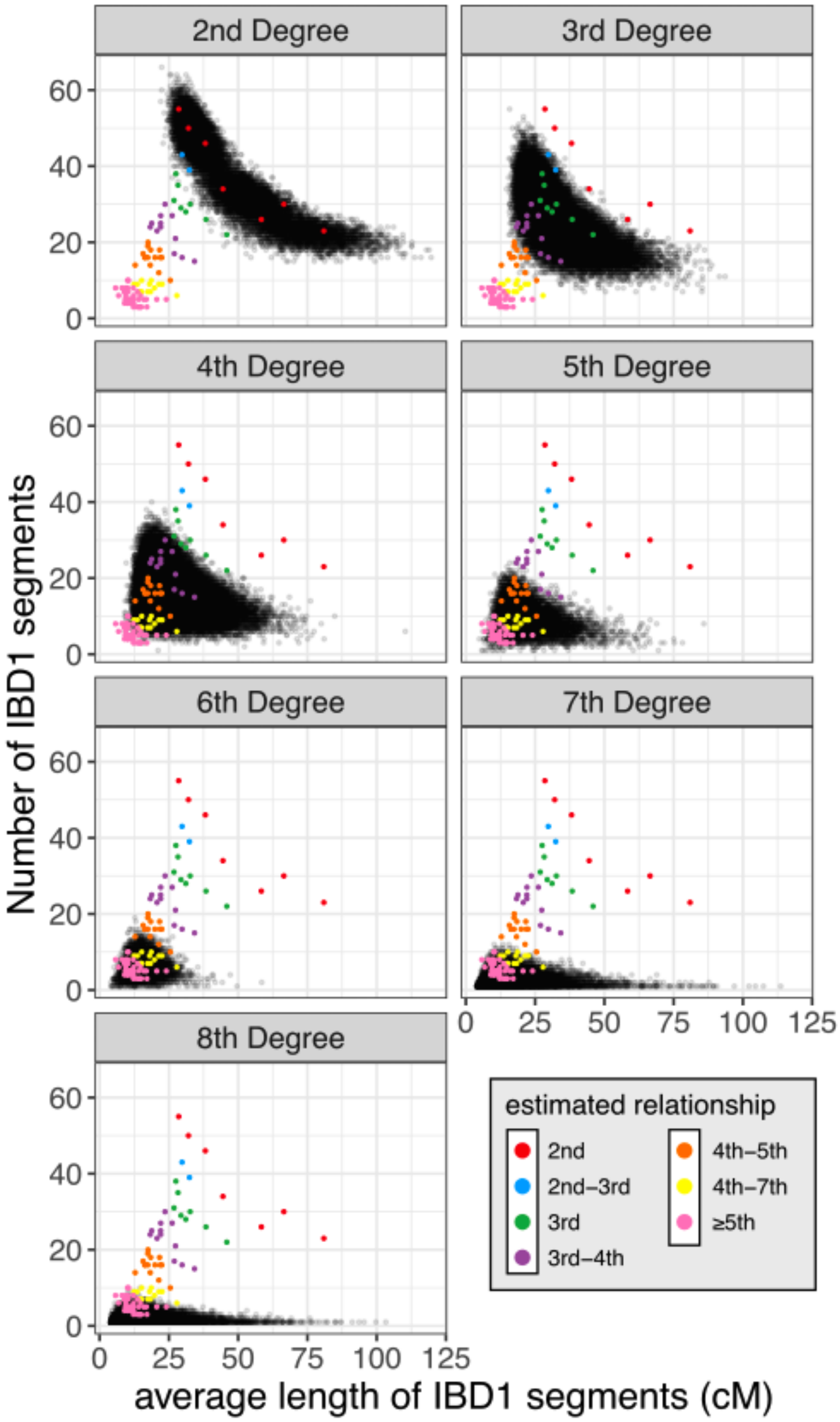

Supplementary Figure 9. Simulated and observed IBD length distributions. Simulated ped-sim data is shown in black. For each type of relationship (multiple types for each degree) 1000 replicates were run. The number of autosomal IBD segments (< 4cM) is plotted against the average segment length (cM) for all WBK pairs with an r^{β} value greater than 0.0055 (4cM minimum segment length, 4cM gap removal threshold). WBK pairs are coloured based on their estimated relationship, which is inferred from their positioning on the plot.

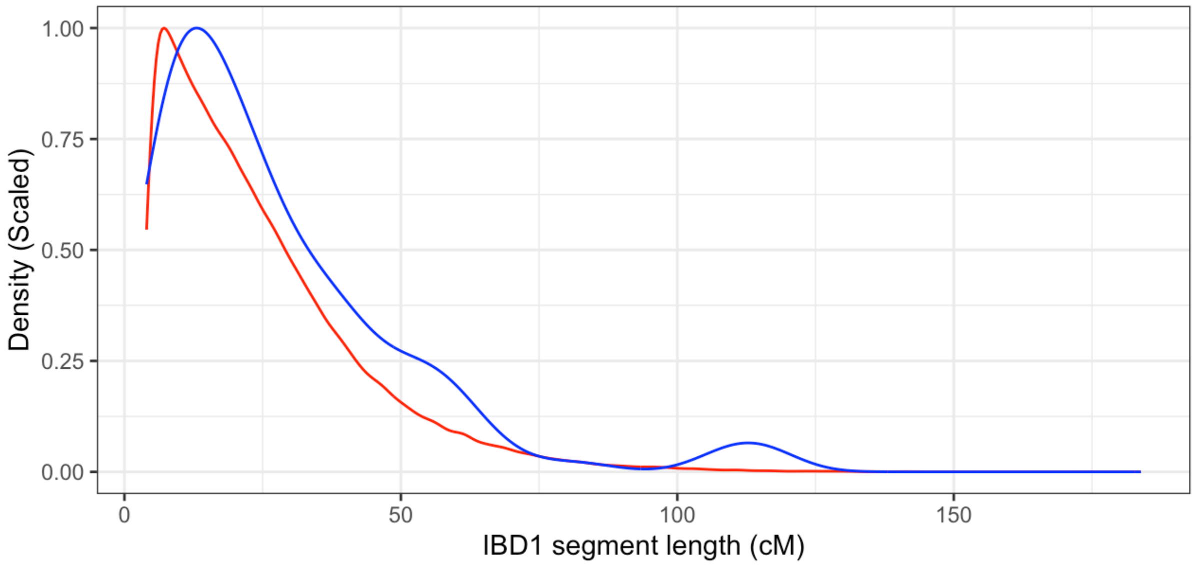

Supplementary Figure 10. Expected and observed IBD length distributions for siblings. Simulated (red) and observed (blue) IBD1 segment length distributions for sibling relationships are shown. Note that simulated data is averaged across 1000 runs, while observed (WBK) data is based on two sibling pairs.

4.4 IBD2 segment retrieval

Importantly, the set of IBD1 regions we identified for each sample pair using refinedIBD (see above) may also include IBD2 regions. To allow for comparison with the output of ped-sim, we subtracted IBD2 regions from the set of IBD1 regions using bedtools[83] for Winterborne Kingston siblings pairs and two 2nd degree relatives pairs with high levels of IBD2 (125-200 cM). We then removed any resulting IBD1 segments with a length below 4cM. To test the accuracy of our IBD retrieval for individuals with high IBD2, we compared distributions for our two full sibling pairs (WBK15+WBK19; WBK34+WBK40) to simulated data (Supplementary Figure 8). The number of comparable simulations here was 3000, as we simulated brothers, sisters and mixed siblings separately for downstream comparisons. We present results for the <4cM gap removal threshold used for estimation of r^{β}, as well as <2cM and <6cM thresholds for posterity. We find that values for both pairs fall within the simulated distributions.

4.5 X chromosome IBD sharing and simulations

The number and length of X chromosome IBD segments shared between each pair of Winterborne Kingston individuals was also calculated. We took the union of segments shared across 63 refinedIBD runs (Supplementary Note 3.5). Small gaps were first removed within each run using the merge-ibd-segments.17jan20.102.jar program (default parameters) and segments filtered for a LOD score of 10. The union of these segments for each pair of individuals across all 63 runs was then determined with bedtools[83] and centimorgan positions added using an in-house script. We then removed short gaps (< 4cM) between segments and removed any remaining IBD segments below 2cM in length. The results are provided in Supplementary Table 10. Parent and offspring comparisons suggest high accuracy for this approach, inferring >0.97 of the X chromosome in IBD for 7 male-female and female-female pairs from Winterborne Kingston, with no segments identified for the single father-son pair, WBK05 and WBK06 (Supplementary Note 3.5).

We observed a relatively large amount of X chromosome IBD sharing between individuals with high autosomal relatedness ($r^{\beta} > 0.0422$) at Winterborne Kingston, with approximately 60% of pairs sharing more than 8 cM. This could be indicative of a lack of male-male transmissions within the community. To test whether this observation reflects the residence pattern at WBK, we simulated three six-generation pedigrees whose members practised 1) patrilocality, 2) matrilocality and 3) mixed residence. These pedigrees were simulated with ped-sim as follows:

1. A single founder individual and spouse comprise the 1st generation. This couple has two daughters and two sons (2nd generation). In the patrilocal simulation, only the sons reproduce.

In the matrilocal simulation, only the daughters reproduce. In the mixed residence simulation, one child of each sex reproduces. Spouses are unrelated individuals who enter the community.

2. Each reproducing member of the 2nd generation has two daughters and two sons (8 individuals total in the 3rd generation). Four of these individuals reproduce with unrelated spouses to form the next generation. The sex of the offspring who reproduce again depends on the residence pattern (patrilocal: four sons, matrilocal: four daughters, mixed: two sons and two daughters).
3. This process is repeated until the 6th generation is reached.

For each couple, we allow the two non-reproducing offspring to remain in the community. These non-reproducing individuals were simulated to reflect the fact that a fraction of the migrating sex will be buried in their natal communities (e.g. individuals who did not marry or died before reaching marriageable age, marriages between members of the same community). Each pedigree comprised a total of 246 individuals (including spouses).

After running the simulations, we removed autosomal IBD segments below 4cM and X chromosome segments below 2cM to mirror the filters used for IBD calling in the WBK dataset. We then extracted all pairs of approximately 4th degree or closer ($r^{\beta} > 0.0422$) from our simulated pedigrees. For each pair, we calculated the X chromosome kinship coefficient (Xθ) and autosomal kinship coefficient (θ). The X chromosome coefficient was calculated by taking the total length of IBD shared (cM) and dividing by:

- The total length of the X chromosome for male-male pairs
- Twice the total length of the X chromosome for female-male pairs
- Four times the total length of the X chromosome for female-female pairs

To approximate the WBK kin group, we randomly subsampled our simulated pairs to consist of the same number of 4th, 3rd, 2nd and 1st degree pairs as classified by refinedIBD for WBK. We also required the same number of male-male, female-male and female-female pairs within these categories. We carried out this random subsampling 100 times on our matrilocal, patrilocal and mixed residence pedigrees. We then plotted the distribution of Xθ/θ for all simulations and for the WBK kin group (Supplementary Figure 11). As expected, we saw the distribution of Xθ/θ ratios in patrilocal simulations to skew towards zero. This is because a large number of male-male transmissions are expected in patrilocal societies, which results in individuals with high autosomal relatedness but little or no X chromosome relatedness. We saw the opposite effect in matrilocal simulations, while the mixed residence simulations fell in between these two extremes. The WBK distribution falls within the range seen for matrilocal simulations, implying a smaller number of male-male transmissions than expected for a mixed residence or patrilocal community.

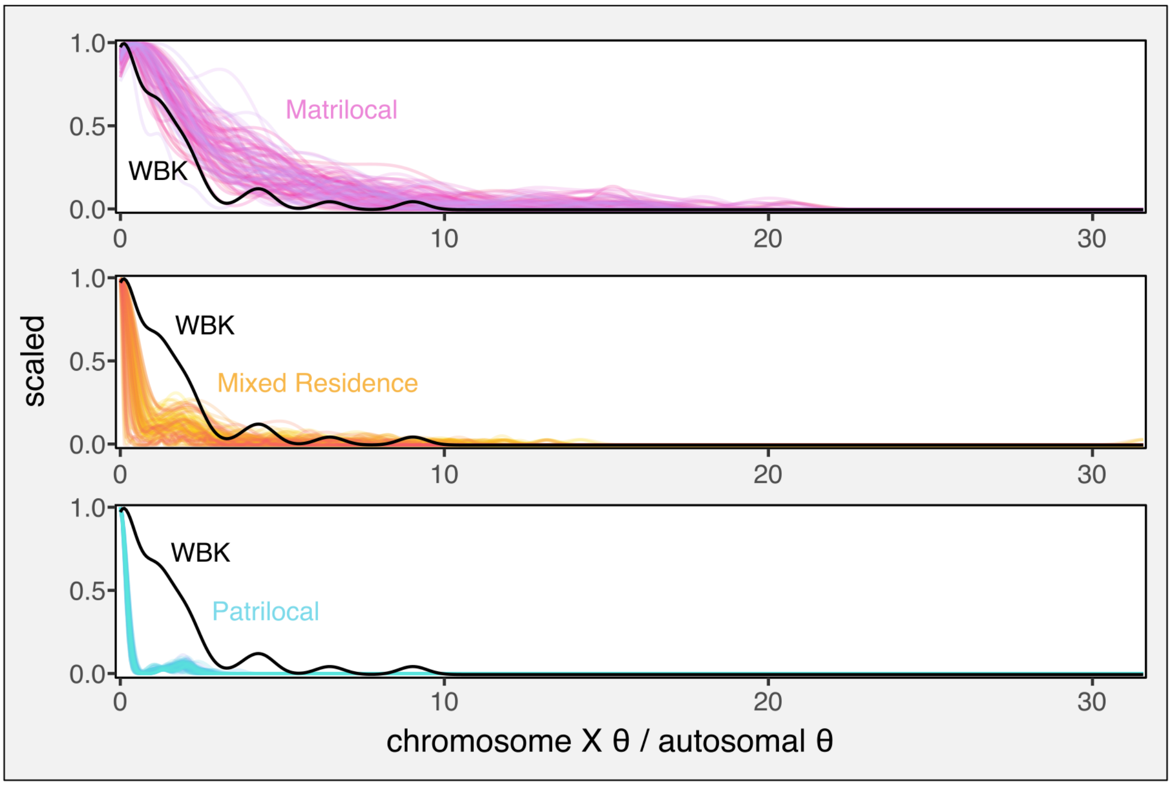

Supplementary Figure 11. Distribution of the X chromosome to autosomal kinship coefficient for close relatives WBK and simulated residence patterns. Here, we define a close relative as those who share an autosomal r^{β} >0.0422. Six-generation pedigrees (number of individuals: 246) for three different residence patterns were simulated with ped-sim and randomly subsampled 100 times for a set of 46 close relative pairs that reflect the WBK sample set (degree of relationship and sex composition of pairs). The distribution of the ratio of the X chromosome to autosomal kinship coefficient was plotted for each simulation and WBK, with the WBK distribution falling within the range seen for the matrilocal simulation.

4.6 Resolving 1st degree relationships

In total, we identified 11 pairs of 1st degree relatives, 10 of which contained individuals of high enough coverage to include in imputation and IBD sharing analysis. IBD analysis allowed us to differentiate between siblings (>680 cM IDB2 shared) and parent offspring pairs (<70 cM IBD2 shared). Two pairs of sisters were identified, but no brothers or mixed sibling pairs. Nine parent-offspring pairs were identified. We provide information on the classification of each below.

WBK15 and WBK19: Sisters

This was determined based on IBD2 sharing (Supplementary Figure 8).

WBK34 and WBK40: Sisters

This was determined based on IBD2 sharing (Supplementary Figure 8).

WBK30 and [WBK15, WBK19]: Father and daughters

The pair of sisters, WBK15 and WBK19, do not share an mtDNA haplotype with the male WBK30. Thus, WBK30 must be their father.

WBK22 and [WBK15, WBK19]: Mother and daughters

The pair of sisters, WBK15 and WBK19, show a level of IBD2 sharing with the female WBK22 that demonstrates a parent-offspring relationship. As WBK22 cannot be the daughter of both and as she is not related to the sisters' father WBK30, she must be their mother.

WBK16 and WBK05: Mother and son

The male WBK05 shows a level of IBD2 sharing with the female WBK16 that demonstrates a parent-offspring relationship. As they share a mitochondrial haplogroup, WBK16 must be WBK05's mother. She cannot be his daughter, as she is unrelated to WBK06, who is another 1st degree relative of WBK05.

WBK06 and WBK05: Father and son

WBK05 and WBK06 do not share an mtDNA haplotype and thus must be father and son. As WBK06 is unrelated to WBK16, the mother of WBK05, he must be the father of WBK05.

WBK17 and WBK14: Father and daughter

WBK14 and WBK17 do not share an mtDNA haplotype. Thus, the male WBK17 must be the father of the female WBK14. This is consistent with the level of IBD2 sharing.

WBK31 and WBK22: Mother and daughter

The female WBK31 shows a level of IBD2 sharing with the female WBK22 that demonstrates a parent-offspring relationship. We further identify WBK31 as the mother of WBK22. This conclusion is based on patterns of IBD sharing between WBK31 and the daughters of WBK22 (WBK15+WBK19), which is discussed further in the following section.

WBK39 and WBK33: Father and son or son and father

This pair of male individuals was found to have a 1st degree relationship based on allele-matching analysis. They do not share an mtDNA haplotype and thus they must be father and son. However, with the current data we are unable to resolve who is the father and who is the son.

4.7 Resolving 2nd degree relationships

We identified 8 pairs of 2nd degree relatives, all of which contained individuals of high enough coverage to include in imputation and IBD sharing analysis.

We also observed one pair of males, WBK06 and WBK23, who were classified as 2nd-3rd degree relatives on the basis of IBD length distributions (Supplementary Figure 9, Supplementary Table 10). This pair had a borderline relatedness coefficient (r^{β}=0.1804, r^{α}=0.1551, 2nd degree lower threshold of r=0.1768). They were subsequently found to be 3rd degree relatives (see below).

Two pairs of 2nd degree relatives showed a substantial amount of IBD2 sharing (WBK17 and the sisters WBK34 and WBK40), implying a double relationship (e.g. double cousins), which is examined further in the next section. To narrow down the relationships between the remaining six pairs, we compared the number and total length of IBD1 segments shared between them to simulated data for all possible non-double 2nd degree relationships (Supplementary Figure 12).

WBK31 and [WBK15, WBK19]: Maternal grandmother and granddaughters

The pair of sisters, WBK15 and WBK19, are the daughters of WBK22. WBK31 is their 2nd degree relative and a 1st degree relative of WBK22. Therefore, WBK31 must be the sisters' maternal grandmother or their maternal half-sister. The IBD sharing between both sisters and WBK31 falls within the range seen for a grandparent-grandchild relationship and far outside that of maternal half-siblings (Supplementary Figure 12). Thus, we can conclude that WBK15 and WBK19 are the granddaughters of WBK31.

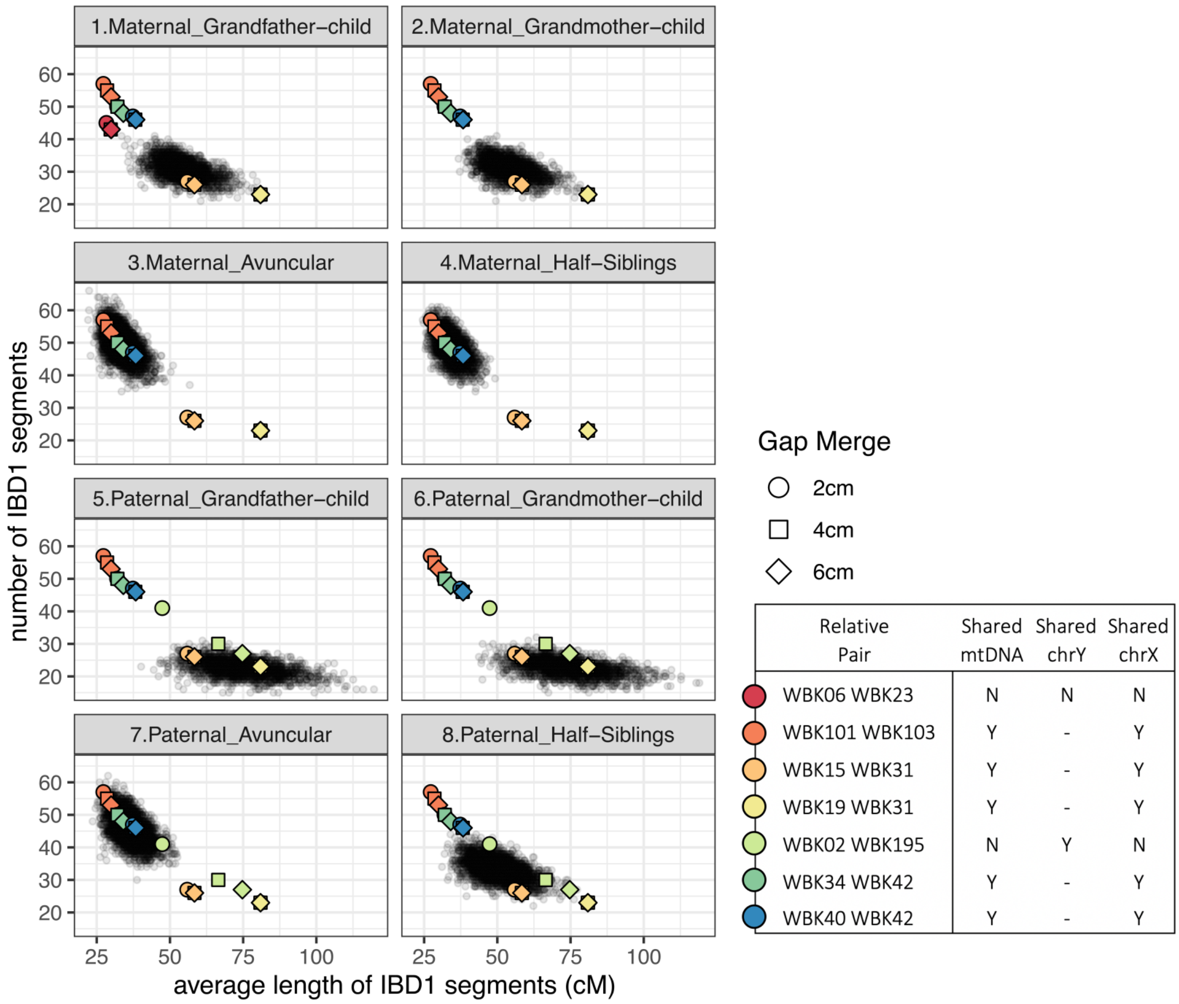

Supplementary Figure 12. Resolving 2nd degree relationships with IBD segments. Simulated ped-sim data is shown in black (1000 replicates for each scenario). The number of autosomal IBD segments is plotted against the average segment length (cM) shared for putative 2nd degree relatives at Winterborne Kingston. Pairs are not plotted in panels for simulated relationships inconsistent with their mtDNA, X and Y chromosome sharing. WBK06 and WBK23 were subsequently found to be 3rd degree relatives, in keeping with their positioning outside of the simulated distribution for maternal grandfather and child, the only 2nd degree relationship that would lead to no shared mtDNA or Y chromosome haplotype. We present results for both the <4cM gap removal threshold, used for estimation of r^{β}, as well as the <2cM and <6cM thresholds using different symbols.

WBK101 and WBK103: Avuncular or maternal half-siblings

These are a male (WBK101) and female (WBK103) individual, neither of whom have any other close relatives identified at the site. Both belong to the dominant matrilineage of the site but share no private mutations. Thus, given the frequency of couplings between individuals of the same matrilineage in this community, we cannot assume these two are recently related through the female line. However, as a large fraction of the X chromosome is shared in IBD between them, we can rule out a paternal grandparent-grandchild relationship, paternal half-siblingship, and WBK103 being the paternal aunt of WBK101.

We can also rule out a maternal grandparent-grandchild relationship from the number of IBD segments shared (Supplementary Figure 12). Thus, the relationship is either maternal avuncular, maternal half-siblingship or WBK101 is the paternal uncle of WBK103. We note that if this is a paternal avuncular relationship it would require another coupling between a male and female of the same matrilineage.

WBK06 and WBK23

These two males do not share mtDNA or Y chromosome haplotypes and so the only possible relationship is maternal grandfather and grandson. However, the pair share no IBD segments on the X chromosome. They also do not show the pattern of autosomal IBD segment sharing expected for maternal grandparent and child (Supplementary Figure 12). Thus, we conclude that these individuals are in fact 3rd degree relatives, which fits with the borderline coefficient of relatedness (r^{β}=0.1804, $r\alpha$=0.1551) estimated for this pair. Their exact relationship is further deduced by identifying additional 3rd and 4th degree relatives of these individuals (see next section).

WBK42 and [WBK34, WBK42]: Sororal niece and aunts

The sisters WBK34 and WBK40 are both 2nd degree relatives of WBK42. This immediately rules out WBK42 as a grandchild of either of the sisters. Patterns of IBD-sharing further exclude all grandparent-grandchild relationships and paternal half-siblingship.

Thus, the relationship must be avuncular or maternal half-siblingship. We note that if this is a paternal avuncular relationship it would require another marriage between a male and female of the same matrilineage.

Patterns of autosomal and X chromosome IBD sharing allow us to narrow down this relationship further and conclude that WBK42 is most likely the sororal niece of the sisters WBK34 and WBK40. This is based on the following observations:

1. The sisters share different sets of X chromosome segments with WBK42. Thus, WBK42 cannot be the sisters' paternal aunt (Supplementary Figure 13).
2. If the sisters are WBK42's paternal aunts, we would expect any X chromosome region they both share in IBD1 with WBK42 to be in IBD2 between them. This is because the sisters share an entire X chromosome inherited from their father, while any brother of WBK34+WBK40 would only share IBD1 segments with them inherited from their mother (males do not inherit their father's X chromosome). Thus, any segments shared with WBK42 would also have to derive from the sisters' mother. However, we observe a large region that is in IBD1 between WBK42 and both sisters, but only in IBD1 between the sisters.
3. WBK42 shares 143cM of the X chromosome in IBD1 with WBK40. Across 1000 simulations, only 2% of paternal aunt-niece pairs share above 140 cM on the X chromosome and only 2.1% of maternal half-sister pairs. In contrast 19% of maternal aunt-niece pairs share above 140 cM. Thus, a maternal aunt-niece relationship is approximately 10 times more likely than maternal half siblingship or a paternal aunt-niece relationship
4. Patterns of autosomal IBD sharing indicate it is unlikely that WBK42 is the aunt of the sisters, based on a previously outlined approach[91]. Specifically, if WBK42 was the sibling of the sisters' father or mother, we would expect WBK42 to share approximately 25% of their genome in IBD2 with the sisters' unsampled parent. From this, we would expect >50 cM of the regions where both sisters are IBD1 with their aunt to be IBD0 between the sisters (i.e. the sisters inherited different haplotypes in regions where their parent was in IBD2 with their aunt). However, we only identify 12 cM total of these tentative $IBD^{011}_{sis1,sis2,aunt}$ regions, with only two regions above 2 cM in length. These are likely the result of inaccurate IBD segment endpoint determination, rather than WBK42 being the true aunt of the sisters.

WBK02 and WBK195: Paternal grandfather and grandson

This pair share a rare Y chromosome haplotype, but not an mtDNA haplotype, ruling out a maternal 2nd degree relationship. Thus, they must have a paternal half-sibling, paternal avuncular or paternal grandfather-son relationship. When the total length and number of IBD segments shared is considered, the pair fall between data simulated for paternal half-siblings and paternal grandfather and grandson (4 cM merge; Supplementary Figure 12). Their exact relationship is further deduced by fitting them in a pedigree with WBK20, their shared 3rd degree relative (see Supplementary Note 4.9).

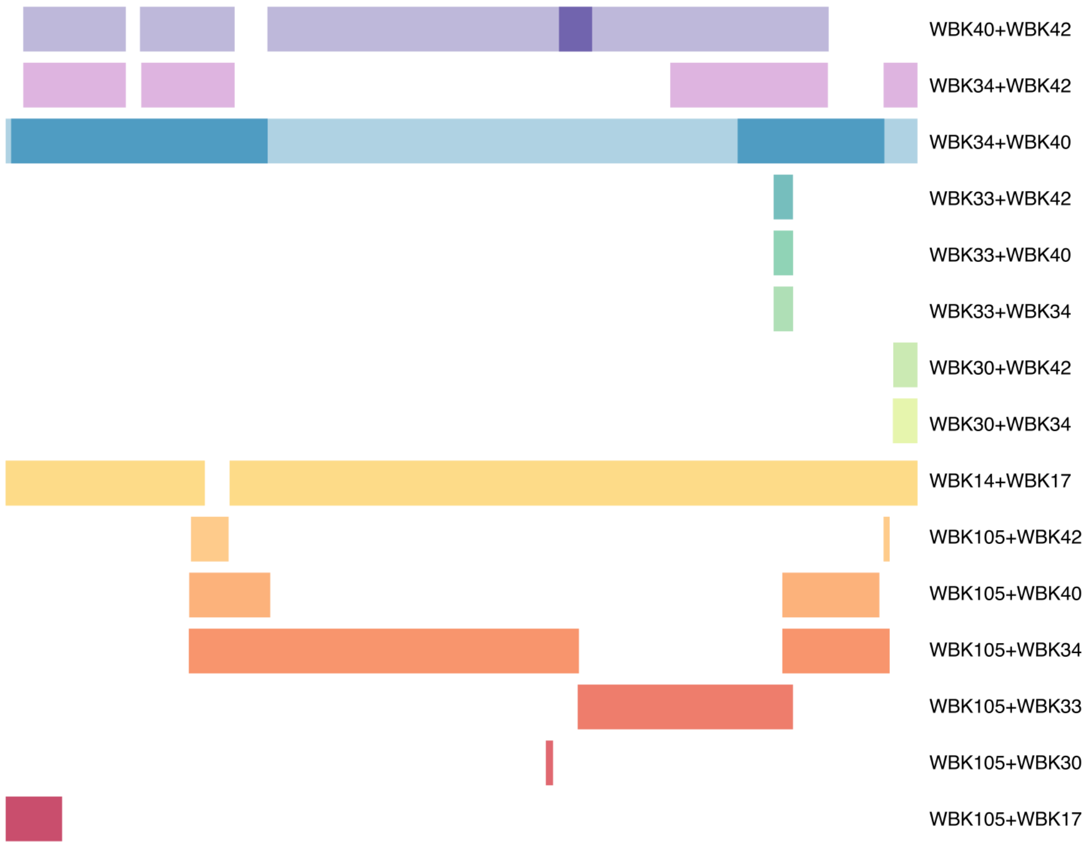

Supplementary Figure 13. X chromosome IBD segments shared between the sisters WBK34+WBK40 and their relatives. IBD segments are plotted with respect to physical position along the X chromosome. Dark colours represent IBD2 segments and light colours IBD1 segments.

4.8 Resolving a 2nd degree double relationship

WBK34+40 and WBK17: Double half-avuncular

We noted a substantial amount of IBD2 sharing between WBK17 and his two 2nd degree relatives, WBK34 and WBK40 (a pair of sisters). There is no evidence for recent inbreeding within the genomes of these individuals when homozygous-by-descent segments output by refinedIBD are considered (Supplementary Note 5.5). This implies a double relationship (e.g. double cousins). Double relationships occur when two individuals are related through both their parents. Double relationships of the 2nd degree occur when two sets of 1st degree relatives have offspring (e.g. a pair of sisters marrying a pair of brothers). To investigate, we used ped-sim to simulate all possible 2nd degree double relationships, listed in Supplementary Table 22.

Both WBK34 and WBK40 share fewer IBD1 segments with WBK17 than expected for a double cousin or cousin-half-avuncular relationship (Supplementary Figure 14). Instead, our observations best fit with a double half-avuncular relationship. These occur when a parent-offspring pair mates with another parent-offspring pair (e.g. a father and son have offspring with a mother and daughter). There are five types of double half-avuncular relationships, involving either cross-generation or same-generation pairings (Supplementary Table 22; bottom row of Supplementary Figure 14). When we consider the possible placements of the male WBK17 and sisters WBK40+WBK34, this results in eight unique pedigree structures.

RelativePair1		RelativePair2		
MatePair1	MatePair2	MatePair1	MatePair2	Offspring relationship
Brother	Brother	Sister	Sister	Double cousins (type 1)
Brother	Sister	Sister	Brother	Double cousins (type 2)
Brother	Sister	Daughter	Father	Cousins and half-avuncular (type 1)
Brother	Brother	Mother	Daughter	Cousins and half-avuncular (type 2)
Sister	Sister	Father	Son	Cousins and half-avuncular (type 3)
Brother	Sister	Mother	Son	Cousins and half-avuncular (type 4)
Mother	Son	Son	Mother	Double half-avuncular (type 1)
Father	Daughter	Daughter	Father	Double half-avuncular (type 2)
Father	Son	Daughter	Mother	Double half-avuncular (type 3)
Father	Son	Mother	Daughter	Double half-avuncular (type 4)
Father	Daughter	Mother	Son	Double half-avuncular (type 5)

Supplementary Table 22. Types of double 2nd degree relationship. These happen when a pair of 1st degree relatives produce offspring with another pair of 1st degree relatives. Offspring can have a double cousin relationship (they are cousins twice over), a cousins and half-avuncular relationship (they are cousins through one pair of parents and half-niece/nephew and half-aunt/uncle through the other pair), or a double half-avuncular relationship (they are half-niece/nephew and half-aunt/uncle twice over). These can come in different forms, which are numbered, depending on the sexes and generational sequence of the 1st degree pairs.

We also considered IBD sharing on the X chromosome. Neither WBK34 nor WBK40 were found to share any X chromosome IBD segments with WBK17, which is unexpected for a double relationship. We used ped-sim to determine the probability of such an event occurring for the eight different pedigree structures. Two pedigrees had a probability of <1%, while three had a probability >5% (Supplementary Figure 14).

To further narrow down the possible relationship between the sisters and WBK17, we considered the male individual WBK105, who is a 3rd degree relative to both WBK17 and the sisters. He also shares large blocks of IBD on the X chromosome with all three individuals. This means that in any double avuncular pedigree, WBK105 must be a cousin of WBK17 and a half-nephew of the sisters through his mother or vice versa (cousin of the sisters and half-nephew of WBK17).

All possible placements of WBK105 in each double avuncular pedigree are shown in Supplementary Figure 15. All but four of these can be eliminated due to incorrect patterns of X chromosome sharing. Specifically, the sisters WBK34+WBK40 cannot be related to WBK105 through their father, as they share different sets of X chromosome segments with WBK105 (Supplementary Figure 13). Daughters inherit a complete X chromosome from their father and thus should show identical patterns of X chromosome IBD sharing with his relatives (pseudoautosomal regions excluded). WBK17 also cannot be related through his father to WBK105, as they share X chromosome regions in IBD and men do not transmit their X chromosome to their sons.

Of the four remaining possibilities, two involve the coupling of a pair of individuals whose children from other partners (i.e. stepchildren) also produce offspring themselves (type 4). The other two possibilities involve a mother-daughter pair mating with a father-son pair, where the mother and son produce offspring and the father and daughter produce offspring (type 3). We deem the latter less anthropologically and biologically plausible, given the generation gaps required. Thus, we present the former scenario in our most parsimonious pedigree for Winterborne Kingston (Fig. 1a). However, we note that the use of the latter would not change any other aspects of the pedigree, such as the placement of WBK105 and WBK42. Of the two possible type 4 configurations, we chose the pedigree with the higher probability of WBK17 and the sisters sharing no X chromosome segments (7.7%).

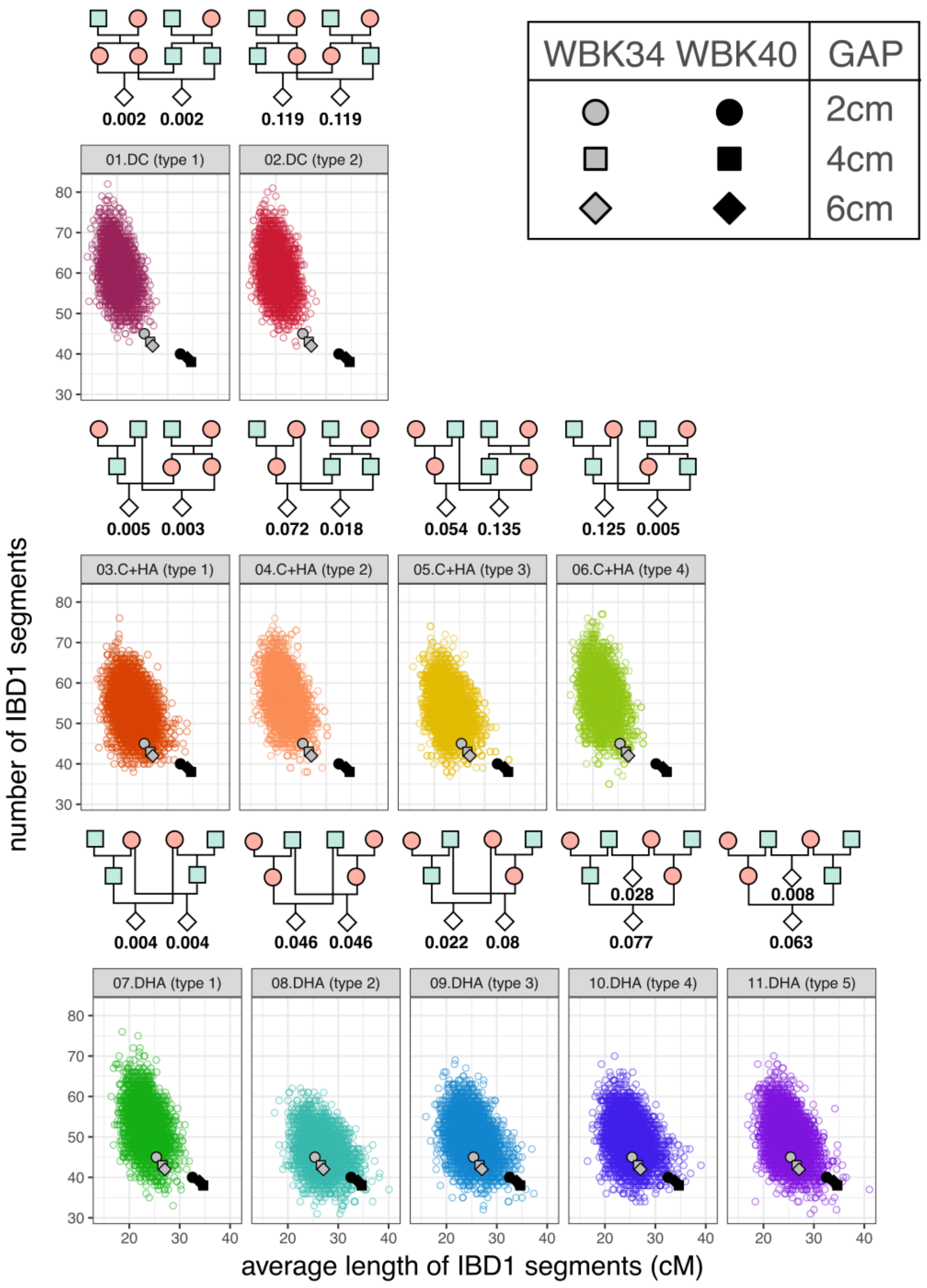

Supplementary Figure 14. Resolving a 2nd degree double relationship with IBD segments. Simulated ped-sim data is plotted separately for all eleven types of double 2nd degree relationship (1000 replicates for each scenario). The number of autosomal IBD1 segments is plotted against the average segment length (cM) in each panel, with the corresponding pedigree shown above: circles - females; squares - males. White diamonds denote the possible placement of WBK17 and WBK34+WBK40. The probability of WBK17 sharing no X chromosome IBD segments with both sisters if placed at that position is shown underneath each diamond. Note that switching the positions of WBK17 and the sisters does not impact pedigree structure or X chromosome transmission for some relationships (e.g. double cousins). We present results for the <2cM, <4cM and <6cM thresholds using different symbols. Data points for gap merges >4cM only fall within the range seen for double half-avuncular relationships. (DC: Double-Cousin, C+HA: Cousin and Half-Avuncular; DHA: Double Half-Avuncular)

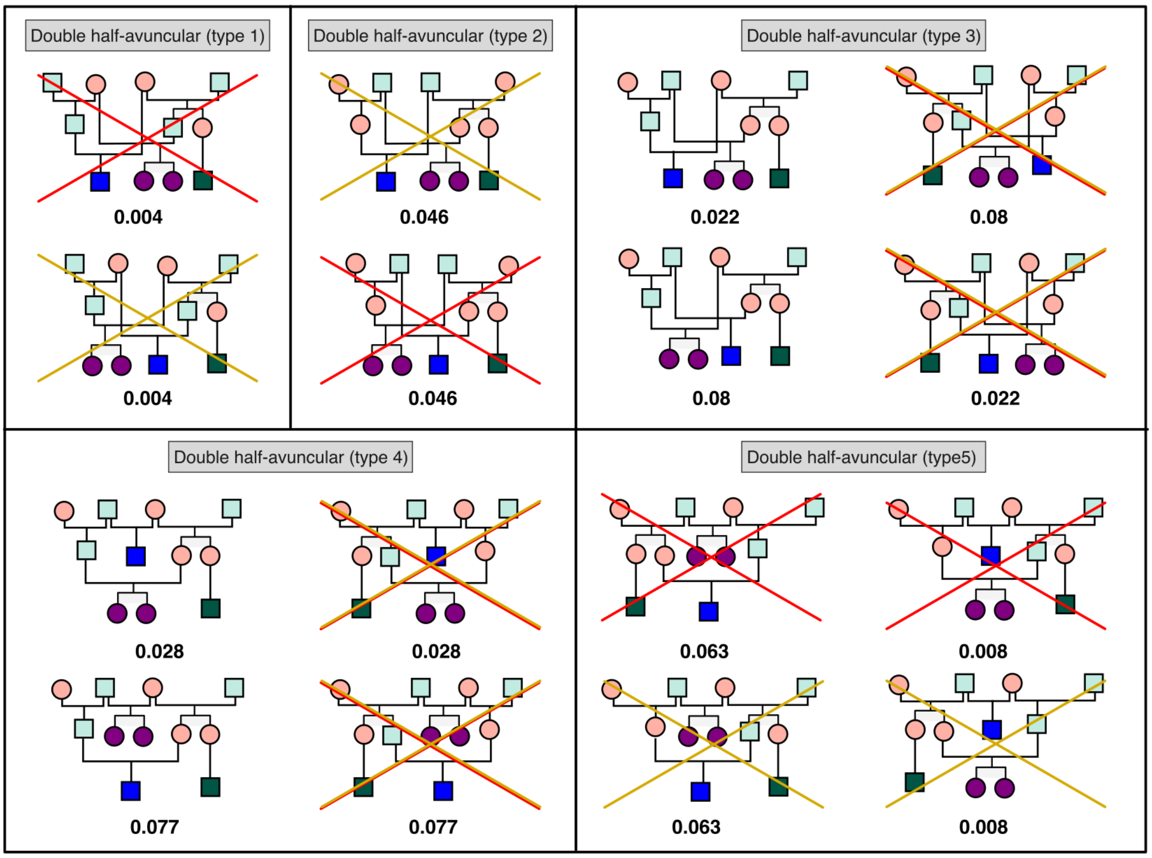

Supplementary Figure 15. Possible pedigree relationships between WBK105, WBK17 and the sisters WBK34 and WBK40. There are eight unique double avuncular pedigrees to consider. For each, WBK105 (green square) is positioned as either a cousin of the sisters (purple circles) through his mother in the top pedigree or the cousin of WBK17 (blue square) in the bottom pedigree. The probability of WBK17 sharing no X chromosome IBD segments with both sisters if placed at that position is shown underneath each pedigree. Pedigrees that do not agree with the observed data are crossed out. A red cross is used if these sisters are related to WBK105 through their father, as this would require them to show identical patterns of X chromosome IBD sharing with WBK105, which they do not. A yellow X is used if WBK17 is related to WBK105 through his father, as the two males share X chromosome segments and thus must be related through their mothers.

4.9 Resolving 3rd degree relationships

We could securely identify nine pairs of 3rd degree relatives based on the total number and length of IBD1 segments (Supplementary Figure 9). We further identified twelve pairs of 3rd-4th degree relatives based on IBD sharing, seven of whom could be classified as 3rd or 4th degree on the basis of their placement in the pedigree. Three additional pairs were estimated to be 3rd degree relatives on basis allele-matching.

The nine secure pairs included WBK14, the daughter of WBK17, and her 3rd degree relative WBK34 (a 2nd degree relative of WBK17). We can be confident that WBK14 is also a 3rd degree relative of WBK40, WBK34's sister, although this relationship was classified as 3rd-4th degree based on IBD sharing. Another three secure pairs contained WBK105, and his 3rd degree relatives, WBK17, WBK34 and WBK40, discussed in the previous section. We further narrow down the relationship between the remaining five secure pairs of 3rd degree relatives below.

WBK31 and WBK12: Maternal-maternal great-grandmother and great-grandson

WBK12 and WBK31 share an mtDNA haplogroup, including a mutation private to Winterborne Kingston (Supplementary Note 2.4; Supplementary Tables 7, 8), implying that they are recently related along the female line. The two share very long IBD1 segments with one another (Supplementary Figure 16), including the entirety of chromosome 21, indicating that they are separated by only a small number of meioses. They do not share any IBD2 segments. Thus, we assume that this 3rd degree relationship is a simple female-line relationship.

Upon further examination, we find the most likely relationship is great-grandmother and son, based on the following observations:

1. The number of IBD segments shared between these individuals falls within the range seen for a maternal-maternal great-grandmother-child relationship and outside the range seen for other female-line relationships (Supplementary Figure 16).

2. WBK12 shares the entirety of chromosome 21 in IBD with both WBK31 and her daughter WBK22. No small gaps were present in this IBD segment. This implies relatively few meioses separate WBK12 and WBK31. Using ped-sim, we find that the probability of observing at least one whole chromosome in IBD is:
    a. 13.3% for a maternal-maternal great-grandmother and child (2,000 simulations)
    b. 4.3% for a maternal-maternal grand-avuncular relationship (4,000 simulations)
    c. 4.7% for a maternal-maternal half-avuncular relationship (4,000 simulations)
    d. 1.7% for maternal parallel cousins (based on 3,000 simulations).

3. WBK12 shares four very large IBD segments (>74 cM) with WBK31, again suggesting a limited number of meioses. This is observed even when the small gap merging threshold is reduced to 0.5 cM. The probability of observing four segments >74cM is:
    a. 8.65% for a maternal-maternal great-grandmother and child (2,000 simulations)
    b. 0.58% for a maternal-maternal grand-avuncular relationship (4,000 simulations)
    c. 0.35% for a maternal-maternal half-avuncular relationship (4,000 simulations)
    d. 0.13% for maternal parallel cousins (3,000 simulations).

As WBK12 is estimated to be a 4th degree relative of WBK31's daughter WBK22, he cannot be a descendant of WBK22 or WBK22's full sibling. Thus, he must be WBK31's great-grandson through another male as opposed to WBK22's father.

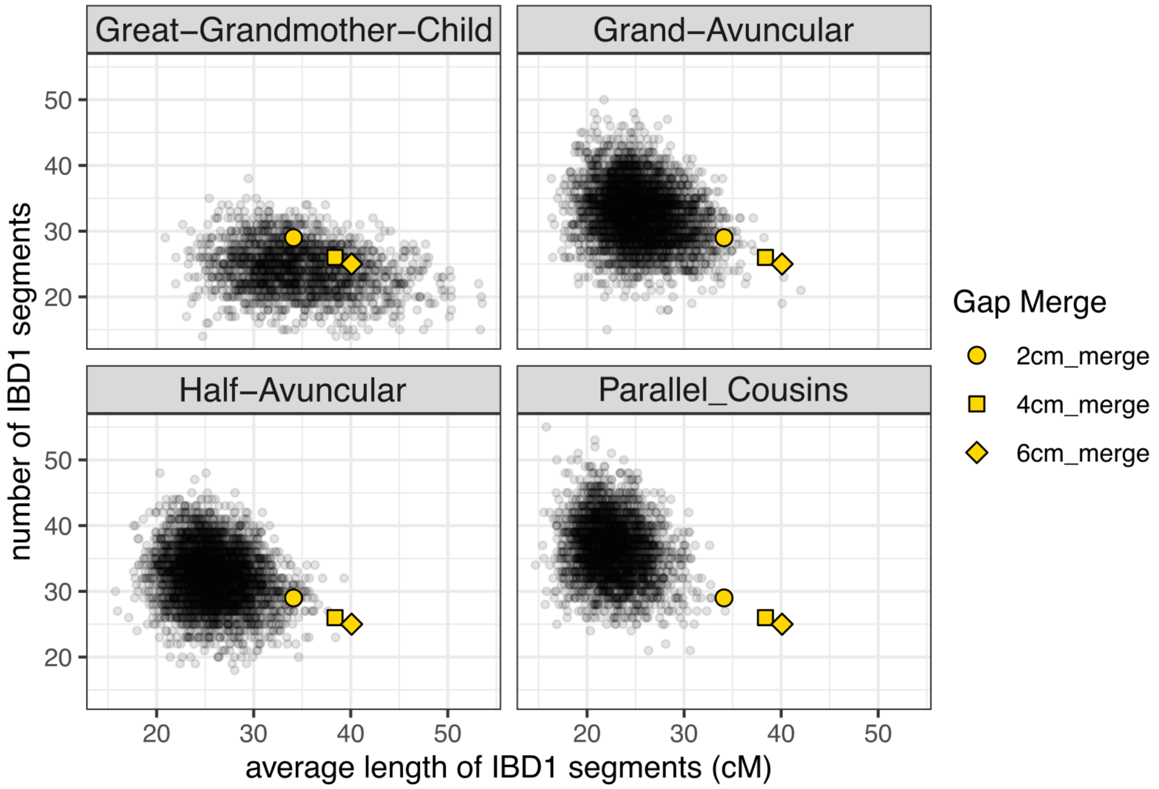

Supplementary Figure 16. Resolving the relationship between WBK12 and WBK31. Simulated ped-sim data is shown in black (1000 replicates for each scenario) for the four possible female-line simple 3rd degree relationships that could exist between these individuals, given their shared mtDNA haplotype with private mutation. The number of autosomal IBD segments is plotted against the average segment length (cM) in each panel. Data points for WBK12 and WBK31 are shown as coloured symbols. We present results for both the <4cM gap removal threshold, used for estimation of r^{β}, as well as the <2cM and <6cM thresholds using different symbols.

WBK02 and WBK20: Maternal-paternal great-grandfather and great-grandson

These two males are 3rd degree relatives and do not share their mtDNA or Y chromosome. They also share no X chromosome IBD segments. WBK02 has one other identified relative on the site, a 2nd degree relative, WBK195, who shares the same rare Y chromosome haplotype (G2a2b2a1a1b1a1a2). No other relatives of WBK02 were detected at Winterborne Kingston (<22cM for all individuals) and we note that the individual is an outlier in PCA and SOURCEFIND analysis, suggesting he or his immediate ancestors derived from continental Europe (Supplementary Note 6).

To further narrow down the relationship between these two individuals, we compared the total length of their genome in IBD and the number of IBD segments they share to simulated data of 3rd degree relationships (Supplementary Figure 17). We did not include any relationships that would lead to a shared mtDNA or Y chromosome haplotype. We find the best fitting relationships are great-grandfather and child or maternal-paternal half-avuncular. The values seen for WBK02 and WBK20 fall outside or on the very edge of simulated distributions for the other relationships considered.

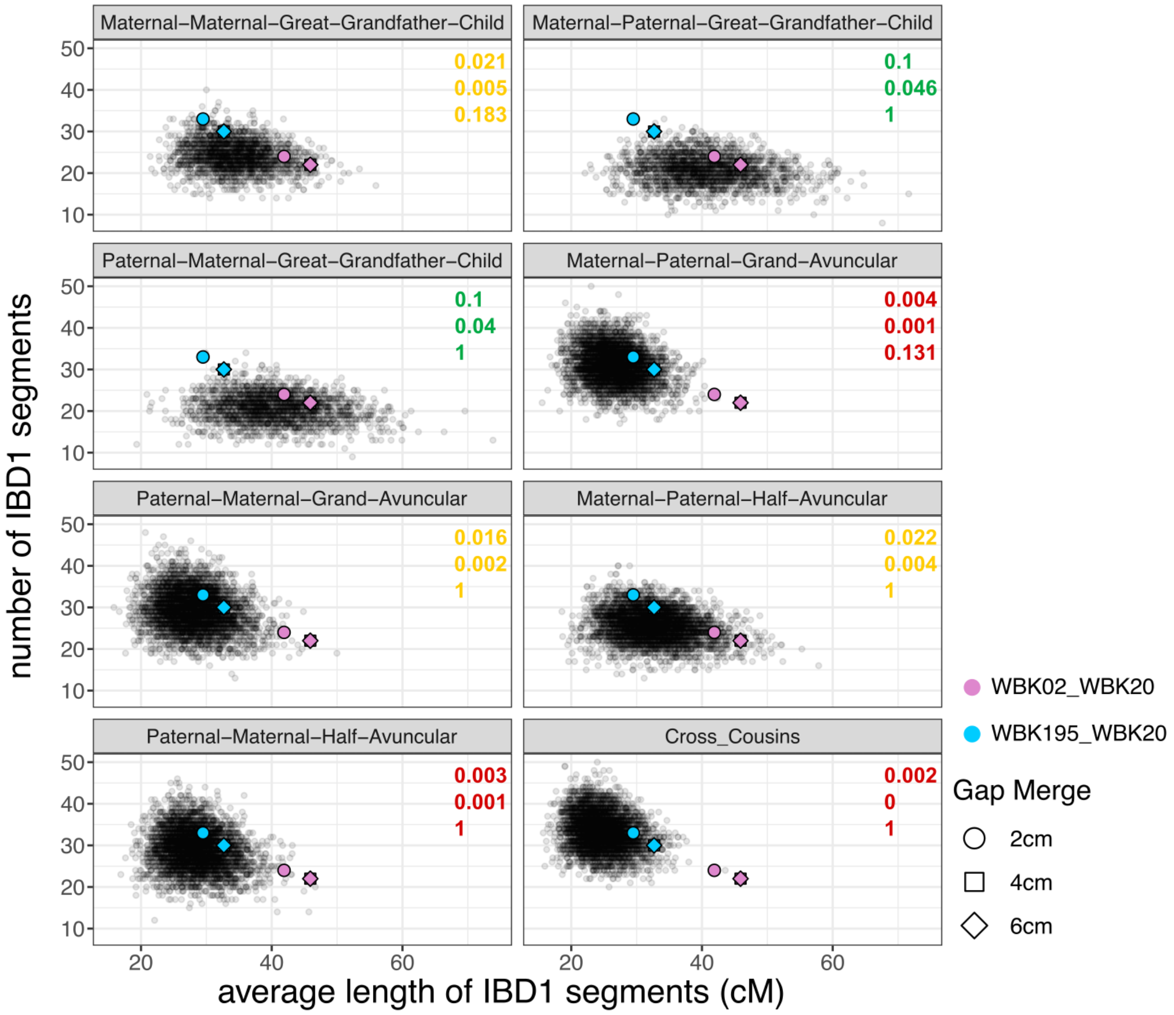

Supplementary Figure 17. Resolving the relationship between WBK20 and his 3rd degree relatives. WBK20 does not share his mitochondrial or Y chromosome haplotype with WBK195 or WBK02. This is compatible with eight possible simple 3rd degree relationships. Simulated ped-sim data is shown in black for each of these (1000 replicates for each scenario). The number of autosomal IBD segments is plotted against the average segment length (cM) in each panel. Data points for WBK02 and his relatives are shown as coloured symbols. We present results for both the <4cM gap removal threshold, used for estimation of r^{β}, as well as the <2cM and <6cM thresholds using different symbols. To further characterise the relationship between WBK02 and WBK20, the probability of sharing (1) two IBD segments >116 cM, (2) three IBD segments >108 cM, (3) no IBD segments on the X chromosome, is shown in the descending order in the right-hand corner of each plot. Green represents the most likely relationships for WBK02 and WBK20, given these probabilities, red the least likely, and yellow intermediate.

We also consider the length of the longest IBD segments shared between WBK02 and WBK20. Two IBD segments over 116 cM in length are observed with no small gaps present within them. Their third longest shared segment is 108 cM in length, with only one small gap (1.58 cM) within it, which was merged for analysis. This implies that relatively few crossover events have occurred on the lineages separating these individuals. We compare these observations to expectations based on simulated 3rd degree relationships (Supplementary Figure 17) and find a paternal-maternal or maternal-paternal great-grandfather and son relationship is most probable. These types of relatives:

1. Have a 10% probability of sharing two IBD segments >116 cM
2. Have a 4-4.6% probability of sharing three IBD segments >108 cM
3. Never share IBD on their X chromosome.

As WBK02 is not related to any of WBK20's relatives (WBK18, WBK06, WBK23, WBK14), we can conclude that WBK02 is not a descendant of WBK20 or his full siblings**.** Thus, he cannot be the great-grandson or grand-nephew of WBK20. We also note that WBK02 and WBK20 both died at a similar age in adulthood (c. 40-45 years), but that WBK02 (195-4 cal BC) pre-dates WBK20 (cal AD 26-207). Thus, WBK02 is very likely to come from an earlier generation.

From this we conclude that WBK02 is mostly likely to be WBK20's:

1. Maternal-paternal grandfather
2. Paternal-maternal grandfather

There are four less plausible relationships, which would require the 108 cM shared between WBK02 and WBK20 to be incorrectly called. These also have a less than 2.2% probability of sharing two segments above 116 cM. In these scenarios, WBK02 would be WBK20's:

1. Maternal-maternal grandfather
2. Paternal-maternal granduncle
3. Maternal-paternal half-uncle
4. Maternal-paternal half-nephew

The exact relationship is further deduced in the following section by fitting these individuals in a pedigree with WBK02's paternal 2nd degree relative, WBK195.

WBK20 and WBK195: Maternal-paternal Half-Uncle and Half-Nephew

WBK195 is a 3rd degree relative of WBK20. WBK02 is a 2nd degree relative of WBK195 and a 3rd degree relative of WBK20. Only certain combinations of 2nd and 3rd degree relationships between

WBK02 and his two relatives will result in WBK195 and WBK20 having a 3rd degree relationship with one another. We constructed pedigrees based on all possible combinations of WBK02+WBK195 and WBK02+WBK20 relationships and for each we calculated the expected degree of relatedness between WBK195 and WBK20 (Supplementary Table 23). Four pedigree structures were found to predict a 3rd degree relationship between WBK195 and WBK20 (Supplementary Figure 18).

Pedigree A: WBK02 is the maternal-paternal grandfather of WBK20, with WBK195 a grandson of WBK02 and a half-uncle of WBK20. This fits with our identification of this relationship as one of the most likely between WBK02 and WBK20 based on IBD segment sharing (see previous section), as well as the earlier radiocarbon date of WBK02.

Pedigrees B, C and D: WBK02 is a paternal half-sibling of WBK20's mother (maternal-paternal half-uncle and nephew), while WBK195 is a 1st degree relative of WBK02's father - (B) father, (C) son or (D) brother. This does not fit with the observed IBD segment sharing. It would also predict only a generation's separation between WBK02 and WBK20, which is incongruent with radiocarbon dates.

We can further rule out pedigrees B and D, as these would require WBK14, a 3rd-5th degree relative of both WBK195 and WBK20 to be related to WBK02. However, this is not observed (<15 cM shared). Pedigree C would require WBK14 to be related to WBK195 and WBK20 through independent paths to avoid relatedness with WBK02. However, WBK14 has a substantial number of IBD segments in common with both WBK195 and WBK20, which implies a shared ancestral path.

Thus, based on all of the available data, we can conclude that WBK02 is the maternal-paternal great-grandfather of WBK20 (pedigree A). In this scenario, WBK195 must be the paternal grandson of WBK02 and the maternal-paternal half-uncle of WBK20 (Fig. 1a).

Relationship to WBK02		WBK20					
		maternal-paternal grandson	maternal-maternal grandson	paternal-maternal grandson	maternal-paternal half-uncle	maternal-paternal half-nephew	paternal-maternal grandnephew
WBK195	paternal grandson (WBK20's half first cousin once removed)	5th	5th	5th	X	X	X
	paternal grandson (WBK20's first cousin once removed)	4th	4th	4th	X	X	X
	paternal grandson (WBK20's half-uncle)	3rd	X	X	X	X	X
	paternal grandson (WBK20's uncle)	2nd (incorrect mtDNA)	X	X	X	X	X
	paternal grandson (other relationship to WBK20)	X	X	X	4th	5th	5th
	paternal grandfather	5th	5th	5th	unrelated	3rd	4th
	paternal half-brother	5th	5th	5th	unrelated	3rd	4th
	paternal uncle	5th	5th	5th	unrelated	3rd	4th
	paternal nephew	5th	5th	5th	4th	4th	4th

Supplementary Table 23. Expected Degrees of Relationship between WBK195 and WBK20 given their relationship to WBK02. Column names show WBK20's relationship to WBK02. Row names show WBK195's relationship to WBK02. The expected degree of relatedness between WBK195 and WBK20 is coloured by fit with the observed degree, with 3rd being the best fit. Entries with a single "X" represent combinations that cannot exist.

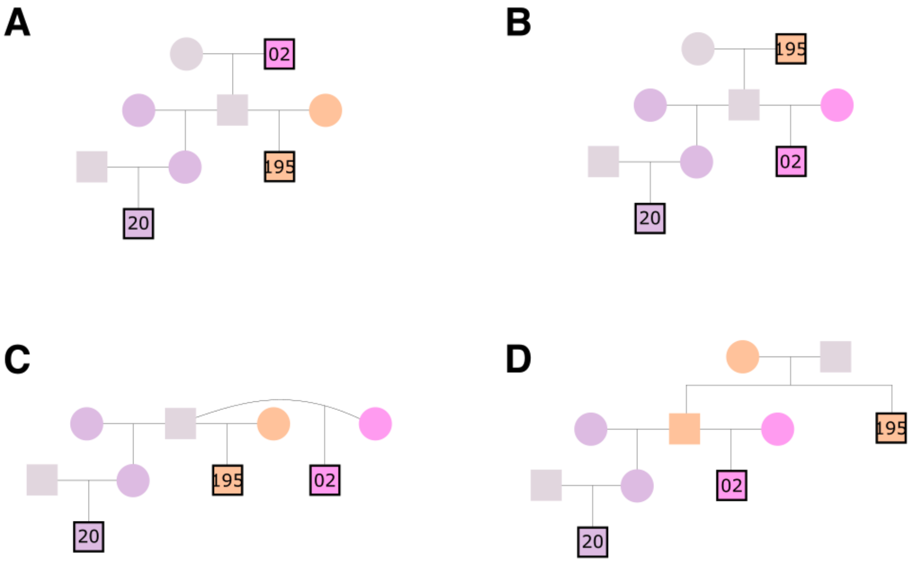

Supplementary Figure 18. Possible pedigree relationships between WBK195, WBK02 and WBK20. Symbol fill colour represents mtDNA haplotype.

WBK06 and [WBK18, WBK23]

WBK06 has two 3rd degree relatives, WBK18 and WBK23. WBK18 and WBK23 are unrelated to each other (only 20cM shared across 7 segments). Therefore, neither can be a descendant of WBK06 or one of his full siblings.

WBK23 does not share his mtDNA or Y chromosome haplotype with WBK06. WBK18 shares his mtDNA haplotype but not his Y chromosome haplotype with WBK06. WBK18 and WBK06 do not belong to the dominant matrilineage of Winterborne Kingston.

One possible scenario is that WBK23 is related to WBK06 through his father and WBK18 through his mother. However, we find that WBK06, WBK18 and WBK23 are all related to WBK20 (4th-5th degree). If this scenario were true, WBK20 would have to be related to WBK18 and WBK23 through either WBK06 or one of his full siblings. This would result in WBK20 sharing a 2-3 degree closer relationship with WBK06, relative to WBK18 and WBK23, which is not observed.

Alternatively, WBK20 could be related to WBK06 through both of WBK06's parents. In this scenario, both WBK06's parents would be 4th degree relatives of WBK20, leading WBK06 to be a "double 5th degree relative". However, given this introduces two additional meioses, we would expect to see an IBD length distribution different to what is typically seen for simple 4th degree relatives. Specifically, we would expect a larger number of shorter fragments. The IBD length distribution of WBK06 and WBK20 falls comfortably within what is observed for other 4th degree relatives within our dataset and far outside the range seen for 5th degree relatives. Thus, we conclude that WBK18 and WBK23 are both related to WBK06 through one of his parents. WBK20 is then related to all of them through a sibling of that parent.

The four possibilities are shown in Supplementary Figure 19. The only scenario compatible with patterns of mtDNA and Y chromosome sharing is WBK18 being related through WBK06's maternal grandmother and WBK23 through WBK06's maternal grandfather. We note that WBK23 died as a juvenile and thus cannot be WBK06's great-grandparent. Thus, there are two possible relationships:

1. Maternal-paternal grand-avuncular (maternal grandfather's sibling)
2. Maternal-paternal half-avuncular (mother's paternal half-sibling)

Based on the total number of shared IBD segments (Supplementary Figure 20), we can eliminate maternal-paternal half-avuncular. Thus, WBK23 is most likely the maternal granduncle of WBK06.

As WBK18 shares an mtDNA haplotype with WBK06 that is rare at Winterborne Kingston, it is likely they are recently related through the female-line and unlikely that WBK18 is WBK06's great-grandfather. Thus, there there are two possible relationships:

1. Maternal-maternal grand-avuncular (maternal grandmother's sibling)
2. Maternal-maternal half-avuncular (mother's maternal half-sibling)

The number of IBD segments shared between this pair is consistent with both relationships (Supplementary Figure 20).

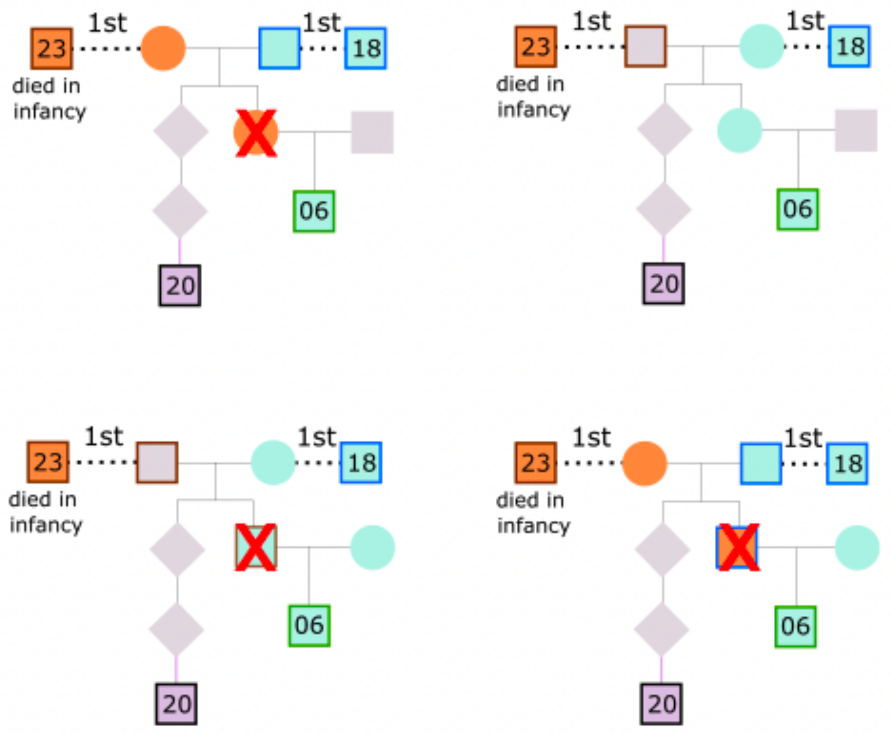

Supplementary Figure 19. Four possible pedigrees for WBK06, WBK18 and WBK23. Symbol fill colour represents mtDNA haplotype and outline colour represents Y chromosome haplotype. In all but one scenario, WBK06's parent has an incorrect Y or mtDNA haplotype, illustrated with a red X.

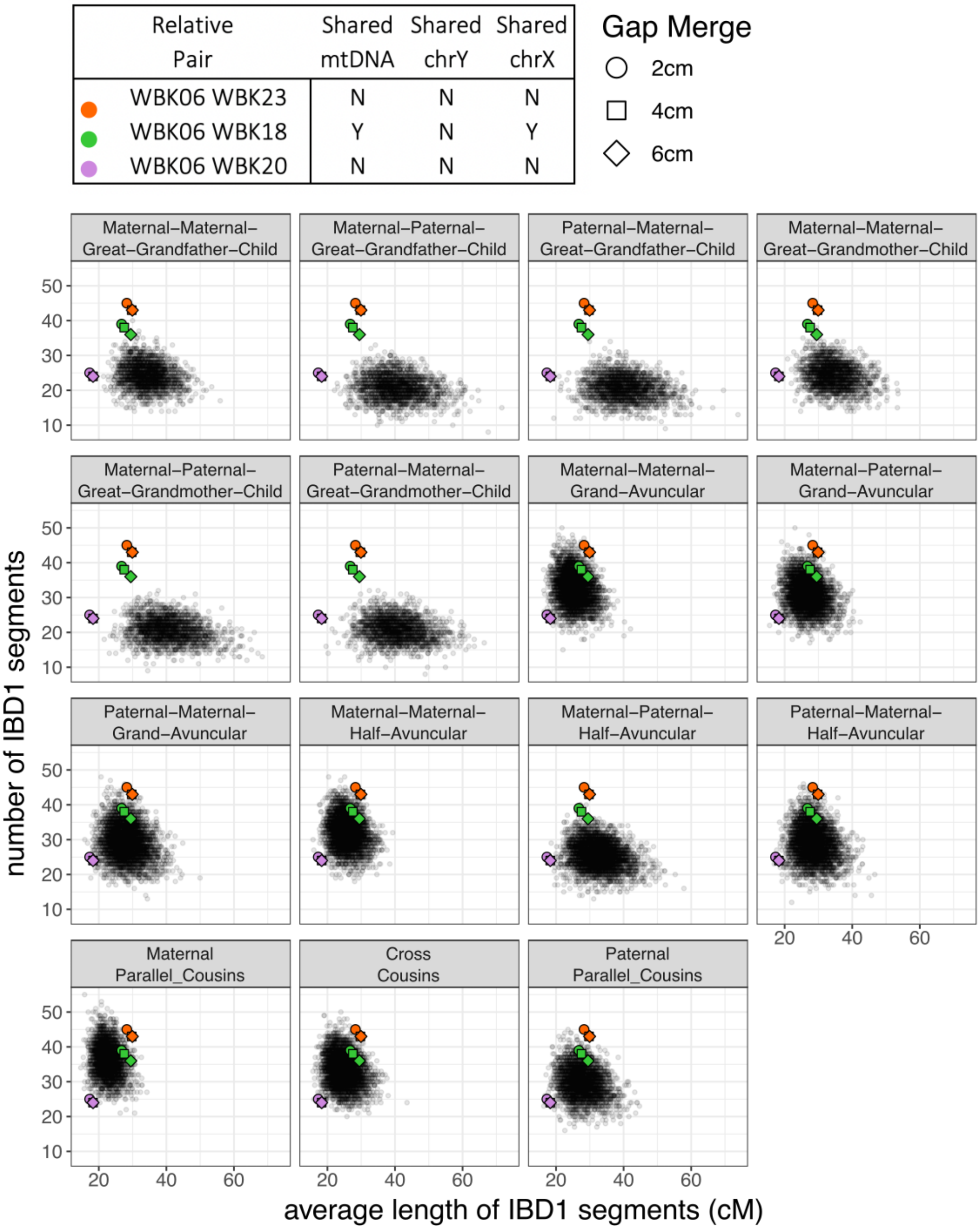

Supplementary Figure 20. Resolving the relationship between WBK06 and his 3rd degree relatives. Simulated ped-sim data is shown in black (1000 replicates for each scenario) for the eleven possible simple 3rd degree relationships that could exist between WBK06 and his 3rd degree relatives WBK23 and WBK18. The relationship between WBK06 and WBK20, which was estimated to be 3rd-4th degree, is also shown. The number of autosomal IBD segments is plotted against the average segment length in each panel. We present results for both the <4cM gap removal threshold, used for estimation of r^{β}, as well as the <2cM and <6cM thresholds using different symbols.

4.10 Resolving 3rd-4th degree relationships

WBK195+WBK20 and WBK14's mother (WBK14-mother)

WBK14 is a relative of the 2nd degree pair WBK195+WBK20, but not WBK02. WBK14's father, WBK17, shows no detectable relationship with WBK195 or WBK20 (<8cM and <44cM respectively). Thus, WBK14 must be related to these individuals through her mother.

WBK14 and WBK195 are 3rd-4th degree relatives, meaning WBK14-mother and WBK195 are 2nd-3rd degree relatives. As she is not related to WBK02, she must be related to WBK195 through his mother or his paternal grandmother. However, if she was related through his mother, she would not show any relatedness to WBK20, which she does (3rd-4th degree relationship). She could be related to WBK20 through a separate path, but, as noted above WBK14 has a number of IBD segments in common with both WBK195 and WBK20, which would require a shared ancestral path. Thus, WBK14-mother must be the paternal-grandmother of WBK195 or a first degree relative of his paternal-grandmother. We choose to show the latter formation on the pedigree in Figure 1.

WBK20 and [WBK06, WBK18, WBK23]

Based on the deductions in the previous sections we can conclude that WBK20 is a first cousin once removed of WBK06 (i.e. WBK20 is the grandson of WBK06's maternal aunt or uncle). This would result in a 4th degree relationship between WBK20 and each of his relatives - WBK23, WBK18 and WBK06, which fits with our observations (Supplementary Figure 9; Supplementary Table 10). We further consider four possible pedigrees (Supplementary Figure 21). WBK20 is the son of WBK06's mother's:

A. sister's daughter
B. sister's son
C. brother's daughter
D. brother's son

We can rule out pedigrees A and D, as WBK20 does not share their mtDNA haplotype with WBK06 or Y chromosome haplotype with WBK23 (Supplementary Figure 21). Pedigree C can also be ruled out, as if WBK06, WBK18 and WBK23 were related to WBK20 through WBK20's mother's father, then they would also be related to WBK02, WBK195 and WBK14, which they are not.

Thus, we can conclude that WBK20 is the son of WBK06's maternal aunts' daughter. Patterns of X chromosome IBD sharing also support scenario B. WBK20 does not share any X chromosome IBD segments with WBK06, WBK18 or WBK23. This observation is more likely if WBK20 is related to these individuals through his father as he would not inherit his father's X chromosome.

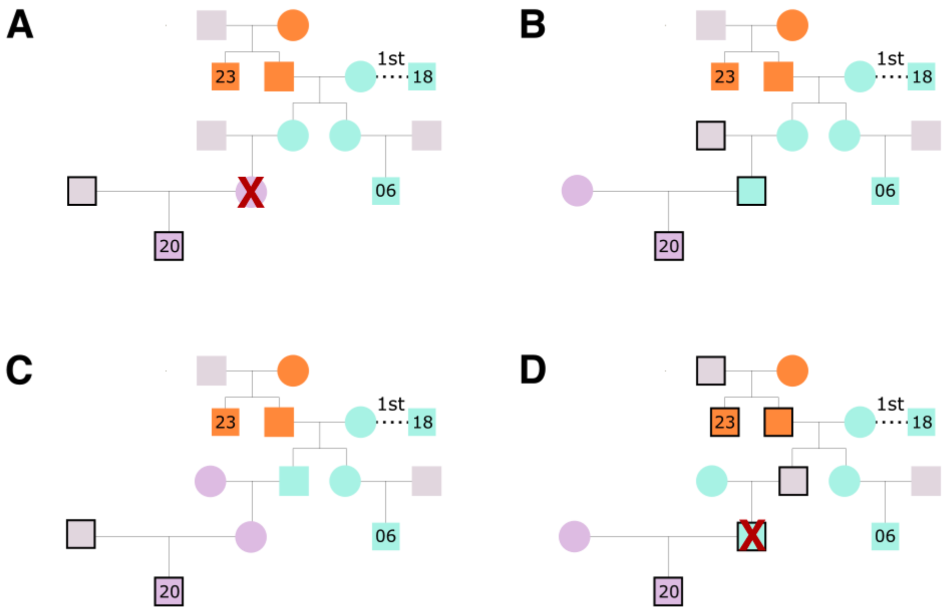

Supplementary Figure 21. Four possible pedigrees for WBK20 and his relatives WBK02, WBK18 and WBK23. Symbol fill colour represents mtDNA haplotype. Black outline represents a shared Y chromosome with WBK20. Scenarios A and D are incompatible with mtDNA and Y chromosome haplotype sharing.

WBK22 and WBK107

WBK22 and WBK107 are identified as 3rd degree on the basis of allele sharing (r^{α}=0.0893). WBK107 shows no relationship to WBK31 (r^{α}=0.0059), the mother of WBK22. Thus, WBK107 must be related to WBK22 through her father.

WBK30 and [WBK105, WBK34, WBK40 and WBK17]

WBK30 is a 3rd-4th degree relative of WBK105, sharing 470 cM of his genome in IBD with this individual. These two males do not share a Y chromosome but do share one IBD segment on the X chromosome. Thus, they cannot be related through their fathers or sons. They also belong to the dominant matrilineage of the site but share no private mutations. Thus, given the frequency of marriage between individuals of the same matrilineage in this community, we cannot assume they are very recently related along the female line.

WBK30 is also estimated to be 4th-5th degree relative to the maternal relatives of WBK105 - WBK34, WBK40, WBK17 (see Supplementary Note 4.8).

WBK30 cannot be an ancestor, great-granduncle, half-granduncle, granduncle, first cousin or half-first cousin to WBK105, as this would lead to a 3rd degree relationship with the sisters (or WBK17 if the alternate configuration of their double relationship is used) or no relationship at all.

WBK30 also cannot be related to WBK105 through WBK105's children or full siblings, as this would lead to 6th-7th degree relationship with WBK34, WBK40 and WBK17. Thus, WBK30 is most likely related to WBK105 through a first degree relative of his mother.

Thus, WBK30 is most likely a first cousin once removed or half-grand-nephew of WBK105.

If WBK30 is a first cousin once removed, we would expect him to also be a 4th degree relative of WBK17 and WBK34+40. In this scenario, all three individuals show below average total IBD sharing with WBK30. Indeed, the r^{β} values of these individuals range from 0.0263-0.045 (r^{β} threshold of 0.0422 for 4th degree relatives). Moreover, WBK14 and WBK42 (both 5th degree relatives in this scenario), also show much lower IBD sharing with WBK30 than expected (Supplementary Table 10).

If WBK30 is a half-grand-nephew of WBK105, we would expect him to be a 5th degree relative of WBK17 and WBK34+40, and a 6th degree relative of WBK14 and WBK42. This fits better with the observed amount of IBD sharing with these samples and we present this as the most likely configuration in Figure 1a.

4.11 Resolving more distant relationships

[WBK33 and WBK39] and [WBK30, WBK105, WBK34, WBK40 and WBK17]

WBK33 and WBK39 are father and son, although the generational order is unclear. WBK39 was too low coverage to include in refinedIBD analysis.

WBK33 is a 4th-5th degree relative of WBK105 and WBK17. He is a 5th-7th degree relative of the sisters WBK34 and WBK40. WBK33 also shares an X chromosome segment with WBK105, meaning they cannot be related through their fathers or sons.

WBK33 cannot be the descendant of a WBK105 or a full sibling, as this would result in too distant a degree of relatedness with WBK17. He also cannot be related to WBK105 through WBK105's maternal grandmother, as this would result in too close a relationship with WBK34 and WBK40.

Thus, he most likely related to WBK105 through a maternal half-sibling of WBK105 or WBK105's full aunt or uncle.

WBK106 and WBK14

These individuals are 4th-5th degree relatives, but WBK106 shows no relationship to WBK14's father (WBK17; 7cM shared). Thus, WBK106 is likely a 3rd-4th degree relative of WBK14's mother.

WBK16 and WBK31

WBK16 shares approximately 203 cM of the genome with WBK31, implying a 4th-5th degree relationship. WBK16 also shares an mtDNA haplogroup, including a private mutation with WBK31 and her descendents, WBK12, WBK22, WBK15, WBK19 (Supplementary Note 2.4), implying that they are all recently related along the female line. However, on the autosomes, WBK16 is more distantly related to the sampled descendants of WBK31 than she is to WBK31 herself.

This rules out WBK16 being related to WBK31 through one of WBK22's siblings or WBK22 herself. It also rules out WBK16 being related to WBK31 through WBK31's offspring with the great-grandfather of WBK12. Thus, WBK16 must be related to WBK31 through WBK31's offspring with another male, a sibling of WBK31 or an ancestor of WBK31.

We further find the most likely relationship between WBK16 and WBK31 is a form of cousinship or half-avuncular, with WBK16 placing within a later generation. We come to this conclusion because:

1. WBK22 yielded an earlier direct radiocarbon date than WBK16, while WBK22's daughter predates WBK16's son (Supplementary Figure 22). Thus, it is probable that WBK16 belonged to a later generation than WBK22.
2. If WBK16 is fitted as a descendent of WBK31 or one of WBK31's siblings, a large generation gap occurs between WBK16 and her male partner, WBK06.

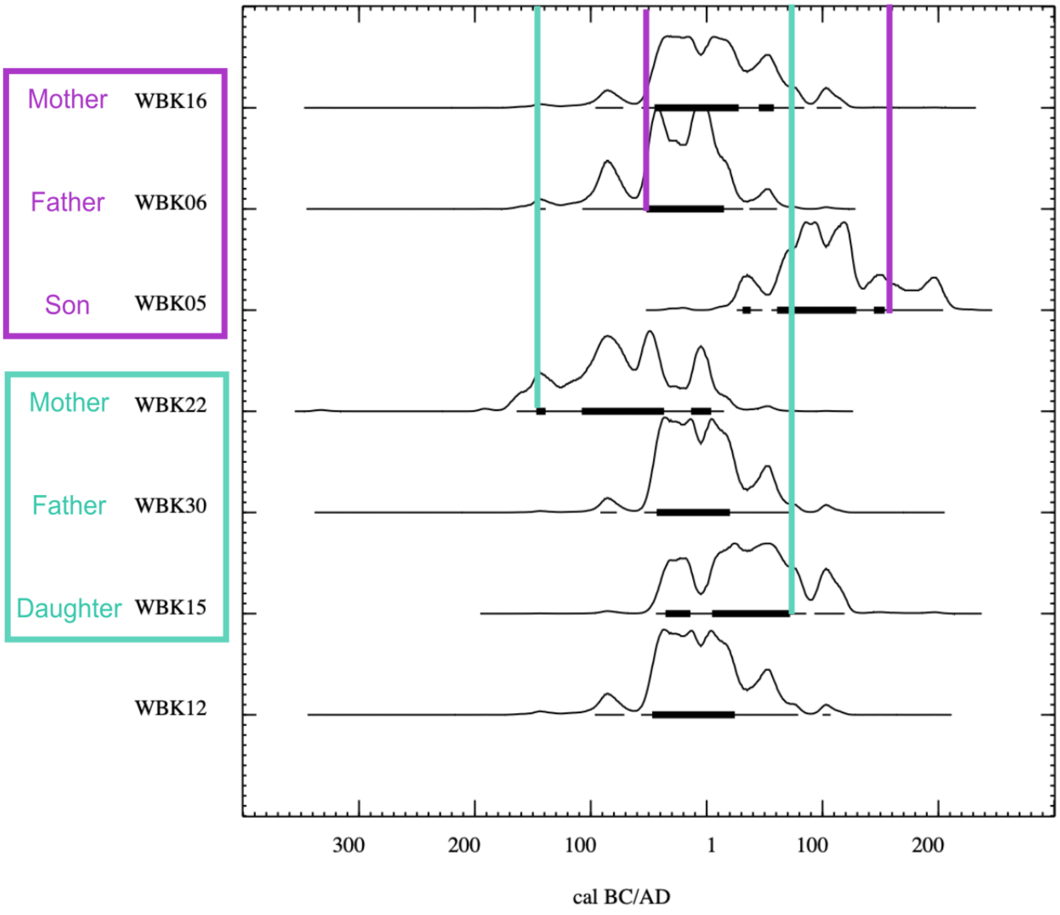

Supplementary Figure 22. Posterior probability distributions of calibrated radiocarbon dates from the immediate families of WBK16 and WBK22. The earliest date observed is from WBK22 and the latest date from WBK05, the son of WBK16.

S5. RefinedIBD Analysis of Iron Age Britain

5.1 Dataset preparation

Both the WGS+SNPCAP1 and WGS+SNPCAP2 datasets were used to characterise patterns of IBD segment sharing across Iron Age Britain. The WGS+SNPCAP1 dataset was used for comparisons between Iron Age sites in northwest Europe, including hierarchical clustering, as well as within site comparisons. The WGS+SNPCAP2 dataset contained additional samples from the Neolithic to medieval era that were not included in the WGS+SNPCAP1 dataset and was used solely for within site comparisons.

Both datasets were generated in an identical manner (Supplementary Note 3). Briefly, each dataset was subject to three rounds of Beagle imputation and phasing. Following this, each Beagle output was filtered for biallelic SNPs with a MAF>0.01 in the dataset and inputted into refinedIBD. We also carried out additional refinedIBD runs where we further restricted the input sites to 1240k positions. This resulted in six sets of IBD segments identified by refinedIBD for both datasets.

Before merging segments across refinedIBD runs, we removed small gaps between IBD segments and updated their LOD scores using the merge-ibd-segments.17jan20.102.jar program (default parameters) available on the software's website. We note that this has the effect of removing information on IBD2 regions. As a conservative measure, these segments were then filtered for a LOD score ≥ 30 and a length ≥ 4 cM. The union of segments across runs was then determined with bedtools[83] and centimorgan positions added using an in-house script.

Homozygous-by-descent (HBD) segments are also outputted from refinedIBD. We filtered these for a LOD score ≥ 10 and a length ≥ 3 cM. Small gaps were then merged and the union of HBD descents was taken for each individual across runs to characterise runs of homozygosity (ROH) within the genome. This dataset was subject to different individual filters based on downstream analysis. This included geographical and temporal filters, as well as a minimum genotype missingness (MIND) across the original 1240k autosomal sites used as input into Beagle.

5.2 Detecting relatives

The accuracy of refinedIBD has been previously assessed by Ramstetter et al. (2017) for relationships up to the 8th degree[85]. This study found the software correctly distinguished >80% of reportedly unrelated pairs from relatives of the 8th degree or closer. The authors noted that the presence of unreported relationships may have confounded this accuracy estimate, which is lower than that reported by refinedIBD's authors[77]. In an effort to reduce false positives, we chose to impose stricter filters (see

above; LOD score ≥ 30, length ≥ 4 cM) than that of Ramstetter et al., who used the default thresholds in their analysis (LOD score ≥ 3, length ≥ 1.5 cM)

Ramstetter et al. used a threshold of at least 19.5cM shared between individuals to define them as 8th degree relatives, based on the lower bound of the expected r^{β} value for 8th degree relatives (r^{β}=0.00275). We use a slightly stricter threshold; instead of using the lower bound of r^{β}, we take the middle value between this lower bound and the average value (r^{β}=0.0039) expected for 8th degree relatives, which corresponds to 24 cM shared for a haploid genome length of 3545.83 cM. We further required pairs to share at least three IBD segments to be classified as related. While we expect these stricter thresholds to increase our false negative rate, we can be more confident in the distant relatives we do identify.

5.3 Patterns of IBD sharing within archaeological sites

To characterise patterns of IBD sharing within sites, we filtered the combined WGS+SNPCAP1 and WGS+SNPCAP2 datasets for a MIND of 0.4 and removed archaeological sites with only one individual remaining. We then calculated the following values:

1. The total length of IBD shared between individuals (*cm_raw*) and the average total length shared between pairs (*cm_norm*).
2. The total number of IBD segments shared between individuals (*seg_raw*) the average number of segments shared between pairs (*seg_norm*).
3. The total length of ROH summed across all individuals (roh_cm) and the average length of ROH within each individual (*roh_cm_norm*).
4. The total number of runs of homozygosity (ROH) summed across all individuals (roh_seg) and the average number of ROH within each individual (*roh_seg_norm*).
5. The number of related pairs identified within the site (rels). This value was divided by the number of pairwise comparisons made to calculate a *rel_norm* value. If the *rel_norm* value is one, this implies all pairs of individuals in a site are genetic relatives, while if *rel_norm* is zero, this implies that no pair of individuals are genetic relatives.

The results are presented in Supplementary Table 13. The *rel_norm* value (normalised number of relative pairs) was plotted against mtDNA diversity (*h*-value) in Figure 2.

5.4 Population and community sizes in the British Iron Age

The amount of shared IBD within individual genomes (i.e. runs of homozygosity) and between members of a population can give an indication of population size. Small populations will show higher levels of IBD sharing. To examine this, we plotted the *cm_norm* and *roh_norm* values (Supplementary

Table 13) for Iron Age, Roman and post-Roman sites across Britain, France and the Netherlands in Extended Data Figure 6. We note that very few British individuals dating between 150-500 AD exist in the dataset. These come from Scotland, WBK and Driffield Terrace in Yorkshire.

Levels of IBD sharing within sites are not only influenced by population size, but also social organisation. To remove the confounding impact of close biological relatedness, we calculate the levels of IBD sharing seen within different British regions, excluding within site comparisons. Specifically, we calculated *cm_norm* values between sites within a region. This is defined as the total amount of IBD shared between two sites, normalised by the number of pairwise comparisons made. We then took the average value of *cm_norm* between sites for each geographical region. We chose to use present-day county divisions to define regions (see Supplementary Table 12). We excluded the Roman burial site of Driffield Terrace in Yorkshire, given the large number of genetic outliers present at the site (Supplementary Note 6.3). For comparison we also calculate this metric for the Paris Basin, Rhine region and southern coast of France.

We find that lowest values for within genome, site and region IBD sharing are concentrated in the southeast, implying larger, denser populations in this region. Lowest values are seen in peripheral regions, particularly Cornwall and Yorkshire.

5.5 Incidences of inbreeding at Winterborne Kingston

We calculated the amount of the genome in homozygous segments > 3cM for 43 individuals from Winterborne Kingston. We note three additional individuals were available for this analysis, who were not included in Supplementary Note 3, due to more relaxed coverage thresholds. Of these 43 individuals only one, WBK36, shows evidence of recent inbreeding (Supplementary Figure 23). This individual dates to the Roman period of the site's usage and is buried in a new custom (extended inhumation). Six other individuals, including four from the main WBK kin-group, have levels of ROH that would be expected for the offspring of 5th to 7th degree relatives (e.g. 2nd to 3rd cousins). The remaining 35 individuals have parents that can be classified as unrelated (> 7th degree). This suggests that consanguineous marriages were not favoured within the community. The results are in agreement with the lack of relatedness observed between the two parental pairs in our dataset (WBK22+WBK30 and WBK06+WBK16).

We further validated our findings with hapROH [92], a pipeline tailored towards pseudo-haploid data. We called pseudo-haploid genotypes for the 1240k SNP capture panel[93] in northwestern Iron Age individuals, retaining those with base calls for more than 400,00 sites. The software was run with default settings, with highly concordant results obtained (Supplementary Figure 24). We also compared *roh_norm* values produced with refinedIBD and hapROH for northwestern Iron Age sites, with high concordance again obtained (Supplementary Figure 25).

We used hapROH to plot individual ROH profiles for all northwestern Iron Age samples in the dataset (Supplementary Figure 26). Several pairs, including WBK36, display profiles consistent with parents who are 3rd degree relatives (e.g. first cousins). However, the majority of samples have parents who are not detectably related, suggesting that consanguineous marriages were not a common custom in Iron Age Britain. One site that is a potential exception is Tregunnel (Cornwall) where two of the three individuals analysed have 142-324 cM of their genomes in ROH. The third individual from Tregunnel only has one 4cM segment. Our results are in accordance with prior work on British ancient genomes, which estimated that mating pools increased roughly fourfold from the Neolithic to Iron Age [34].

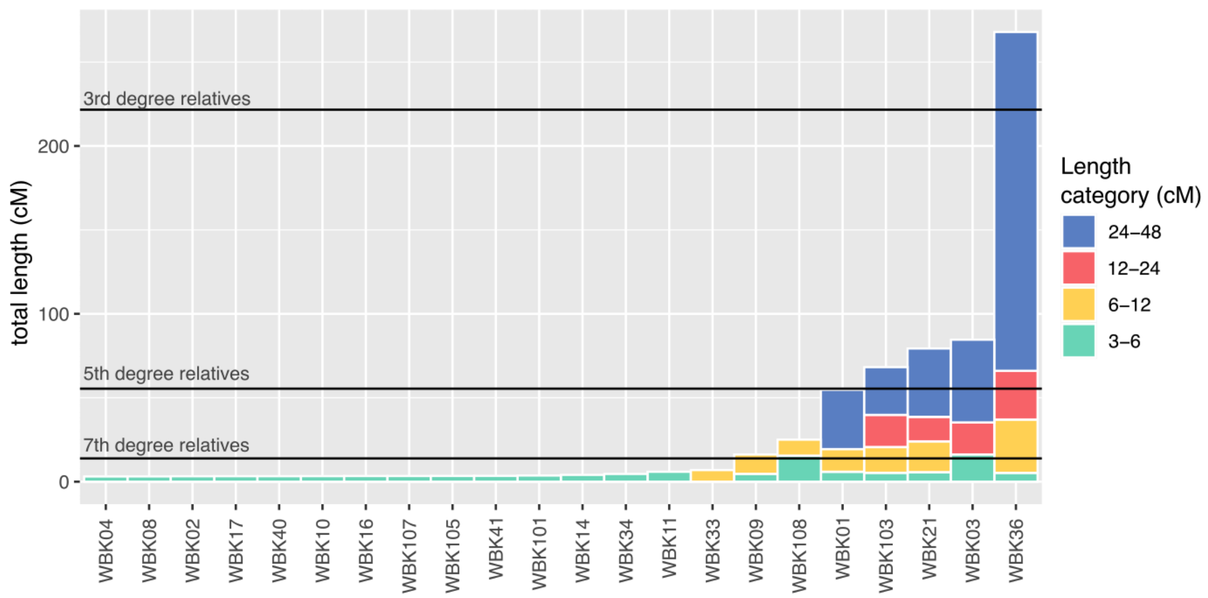

Supplementary Figure 23. Total length of runs of homozygosity for WBK genomes estimated by refinedIBD. ROH lengths are totalled for different segment length categories. Black horizontal lines show the expected values for the offspring of 3rd, 5th and 7th degree relatives. An additional 21 analysed individuals were excluded from this plot due to no ROH segments being identified.

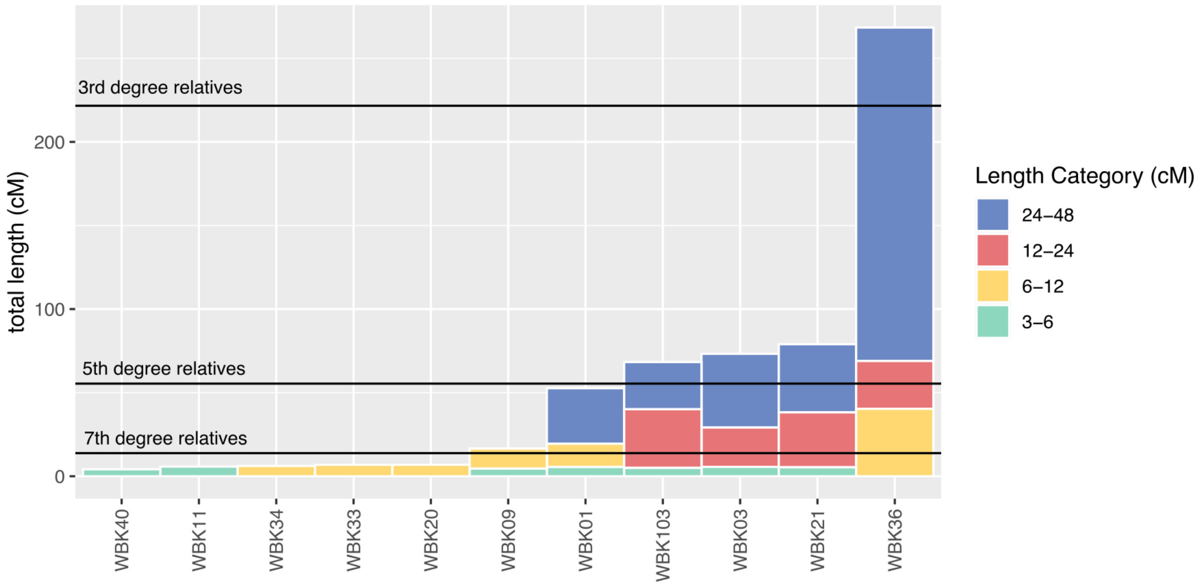

Supplementary Figure 24. Total length of runs of homozygosity for WBK genomes estimated by hapROH. ROH lengths are totalled for different segment length categories. Black horizontal lines show the expected values for the offspring of 3rd, 5th and 7th degree relatives. An additional 21 analysed individuals were excluded from this plot due to no ROH segments being identified.

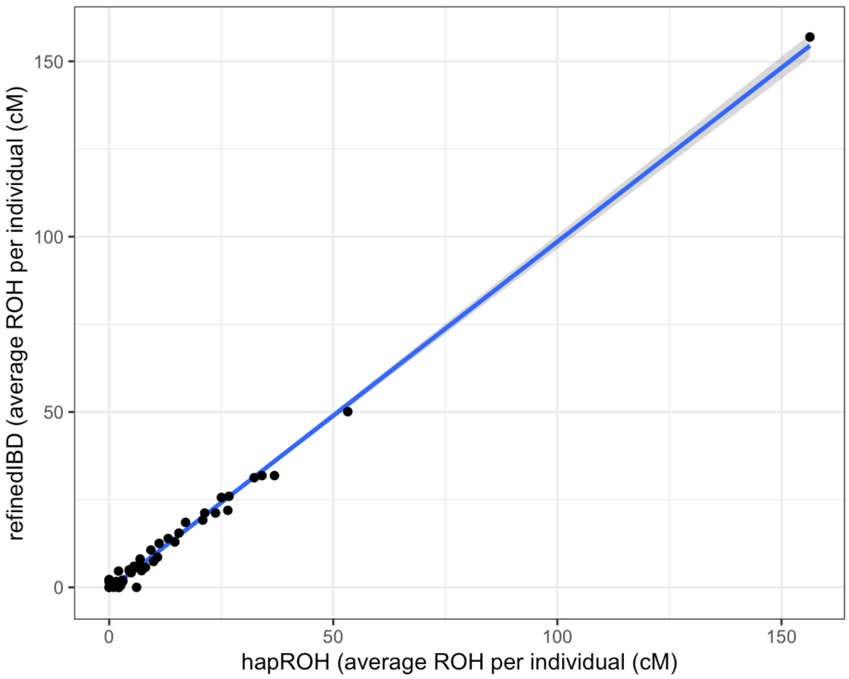

Supplementary Figure 25. Comparison of refinedIBD and hapROH pipelines for detecting ROH in northwestern Iron Age individuals. Data points are archaeological sites. A strong correlation is seen for the amount of the genome in ROH (cM) per individual estimated using the different pipelines.

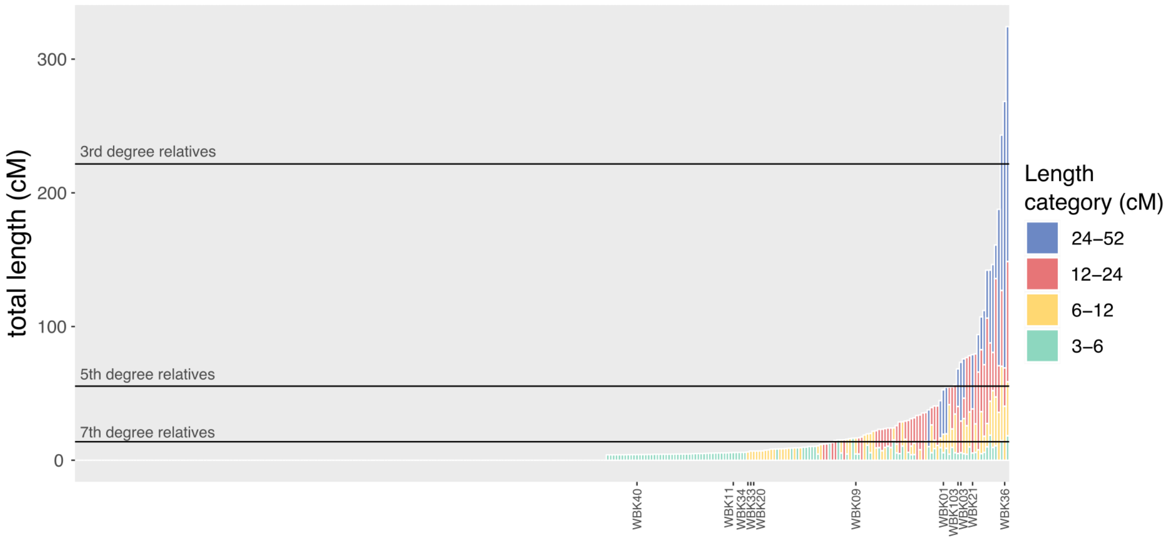

Supplementary Figure 26. Total length of runs of homozygosity for northwestern Iron Age genomes estimated by hapROH. ROH lengths are totalled for different segment length categories. Black horizontal lines show the expected values for the offspring of 3rd, 5th and 7th degree relatives. Samples from the current study with a total length of ROH > 0 are labelled.

5.6 Relatives within and between British Iron Age sites

We extracted all British Iron Age, Roman and post-Roman individuals with a MIND<0.4 (n=303) from the WGS+SNPCAP1 relax filter dataset and identified 11,221 IBD segments shared among them. We found 6,373 of these were shared within sites and 4,848 of these between sites. In total, we identified 386 pairs of close and distant relatives in our British Iron Age dataset (Supplementary Table 14). Of these, only 30 pairs were observed between sites. These pairs shared between 24-70 cM, which reflects approximately 7th-8th degree relationships, all though we note there is a high overlap of relatedness coefficients among higher degrees of distance.

We further found that 50.8% of relative pairs within sites shared an mtDNA haplogroup, with no mtDNA sharing seen among relative pairs between sites. If we filter our relatives within sites to remove those who share more than 70 cM, we find that 39.1% of individuals share an mtDNA.

The majority of between site relative pairs were mixed sex (n=16), followed by male-male (n=9) and female-female (n=5). We note an opposite skew towards female-female pairs is present in our set of relative pairs found within sites - mixed pairs (n=187), female-female (n=98), male-male (n=71). However, this skew is not significant (chi-square test). We also compared the proportion of distant relative pairs (24-70 cM shared) who shared an X chromosome IBD segment within and between sites. While a lower proportion of relatives between sites shared IBD on the X chromosome (6.67%) this was not significantly different to within site pairs (16.67%).

Relatives between sites

The majority of relative pairs identified between sites linked communities in close proximity to one another. We provide a brief description of each pair below.

Neighbouring Sites:

1. **Dibbles Farm and Worlebury, North Somerset**: Eight between-site relative pairs were observed, derived from all five individuals from Worlebury and four of the eight individuals from Dibbles Farm. Worlebury Camp is a large multivallate hillfort situated on a coastal promontory with views of the Bristol Channel. Dibble's Farm is found further inland, approximately 7.3 km southeast of Worlebury, and is a farmstead with a series of associated burials. The human remains from Worlebury are of unknown context: bone was recovered from storage pits as well as deposits relating to a potential massacre. All samples included in IBD analysis have been directly dated and are broadly contemporaneous, spanning the Middle Iron Age.

2. **Nunburnholme Wold and Pocklington, East Yorkshire:** Nunburnholme Wold is a hilltop which contains the remains of an Iron Age cemetery and associated enclosures and drove ways. These surround an ovoid open area, interpreted as a meeting point that would have served the surrounding region. Pocklington is a cemetery associated with the Arras Culture that is situated in a valley approximately 4 km west of Nunburnholme Wold. The two individuals analysed from Nunburnholme Wold have multiple relatives at Pocklington. I5502 is a relative of I11033+I14105, while I5503 is a relative of I12411+I12413+I13756+I14104+I14105+I5505+I5506. Notably, the Nunburnholme Wold individuals (I5502 and I5503) are unrelated to one another, only sharing one IBD segment (13 cM). The Nunburnholme individuals both have associated radiocarbon dates, spanning 363-3 cal BC, while the Pocklington individuals date to 400-50 BCE.
3. **Pocklington and East Coast Pipeline Field 16, East Yorkshire:** Field 16 of the East Coast Pipeline is located approximately 20 km east of Pocklington, between Boynton and Argham. It comprises a multi-phased enclosure complex dating to the Iron Age and Roman period. Two individuals, I22064 and I14327, associated with a square barrow to the north of the enclosure, were found to have relatives at Pocklington: I22064 (105 cal BC –cal AD 64) is a relative of I5505 and I5507, while I14327 (340–47 cal BC) is a relative of I14102.
4. **Tregunnel and Harlyn Bay, Cornwall:** Harlyn Bay is an Iron Age cist cemetery located approximately 16 km north of Tregunnel, another site with Iron Age burial activity. Both sites are in close proximity to the coast. The individual I12772 from Harlyn Bay has two relatives at Tregunnel - I12792 and I12793, who are a mother and son pair. Notably, I12772 shared 17cM across 2 IBD segments with a 2nd-3rd degree relative of the mother and son, I12790. No direct radiocarbon dates are available for the analysed samples, but the Tregunnel remains are contextually dated to 400-100 BC, while I12772 may date anywhere between 800 BC and AD 43. However, given I12772's relationship with the Tregunnel samples, it is likely that this sample is approximately contemporaneous in date.
5. **Cliff's End Farm and East Kent Access Road, Kent:** Cliff's End Farm witnessed funerary activity throughout the Late Bronze Age and Iron Age. It comprises two enclosures, possibly used for communal meetings, and a mortuary feature containing both articulated and disarticulated human bone. One individual sampled from this feature, I14380 (387–203 cal BC), has a relative**,** I13615, buried 2 km away at the Middle Iron Age cemetery at East Kent Access Road. I13615 is contextually dated to 400-200 cal BC. Notably, I14380 had an isotopic signature that suggested he was a non-local. Interestingly, very little relatedness was found among the populations of both sites, despite relatively large sample sizes. Only one pair of relatives, a mother and daughter, have been identified at East Kent Access Road. Of the remaining nine pairwise comparisons that can be made within both sites, the maximum amount of IBD shared was 8cM.

6. **Danebury, New Buildings and Suddern Farm, Hampshire:** Danebury is a hillfort that has yielded approximately 300 deposits of human remains, mostly found in storage pits. A male individual from Danebury (I17264; DA75; Deposit 28; Pit 829) contextually dated to 450-100 BC, was found to have two relatives in neighbouring sites. One relative, I20984, was identified 5 km away at Suddern Farm, a site which includes an Early-Middle Iron Age cemetery contemporaneous with Danebury. I20984 is contextually dated to 450-1 BCE. The other relative, I17260, was identified at New Buildings. This sample, a cranial fragment of a child (NB92), is not dated, but was found in the upper fill of Early Iron Age pit P232(1) Tr 2. The assemblages recovered from the Early Iron Age pits at New Buildings are comparable to those at Danebury, 3 km to the west.
7. **Ham Hill and Winterborne Kingston, Dorset:** The male child WBK43 (156 cal BC – cal AD 20; *circa* 1-3 years) has a male relative at Ham Hill hillfort - I19855. This sample derives from the hill's north 'spur' quarry works and is contextually dated to 400-100 cal BC. We note that WBK43 does not belong to the dominant matrilineage at Winterborne Kingston and has no relatives identified at the site. Ham Hill lies approximately 40 km to the northwest of Winterborne Kingston and as Britain's largest known hillfort, would have been an imposing presence within the region.

Distant Sites:

8. **Winterborne Kingston, Dorset, and Carsington Pasture Cave, Derbyshire:** One individual from Winterborne Kingston, WBK01 (362-169 cal BC), has two relatives at Carsington Pasture Cave in the southern Peak District (Derbyshire). Carsington Pasture Cave has produced evidence of burial activity from the Early Neolithic to the Iron Age. The two relatives of WBK01 are contemporaneous. I12771 is dated to 513-210 cal BC and I3014 to 377-176 cal BC. Both samples derive from disarticulated adult temporal bones from various contexts within the cave. WBK01 shared 31 cM across three segments with I12771 and 39 cM across three segments with I3014.

   Given that all other pairs of relatives between sites have been found within approximately 40 km of one another, with the exception of the coastal Scottish samples below, this result suggests that WBK01 derives some of her ancestry from the Derbyshire region. To investigate further, we examined the top individual hits for WBK01 across the entire British Iron Age dataset (Supplementary Table 24) and found that 5/7 individuals she shares >7cM with are from Derbyshire, while only one is from southern England. In a more extensive analysis of IBD sharing at Winterborne Kingston (Supplementary Note 4), we find WBK01 has no detectable relatives at the site and does not belong to the dominant matrilineage.

In contrast, while the two Carsington Pasture individuals (I3014 and I12771) are not detectably related, they share 25 cM across 2 large IBD segments, which is just below the required threshold to be classified as 8th degree relatives. We further note that the next top hit for I3014 is a sample (I20621) from the nearby site of Fin Cop, Derbyshire, with whom she shares 16 cM across 2 IBD segments. This suggests that I3014 and I20621 are derived from the local Derbyshire population. Taken together, these data strongly imply that WBK01 or her recent ancestors originated in Derbyshire or neighbouring regions and were non-local to Dorset.

9. **Applecross, Scottish Highlands, the Knowe of Skea, Orkney and Bu, Orkney:** A male individual, I3568 (42 cal BC - cal AD 119), from a multiple grave at Applecross is related to individual KD043 (cal AD 25-214) from an Iron Age cemetery at the Knowe of Skea on Westray. I3568 is also a relative of another earlier individual from Orkney - I2983 (398-207 cal BC) from an Atlantic roundhouse excavation at Bu. The other two individuals analysed from Applecross, I3566 and I3567, share 20 cM across four segments with one another, but only one 5 cM segment total with I3568. Taken together, this data may suggest that I3568 is not local to Applecross and may ultimately originate from Orkney. We note that all of the above sites are coastal, with the Applecross grave constructed from a low mound of beach cobbles. This implies maritime connectivity between the sites.

**Site**	**Sample**	**Segments**	**Total cM**	**Average Segment Size**
Fin Cop, Derbyshire	I20631	2	9.82	4.91
New_Buildings, Hampshire	I17260	3	21.1693	7.05643
Carsington Pasture Cave, Derbyshire	I12771	3	31.1816	10.39386667
Pocklington, Yorkshire	I13760	1	10.5327	10.5327
Carsington Pasture Cave, Derbyshire	I3014	3	38.8221	12.9407
Fin Cop	I20622	1	13.2395	13.2395
Carsington Pasture Cave, Derbyshire	I12775	1	18.995	18.995

Supplementary Table 24. Top IBD hits for WBK01. Five of the seven top hits (total cM > 7cM) come from Derbyshire.

Relatives within sites

Of the 45 British Iron Age sites considered, only 16 had a *rel_norm* value above zero (Extended Data Figure 4, Supplementary Table 13). We observed a reduction in mitochondrial diversity with increasing value of *rel_norm* for these sites (Fig. 2). Each site, with the exception of Winterborne Kingston (Supplementary Note 4), is discussed in turn below, with results presented in Supplementary Table 14. The samples discussed are all derived from Patterson et al. 2022.

1. **Bottle Knap, Dorset:** Two individuals at Bottle Knap, I27381 and I27383, are relatives, sharing 66 cM across 8 IBD segments, as well as a shared mtDNA haplotype (U4c1). No relatives had been previously identified at this site using allele matching methods.
2. **Casterly Camp, Wiltshire:** A pair of 2nd to 3rd degree relatives have been previously identified at Casterly Camp (I21313+I21314) and were re-identified here. This pair do not share an mtDNA haplogroup
3. **Dibbles Farm and Worlebury, North Somerset:** In addition to the eight pairs of relatives identified between these sites, we identified seven pairs of relatives within Worlebury and six within Dibbles Farm. No relatives had been previously identified at these sites using allele matching methods. Together with the low levels of mitochondrial diversity observed at both sites, we can conclude that burial at both Dibbles Farm and Worlebury was at least in part guided by kinship.
4. **East Coast Pipeline Field 16, East Yorkshire:** We identified a pair of previously unidentified distant relatives at this site, I22064+I22052. These individuals do not share an mtDNA haplogroup. No other relatives were detected at this site, although two individuals have relatives at the nearby site of Pocklington.
5. **East Kent Access Road, Kent:** We identified a previously reported mother-daughter relationship at this site. However, we estimated a deflated relatedness coefficient of 0.25 between these individuals, likely a result of inferior IBD retrieval among SNP capture samples.
6. **Fin Cop, Derbyshire:** Two pairs of 2nd to 3rd degree relatives were previously identified at Fin Cop (I20631+I20632; I20623+I20627), all belonging to mtDNA haplogroup V2b. We identify a more distant relative of I20631 (I20622), who does not belong to the V2b haplogroup.
7. **Gravelly Guy, Oxfordshire:** Three pairs of 2nd degree relatives were previously identified at Gravelly Guy. They comprise three individuals, I21276, I21277 and I20584. All three individuals belong to the same mtDNA haplotype - K1a4a1. We identify a further 5 pairs of relatives, comprising the above three individuals and an additional three - I20583, I21274 and I21275. These individuals also belong to haplogroup K1a4a1. The remaining three individuals at the site have no detectable relatives and do not belong to haplogroup K1a4a1.

8. **Harlyn Bay, Cornwall:** We identified one pair of relatives at this site, I12772+I16439, both of whom belong to the same mtDNA haplotype, T2c1d+152. No relatives had been previously identified at this site using allele matching methods.
9. **Law Road, East Lothian, Scotland:** We identified one pair of relatives at this site, I16495+I16499, who do not share an mtDNA haplotype. No relatives had been previously identified at this site using allele matching methods.
10. **Pocklington, East Yorkshire:** Six pairs of relatives had been previously identified at Pocklington. We identified an additional 207 pairs of relatives. This site also exhibits low levels of mtDNA diversity with three major haplotypes identified: K1c1a, J1c9 and H2a3b. Of the 35 individuals at the site, 28 belong to one of these haplotypes. Together with the abundance of genetic relatives, we can conclude that a matrilineal system guided burial at this cemetery.
11. **Rowbarrow, Wiltshire:** We identified one pair of relatives at this site, I19862+I19868, who do not share an mtDNA haplotype. No relatives had been previously identified at this site using allele matching methods.
12. **Tregunnel, Cornwall:** We identified a previously reported mother-son relationship at this site. However, a deflated relatedness coefficient of 0.2 was observed between these individuals, likely a result of inferior IBD retrieval among SNP capture samples. We also identified a previously reported 2nd to 3rd degree relative of the mother-son pair who also shares an mtDNA haplogroup with them.
13. **Trethellan Farm, Cornwall:** We identified one pair of relatives at this site, I16450+I16457, both of whom belong to the same mtDNA haplotype, T1a1. No relatives had been previously identified at this site using allele matching methods.
14. **Wattle Syke Dalton Parlours, West Yorkshire:** We identified one pair of previously reported relatives at this site, I14351+I14352, who are sisters. However, we estimated a deflated relatedness coefficient of 0.11 between these individuals. This is due to the collapsing of IBD1 and IBD2 segments and poorer IBD retrieval among SNP capture samples.

5.7 IBD sharing between Iron Age communities in northwest Europe

In the above section we considered IBD sharing between individuals. In this section, we use the WGS+SNCAP1 dataset to consider trends in IBD sharing between archaeological sites. These are assessed using normalised pairwise metrics. Specifically, we calculate the total number and length of IBD segments (cM) shared between all pairs of individuals between sites and normalise these values by the number of pairwise comparisons made (*cm_norm* and *seg_norm*).

We focus on 298 British individuals (MIND < 0.4) from the Iron Age to post-Roman period (pre-AD 500), alongside 37 individuals from northern France and the Netherlands in these analyses (Supplementary Table 12). We note that only 13 British individuals have point date estimates later than AD 150. These include seven individuals from a Roman cemetery at Driffield Terrace[47], four individuals from Winterborne Kingston, and two individuals from Orkney[46].

IBD neighbourhoods and enclaves

To examine connectivity between Winterborne Kingston and other Iron Age communities, we plot pairwise *cm_norm* values in Supplementary Figure 27. Highest amounts of IBD sharing are seen for other sites in Dorset and surrounding regions. A particular abundance of long shared segments is seen with two Dorset sites approximately 25-30 km west of Winterborne Kingston - Maiden Newton and Bottle Knap. Articulated inhumation burials were discovered at both these sites. Maiden Newton is notable in that it is a well-furnished Durotrigian burial of an adult female. We observe very little IBD sharing between another Durotrigian-type burial at Worth Matravers and the Winterborne Kingston population. In fact, this Worth Matravers sample showed minimal IBD sharing across the dataset - 12 segments total, the longest (8cM) shared with Bottle Knap.

We contrasted the geographical patterns of IBD sharing seen for Winterborne Kingston with those of the second largest population in our dataset, the burial community of Pocklington in East Yorkshire (Supplementary Figure 17). We found that Pocklington shares extremely high *cm_norm* values with neighbouring sites in East Yorkshire. To investigate further, we examined IBD sharing between all northern English sites (between a latitude of 53° and 55°; Extended Data Figure 5). We found remarkably high levels of IBD sharing between all archaeological sites east of the river Derwent, with respect to both overall cM shared and the average length of shared segments. Sites west of the river show no such inflation. It is worth noting that East Yorkshire is associated with a distinctive material culture in the Iron Age, the Arras Culture, with putative ties to the continent. The territory is ascribed to the Parisi Tribe by Ptolemy, bordered by the Brigantes to the north and west, and the Coritani south of the River Humber.

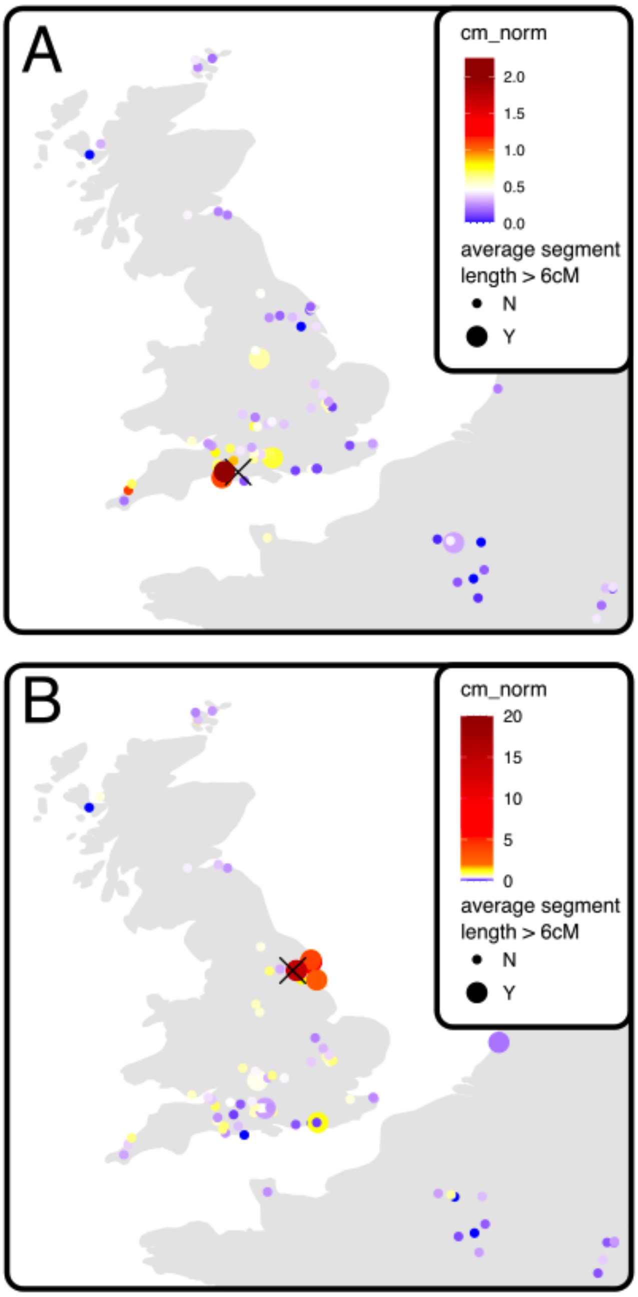

Supplementary Figure 27. IBD Neighbourhoods of large Iron Age cemeteries in Dorset and Yorkshire. A) Shows normalised amounts of IBD sharing (cM) between Winterborne Kingston. Large circles indicate long segments shared on average (>6cM). Inflated values are seen in the regions surrounding Winterborne Kingston, with top hits for the neighbouring Dorset sites of Maiden Newton and Bottle Knap. The inflated sharing with one midlands site (Carsington Pasture Cave) is driven by one sample, WBK01, having relatives at this site. B) The IBD neighbourhood of Pocklington. We note the colour scale here is an order of magnitude higher due to excessive sharing with other sites in East Yorkshire.

Hierarchical clustering of British Iron Age sites using the Leiden algorithm

Individuals were then further filtered for a minimum genotype missingness (MIND) below 0.25 and *cm_norm* values were calculated between archaeological sites. These values were used to construct a distance matrix, which summarised the expected amount of IBD shared between two individuals from any pair of sites. We then constructed a weighted network graph from the resulting distance matrix using the R package "igraph". This network graph was used for community detection with the Leiden algorithm [94], implemented in the R package "leidenAlg".

The leiden.community function was used with a resolution parameter of 1.1, a minimum community size of three and the max.depth parameter set to eight to allow for fine-grain resolution. No resolution was gained beyond a recursive depth of three. The resulting dendrogram showed clear geographical structuring, however, when the leiden.community function was re-run using identical parameters, we observed some variability in the dendrogram topology. Thus, to find the best fitting dendrogram, we ran the leiden.community function 100 times with different seeds and constructed a consensus tree from the output. To do this, we converted the outputted dendrograms into "phylo" objects and inputted them into the maximum clade credibility (MCC) function available in the R package "phangorn". We note this MCC tree is not derived from a Bayesian posterior distribution, however, each dendrogram derives from a probability distribution with an expectation equal to the "true" dendrogram. The resulting consensus tree is shown in Figure 3. Further information on the individuals, archaeological sites and assigned leiden clusters can be found in Supplementary Table 17.

S6. Population Structure and Admixture

6.1 Projection PCA

To place the populations of Iron Age Britain into the broader European context, we performed PCA on a dataset of 5326 modern individuals from western Europe[95,96] using smartpca (version 16000) from EIGENSOFT[97]. We used a set of 265,948 variant sites for PCA, present in both the modern datasets and the 1240k SNP capture panel[93], commonly used for targeted capture in ancient genomic research. Pseudo-haploid genotypes from 534 Iron Age individuals from western Europe with over 40,000 genotype calls were then projected onto this PC space[34,39,41,43,46,47,70,98–100] (Supplementary Table 12).

Seven archaeological sites of interest had multiple samples available for analysis, however, all samples or all but one sample had too few SNP calls to project individually. To contextualise the genetic variation present at these sites, we created seven "merged" individuals, whereby genotype data from all samples from the site were merged into a single "hybrid" individual. These sites were:

1. Melton, England: I0525, I0527
2. Meare Lake Village, England: I11146, I13683, I13684
3. Knowe of Skea, Scotland: KD042, KD043, KD004
4. Llanmaes, Wales: I16471, I6771
5. Dinorben, Wales: I16514, I16410, I16475
6. Urville-Nacqueville, France: UN85, UN129
7. Longis Common, Channel Islands: I26628, I26629, I26630

To explore geographical trends in genetic variation, we took the median values observed along PC1 and PC2 for individuals from different regions of Britain and the continent (Extended Data Figure 2). For the European continent we categorised individuals by country and for Britain we categorised by county (Supplementary Table 12). We split French populations into northern France, Southern France and Channel Islands/La Manche. We also generated median values for single archaeological sites in Britain.

We find that populations from the western and northern peripheries of Britain, including Cornwall, Wales, Scotland, Northern England and the Midlands place further away from continental Iron Age variation. In contrast, populations from the south coast are closest to continental variation, reflecting connectivity across the English Channel also observed in haplotype analysis. Accordingly, Iron Age populations from the Channel Islands and the French region of La Manche in Normandy (Urville-Nacqueville[39]) place very close to southern English populations in PC space. We note that the three individuals from Urville-Nacqueville show evidence of ancestral diversity in SOURCEFIND analysis

(Extended Data Figure 8, Supplementary Note 6.3). Two low coverage samples had majority continental ancestry, while one higher coverage individual, UN19, possessed a majority component of British Bronze Age ancestry. This differentiation is reflected in pseudo-haploid PCA, where UN19 places centrally within British Iron Age variation, while a "merged" individual composed of UN85 and UN129 places close to the median of northern French populations.

To identify outliers within Iron Age Britain, we identified individuals who fall at least two standard deviations outside the mean value seen for British Iron Age populations along PC1 and PC2 (Extended Data Figure 2). We observe a number of outlying samples from Kent, Scotland and Yorkshire to fall towards the median values for Netherlands and Scandinavian Iron Age populations, hinting at North Sea connectivity. Two outliers from Winterborne Kingston fall towards French and Central European Iron age populations, one of which, WBK02, was confirmed to be a genetic outlier with SOURCEFIND.

6.2 qpADM

The Early Bronze Age population of Britain derives its ancestry from three distinct sources. These are (1) the Mesolithic hunter-gatherers of Europe, also known as western hunter-gatherers (WHG), (2) the first Neolithic farmers of Europe, also known as early European farmers (EEF), and (3) steppe pastoralists from the Pontic-Caspian Steppe. A rise in EEF ancestry has been observed across the Late Bronze Age in Britain (1000 - 750 BC), driven by gene flow from continental (likely French) populations[34]. Although some regional variability in EEF ancestry was observed, no clear geographical trends were identified (e.g. correlation with latitude).

To place the Dorset population from the current study into the wider British context we followed the same approach implemented by Patterson et al. (2022[34]) to estimate the percentage of EEF ancestry in British ancient genomes. This allowed us to explore regional and temporal variation in continental ancestry. Each target individual was given a point date estimate, based on either the median probability of their radiocarbon date, taken from CALIB 8.2, or the midpoint value of their contextual date range (Supplementary Table 12). Individuals with point estimates greater than AD 250 were removed. We also removed target individuals that had high missingness (<50,000 sites called across the 1240k panel).

We ran qpAdm on each individual, using the below setup to estimate ancestry proportions. We avoided using targeted SNP capture data in our source and reference populations to avoid any potential biases. Both SNP capture and whole genome shotgun data were used for target individuals.

The source (left) populations were:

1. WHGA (n=13) A set of Mesolithic individuals from France and northwest Europe[38,101,102].
2. Yamnaya (n=6) A set of Yamnaya individuals from the western Eurasian steppe[99].
3. EEF (n=9) A set of Early Neolithic Europeans from Greece, Hungary and Germany[101,103–105].

The reference outgroups (right) populations were:

1. WHGB (n=6) A set of Mesolithic individuals from Latvia and Romania with ancestry similar to WHGA[80,106]
2. Afanasievo (n=4) A set of individuals from the Altai region with ancestry very similar to Yamnaya[99].
3. Turkey_N (n=11) A set of Anatolian Neolithic farmers with ancestry similar to EEF[107].
4. Mbuti (n=10) A set of modern-day individuals indigenous to the Congo region with no evidence of recent Eurasian admixture[108].

More details on the reference and source populations can be found in Supplementary Table 15. We obtained similar estimates of EEF ancestry to those reported by Patterson et al. 2022 (Supplementary

Table 12). However, while Patterson et al. observed no rise in EEF ancestry through the Iron Age, we observe a significant increase in EEF ancestry in the Late Iron Age. This is driven by the addition of samples from the current study and is retained when we further remove samples with point date estimates greater than AD 50 (Supplementary Table 25). When the newly sequenced samples reported here are removed from our dataset, we observe no significant increase.

<table>
<tr><th>Time Period</th><th>Patterson et al. 2022</th><th>Current Study with new IA samples</th><th>Significant increase?</th><th>Current Study (new IA samples removed)</th><th>Significant increase?</th></tr>
<tr><td>Copper/Early Bronze Age (CEBA)
2450-1550 BC</td><td>31.0 ± 0.5%</td><td>33.1 ± 0.6%</td><td>-</td><td>-</td><td>-</td></tr>
<tr><td>Middle Bronze Age (MBA)
1550-1150 BC</td><td>34.7 ± 0.6%</td><td>35.6 ± 1.0%</td><td>Yes
(p=0.0439)</td><td>-</td><td>-</td></tr>
<tr><td>Late Bronze Age (LBA)
1150-750 BC</td><td>36.1 ± 0.6%</td><td>37.4 ± 1.2%</td><td>No
(p=0.2426)</td><td>-</td><td>-</td></tr>
<tr><td>Early/Middle Iron Age (EMIA)
750-100 BC</td><td rowspan="2">37.9 ± 0.4%</td><td>39.7 ± 0.2%</td><td>Borderline
(p=0.0637)</td><td>39.6 ± 0.2%</td><td>Borderline
(p=0.0752)</td></tr>
<tr><td>Late Iron Age (LIA)
100 BC - AD 50</td><td>41.8 ± 0.5%</td><td>Yes
(p=0.0005)</td><td>40.1 ± 0.9%</td><td>No
(p=0.5858)</td></tr>
</table>

Supplementary Table 25. Estimated EEF ancestry in the English and Welsh population for different archaeological periods. The mean percentage EEF ancestry and standard error are provided for each period. Time periods are based on those used by Patterson et al. (2022) and previously calculated estimates from Patterson et al. (2022) are shown. We further divide the Iron age into an Early/Middle and Late period. Welch's *t*-test (two-tailed) is used to test for differences in EEF ancestry between time periods. We observe a significant increase in EEF ancestry in the Late Iron Age (red), driven by the addition of new samples from the current study.

The above result implies additional and substantial gene flow into Britain from continental populations during the Iron Age period. This gene flow may have been regionally restricted. To explore further, we divided our dataset into a "channel core" region, encompassing populations south of the Thames, from the Durotrigian zone in the west to the Kentish coast in the east (below latitude 51.5° and east of longitude -2.8°), and a "peripheral" region, encompassing all other English and Welsh populations. We plot the percentage of EEF ancestry in each genome their point date estimate in Figure 3c. We fit a local polynomial regression line (loess) to the data, which reveals separate trajectories of EEF ancestry in the channel core region and peripheral zones. In the channel core, EEF ancestry rises rapidly after the Early Bronze Age, plateauing across the Late Bronze Age, before rising again during the Middle to Late Iron Age. In the peripheries, no increase is seen until the Late Bronze Age to Early Iron Age, with the amount of EEF ancestry remaining stable after the Early Iron Age.

To test the robustness of this signal, we further divided our dataset into seven subregions (Extended Data Figure 7). The "channel core" region was split into three coastal zones from east to west, while the peripheral regions were split into southwestern, northern and central English groupings. We also included Scotland as the seventh region. To characterise changes in EEF ancestry, we took two approaches. First, we binned genomes into 500-year intervals. For ease, we label these bins using the approximate archaeological era they overlap with, although we emphasise these date ranges do not hold any particular archaeological significance: 250 BC - AD 250 (Later Iron Age; LIA), 750-250 BC (Earlier Iron Age; EIA), 1250-750 BC (Later Bronze Age; LBA), 1750-1250 BC (Middle Bronze Age; MBA), 2500-1750 BC (Earlier Bronze Age; EBA). Second, we plot a rolling average of EEF for each region, using a window size of 500 years and a step size of 50 years.

We discuss the results for each region in turn below.

1. Scotland (marked green in Extended Data Figure 7): No significant changes in EEF ancestry are seen through time, in agreement with the observations of Patterson et al. 2022.

2. Northern England (blue): EEF ancestry remains stable from the Early to Late Bronze Age. We find no evidence for an increase in EEF ancestry until the Early Iron Age. Indeed, Late Bronze Age samples from Raven Scar Cave (contextually dated to 1090-900 BC) and Melton (1200-933 cal BC), show a slight decrease in EEF ancestry relative to previous periods. The earliest Iron Age samples, from Carsington Pasture Cave in Derbyshire (CE033/I12274; 757-416 cal BC) and Pockington in Yorkshire (I11033; 717-395 cal BC), show a sharp increase in EEF ancestry. Haplotypic analysis suggests that the Carsington Pasture individual is not local to Britain, with close to zero ancestral contribution from preceding Bronze Age populations (Extended Data Figure 3). No significant changes in EEF ancestry are seen following the Early Iron Age.

3. Southwest England and Wales (light pink): Very few samples are available from Wales, Cornwall and bordering regions. We do not observe any significant changes in EEF ancestry across the Bronze Age. When comparing sample bins, the only significant difference is seen between the 2500-1750 BC bin and the 250 BC - AD 250 bin. This implies gene flow into the region during the Late Bronze Age and/or Iron Age. An increase in EEF ancestry is seen between the Early and Late Iron Age but this is insignificant.

4. Central England (Orange): Unlike the "channel core" regions south of the Thames, we do not see any increase in EEF ancestry across the Early to Middle Bronze Age transition in this region. Instead, we see EEF increase through the Late Bronze Age and Early Iron Age. No significant change is seen after the Early Iron Age.

5&6. South Central England: We consider two neighbouring coastal regions centred on Dorset (Dark Red) and Hampshire (Red). Both have very similar trajectories in EEF, although very few Bronze Age samples from the Dorset region are available. We observe a significant increase in EEF ancestry from the Early Bronze Age to the Middle Bronze Age in Hampshire, resulting in this region having the highest levels of EEF ancestry in Britain at this time. Thus, it is plausible that migration from this region contributed to increases in EEF ancestry seen in other areas of Britain during later periods. No further change in EEF ancestry is seen until the Late Iron Age. Both regions show a significant increase between the 750-250 BC and 250 BC- AD 250 bins (p-values of 0.023 and 0.003 for Hampshire and Dorset respectively). This increase is still significant if we remove samples whose point date estimates fall after AD 1 (p-values of 0.023 and 0.036).

7. Southeast England (yellow): EEF ancestry climbs in this region from the Early Bronze Age to the Late Bronze Age. Similar to the two other "channel core" regions, and in contrast to the "peripheral" regions, no significant change in EEF ancestry is seen across the Late Bronze Age to Early Iron Age transition. However, unlike the other "channel core" regions, we do not detect any further increases in EEF ancestry in the Late Iron Age.

6.3 ChromoPainter, fineSTRUCTURE and SOURCEFIND

It has been demonstrated through allele-frequency-based analysis that England experienced inward migration from continental Europe during the Middle Bronze Age to Late Bronze Age[34]. This study estimated that approximately half the gene pool of England in the Iron Age derived from these migrants and that Britain experienced a substantial degree of genetic isolation from continental Europe during the Iron Age.

Here, we apply haplotype-based methods to characterise patterns of gene flow into Britain in both the Bronze and Iron Age. These methods provide higher resolution of population structure and admixture compared to allele-frequency-based approaches, particularly among populations that show relative genetic homogeneity[95]. Specifically, we implement two different methods for inferring ancestry proportions in genomes, which are applied to the output of ChromoPainter software[109].

ChromoPainter reconstructs individual genomes (recipients) using haplotypic chunks donated by other individuals (donors), resulting in an asymmetric co-ancestry matrix that summarises the amount of haplotype donation between pairs. Ancestry proportions in "target" individuals or populations that are contributed by different "surrogate" populations can then be calculated from this matrix using (1) non-Negative least squares (NNLS) regression[95,110] or (2) a Bayesian model, SOURCEFIND[111], which eliminates contributions that cannot be reliably distinguished from background noise. To test the robustness of our results, we used different sets of surrogate populations for NNLS and SOURCEFIND. More details on each approach are provided in the following sections.

Dataset preparation

A dataset of 1552 individuals from the European Bronze Age to medieval period (WGS+SNPCAP3) had previously been subject to three independent runs of Beagle5 imputation and phasing (Supplementary Figure 6; Supplementary Note 3), with the resulting genotypes filtered for biallelic SNPs present in the 1240k SNP capture[93] and with a minor allele frequency (MAF) > 0.01 in the dataset. The filtered output from the first run was subsequently subject to SHAPEIT2 (v2.r837) phasing[112] and then converted into ChromoPainter format using scripts available from the software's website (http://www.paintmychromosomes.com/). The individuals were then subject to further filtering depending on the downstream analysis.

1. **Removing Relatives:** To identify relatives, we ran refinedIBD on each of the three filtered datasets outputted from Beagle5. For each run, small gaps between IBD segments were removed using the merge-ibd-segments.17jan20.102.jar program (default parameters). These segments were then filtered for a LOD score ≥ 30 and a length ≥ 4 cM. The union of segments across runs was then determined with bedtools[83] and centimorgan positions added using an in-

house script. Pairs of individuals with >200cM shared were flagged as closely related. We then used an in-house script to iteratively remove individuals with the largest number of relatives, until no related pairs were left. This provided us with a list of individuals to remove from downstream ChromoPainter runs to prevent excessive painting between relatives.

2. **Missingness:** Prior to Beagle imputation, each individual had been subject to GLIMPSE imputation and the outputted genotypes had been filtered for 1240k positions and a genotype probability (GP) > 0.99. Lower coverage samples that had imputed less accurately possessed higher levels of genotype missingness following this GP filter. Only samples with a minimum individual missingness (MIND) below 0.4 were subject to Beagle imputation. We further filtered our SHAPEIT phased dataset by MIND values of 0.15 and 0.25, depending on the type of ChromoPainter analysis. We emphasise that there were no missing genotypes in the dataset inputted into SHAPEIT, as these had been re-imputed by Beagle5.

ChromoPainterv2 requires switch rate (-n) and global mutation rate (-M) parameters to be provided. To estimate the appropriate values to use for this dataset, we followed the software's guidelines and ran 10 E-M iterations on 50 individuals in the dataset for chromosomes 1, 2, 21 and 22. We then took the final estimated values for each chromosome and averaged to get the final switch (-n 200) and global mutation rates (-M 0.00015).

Ancestry profiles generated using NNLS

For this approach, we used a dataset of 332 individuals (MIND<0.15, relatives removed) from Bronze and Iron Age Europe as our surrogates, including British Bronze Age individuals. These were grouped into 17 populations using fineSTRUCTURE[109] (Supplementary Table 12). Both whole genome shotgun (WGS) sequence data and SNP capture (SNPCAP) data were included.

We generated a co-ancestry matrix of donated haplotypic chunk counts for this set of 332 individuals using ChromoPainterv2. We then ran fineSTRUCTURE's MCMC model on this matrix for 1,000,000 million burn-in iterations and 2,000,0000 sampling iterations, sampling every 10,000 iterations. We then used a previously described maximum concordance tree-building method[95], whereby individuals are reassigned to their optimal cluster based on cluster membership across all MCMC samples. This resulted in 17 clusters being identified, which showed good geographic and temporal integrity (Supplementary Figure 28, Supplementary Table 12). Nearly all individuals from Bronze Age Britain (Early to Late) were grouped within a single cluster (n=54), which also contained 18 continental genomes that mostly derived from the Early Bronze Age of northern Europe (Germany, Netherlands, Poland, Czechia).

Next, we painted the genomes of 270 British individuals dating from *c.* 800 BC - AD 400 (MIND<0.25, relatives included) using these 17 populations. This resulted in a vector of haplotype donations from the 17 populations for each British genome. The non-negative least squares (NNLS) approach described in Leslie et al. 2015[95] was then used to generate ancestry profiles for each individual. To reduce noise, we then grouped samples by archaeological site, summing haplotype contributions from each of the 17 fineSTRUCTURE clusters across individuals. This resulted in a new vector, which was used to reconstruct an ancestry profile for the population buried at each archaeological site through NNLS.

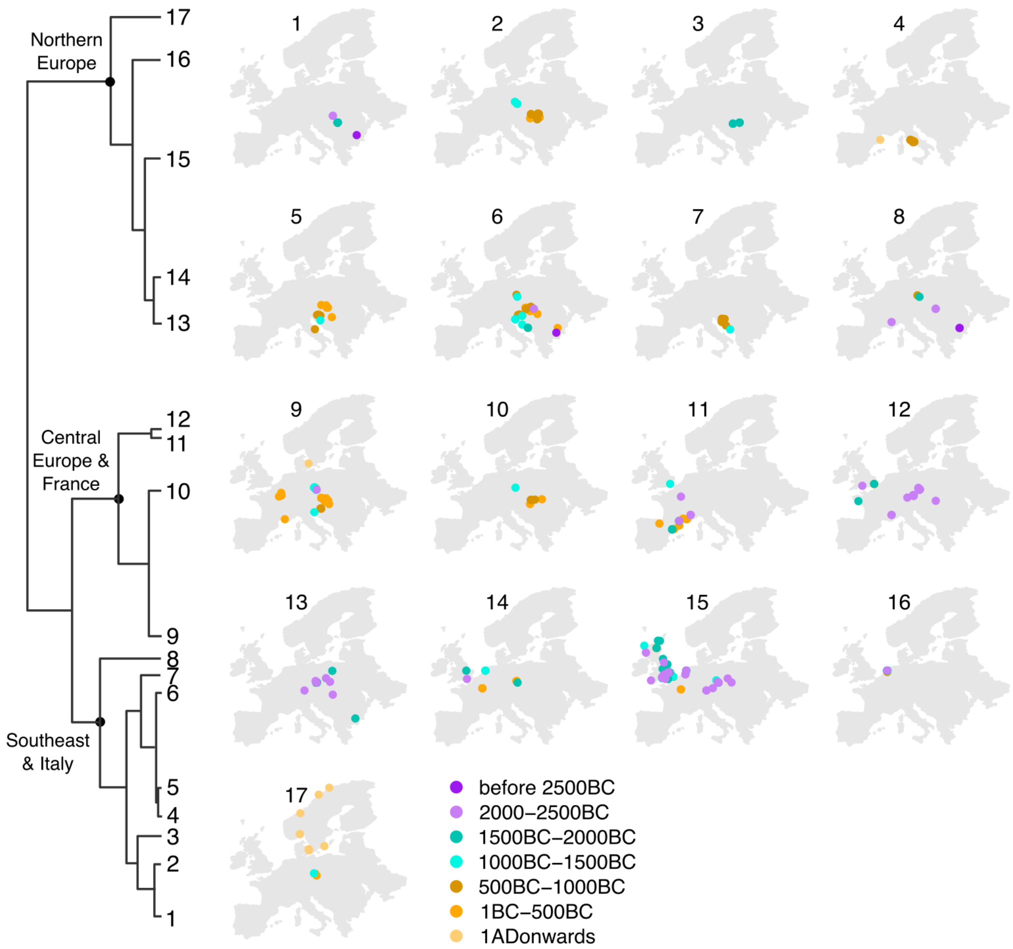

Supplementary Figure 28. Population structure of Bronze Age and Iron Age Europeans. Individuals were grouped into 17 populations using fineSTRUCTURE. Each panel shows the geographic and temporal distribution of individuals within each cluster. Cluster 15 contains the majority of British Bronze Age individuals alongside a number of Early Bronze Age samples from northern Europe. The dendrogram reveals the 17 populations to cluster in broader geographical groupings, corresponding approximately to northern Europe, Central Europe and France, and southeastern Europe and Italy.

Ancestry profiles generated using SOURCEFIND

It has been shown through simulations that SOURCEFIND has greater accuracy than NNLS[111]. For this approach, we used a different set of individuals (MIND<0.15, relatives removed) from Bronze Age, Iron Age and medieval Europe as our surrogates. Specifically, here we only included SNP capture data in our surrogate dataset as the use of two different data types may introduce batch effects to the chromopainting process. We did not include British Bronze Age genomes post-dating 1500 BC as surrogates to allow us to generate ancestry profiles for these individuals. An Iron Age sample from the Netherlands was removed for the same reason.

This resulted in a dataset of 307 surrogates. We then generated a co-ancestry matrix of donated haplotypic chunks for these individuals, as well as 19 medieval Irish individuals[79], who were not used as surrogates given the confounding impact of later gene flow from Britain to Ireland. The surrogate individuals were then grouped into populations on the basis of geography and time period. Here the Scottish Bronze Age population and the English Bronze Age population were treated as separate surrogates. These were used to paint the genomes of 390 individuals (MIND<0.4, relatives included) derived mostly from the British Iron Age. Both whole genome sequence (WGS) and SNP capture data were painted. We also painted one genome from the Netherlands Iron Age, as well as a set of French Iron Age genomes generated using WGS and thus excluded from the surrogate dataset.

SOURCEFIND was run using 50,000 burn-in iterations followed by 200,000 sample iterations, thinning every 5,000 iterations. We set the expected number of surrogates used to form the target as two, with a total number of four surrogates allowed to form the target in each iteration. We carried out 50 independent runs of the above procedure and extracted the estimates with the highest posterior probability in each run. The average of these 50 estimates (weighted by posterior probability) was then calculated for each individual. This provided us with a set of ancestry proportions for each genome (Supplementary Table 17).

We aggregated these estimates for 325 genomes with a MIND<0.25 for archaeological sites and larger geographical regions.

Comparing the output of NNLS and SOURCEFIND

To check for consistency between NNLS and SOURCEFIND results, we compared the amounts of British Bronze Age ancestry in each British Iron Age genome estimated using the two different approaches (Supplementary Figure 30). We observed a strong correlation. In both analyses, highest levels of British Bronze Age ancestry were observed in Scottish populations, while lowest values were found in southern coastal regions. If we consider all British genomes painted, ranging from *c.* 800 BC - AD 250 in England/Wales and *c.* 600 BC - AD 400 in Scotland, we estimate the mean contribution of

earlier British Bronze Age ancestry as 75.56 ± 1.24 % based on NNLS and 72.73 ± 1.19 % based on SOURCEFIND. For English and Welsh Iron Age genomes, these estimates dropped to 73.85 ± 1.27 % and 71.14 ± 1.22 % respectively. For Scottish Iron Age genomes, these estimates were 98.07 ± 1.31 % and 90.19 ± 1.95 %. This is in agreement with previous findings that demonstrated population movement into England, but not Scotland at the end of the Bronze Age[34]. To control for any later movement into Britain during the Roman period we also calculate these estimates removing all samples with a point date estimate later than AD 50 for the seven geographical regions used in qpAdm analysis (Supplementary Table 16), as well as the twelve IBD clusters identified using the Leiden algorithm (Figure 1a; Supplementary Data Figure 29). Both divisions of the dataset reveal the Iron Age populations of Hampshire and surrounding regions to possess the lowest average contributions from the British Bronze Age population.

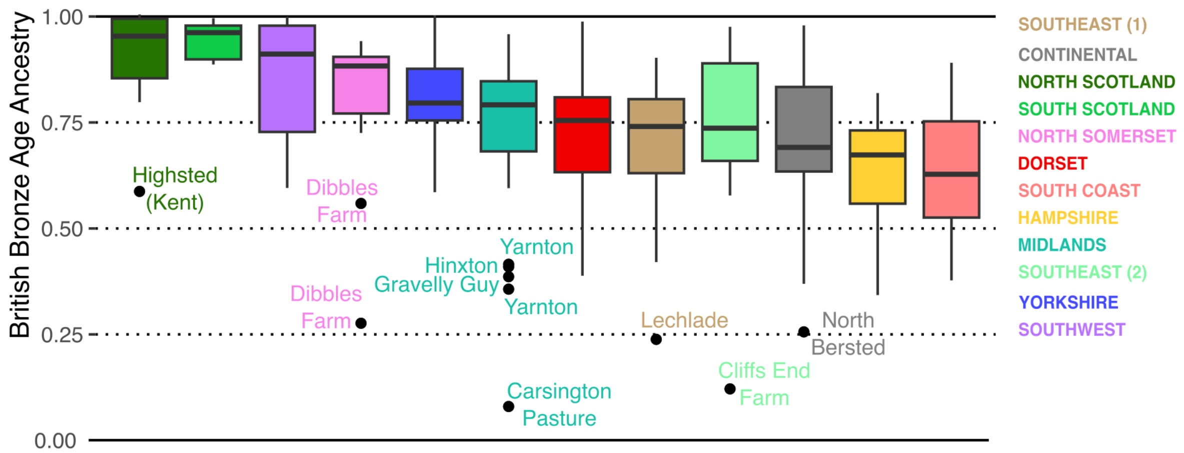

Supplementary Figure 29. Proportion of ancestry derived from the British Bronze Age in British Iron Age Leiden clusters. Haplotypic contributions from the British Bronze Age population to later genomes, grouped by IBD cluster (see Fig. 3a). Haplotypic contribution is an average of that estimated using SOURCEFIND and NNLS approaches with different surrogate population panels. Outlying genomes are labelled by their archaeological site. Exact values and sample numbers for each grouping are available in Supplementary Table 17.

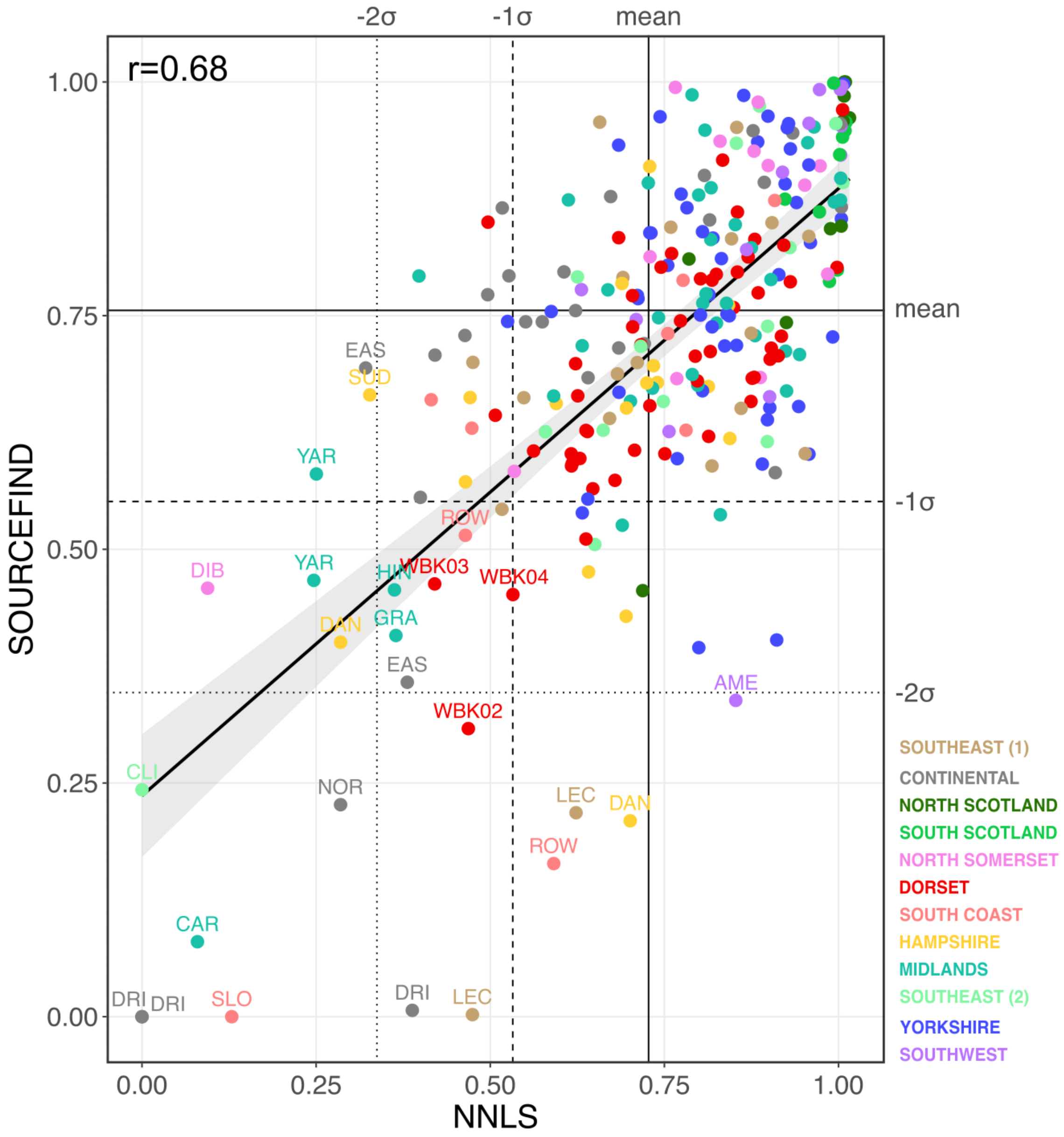

Supplementary Figure 30. Proportion of ancestry derived from the British Bronze Age in British Iron Age Genomes. Two different pipelines were used to estimate the haplotypic contribution of the British Bronze Age population to later genomes. These were based on SOURCEFIND and NNLS approaches with different surrogate population panels used. Individuals are coloured based on IBD clusters identified in Figure 3a. Individuals with low levels of British Bronze Age ancestry are labelled by the first three letters of their archaeological site. We label individuals two standard deviations below the mean in either NNLS or SOURCEFIND analysis. We also label individuals that are one standard deviation below the mean in both analyses. Three WBK outliers are emphasised.

We also compared British Bronze Age contributions to the ancestry profiles of different archaeological sites (Supplementary Figure 31). Again, we observed a strong correlation between estimates from the SOURCEFIND and NNLS pipelines. Four sites had markedly low levels of British Bronze Age ancestry. One of these sites, Driffield Terrace, dates to the Roman era, while another, Slonk Hill, includes an individual with a wide contextual date range (700 BC - AD 900). SOURCEFIND estimates this Slonk Hill individual to have zero contribution from the British Bronze Age population, as do three of the Driffield Terrace individuals (discussed further below). Given their post-Iron Age dates and outlying ancestral profiles, these individuals were removed from further downstream analysis.

To explore the geographical distribution of British Bronze Age ancestry in Iron Britain, we took the average of the NNLS and SOURCEFIND contributions for each archaeological site. Inverse Distance Weighting (IDW) Interpolation was carried out using the *gstat* package in R to estimate the distribution of British Bronze Age ancestry through space. Weights were calculated using the default method ("Shepard").

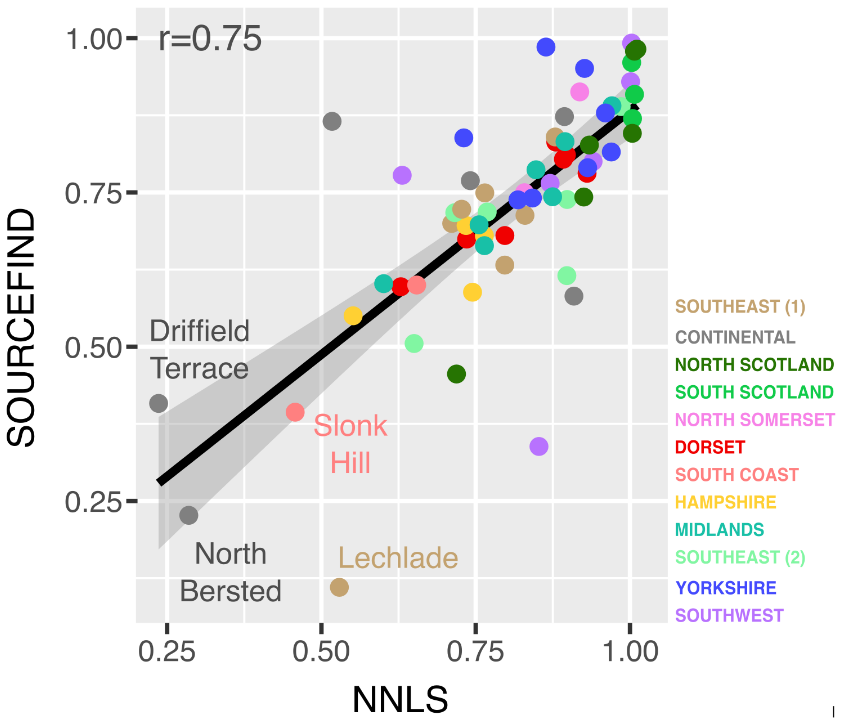

Supplementary Figure 31. Proportion of ancestry derived from the British Bronze Age in British Iron Age archaeological sites. Two pipelines, based on SOURCEFIND and NNLS, were used to generate ancestry profiles for British Iron Age populations at different archaeological sites. The estimated haplotypic contribution of the British Bronze Age population to these ancestry profiles is shown for both pipelines. A strong correlation is observed, with four outlying sites identified in both analyses highlighted.

Comparing the output of qpAdm and SOURCEFIND

Increases in EEF ancestry in Britain are a good indicator of continental gene flow[34], given the higher levels of EEF ancestry in French and Central European populations. Accordingly, when we plot EEF ancestry against British Bronze Age haplotypic contribution (estimated by SOURCEFIND) we observe a moderate negative correlation (Supplementary Figure 31). However, EEF ancestry is an imperfect predictor of continental ancestry. We observe a degree of heteroscedasticity, whereby the squared residual size is positively correlated with the level of EEF ancestry. At the higher range of EEF ancestry, we observe a large number of outliers who possess much lower British Bronze Age ancestry than would be predicted on the basis of EEF ancestry. Thus, as EEF ancestry increases through time in Britain, our ability to detect further gene flow and non-local individuals decreases. We can expect such an effect to have the largest impact on southern regions of England where EEF ancestry is highest. Accordingly, when we regress average British Bronze Age haplotypic contribution against EEF ancestry for Iron Age archaeological sites, we see large negative residuals concentrated in the "channel core" region (Supplementary Figure 32).

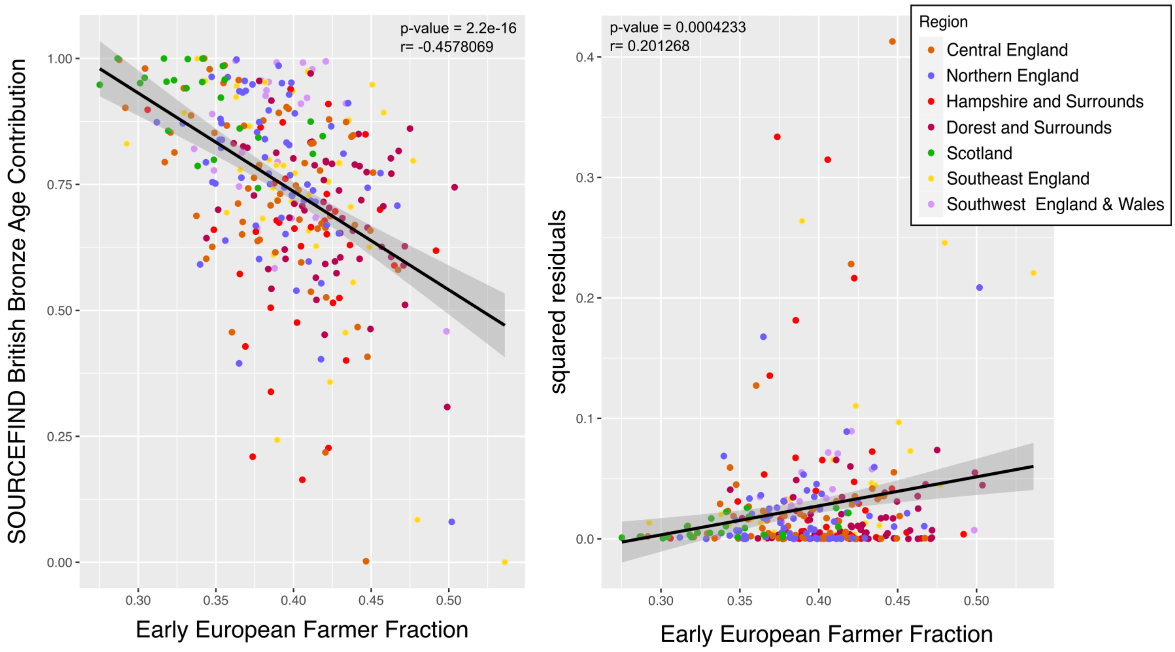

Supplementary Figure 32. Comparing qpAdm estimates of EEF ancestry to SOURCEFIND estimates of British Bronze Age ancestry. Genomes are coloured based on geographic region (see Extended Data Figure 7). A moderate negative correlation is observed (right panel). The squared residual size increases as a function of proportion of EEF ancestry.

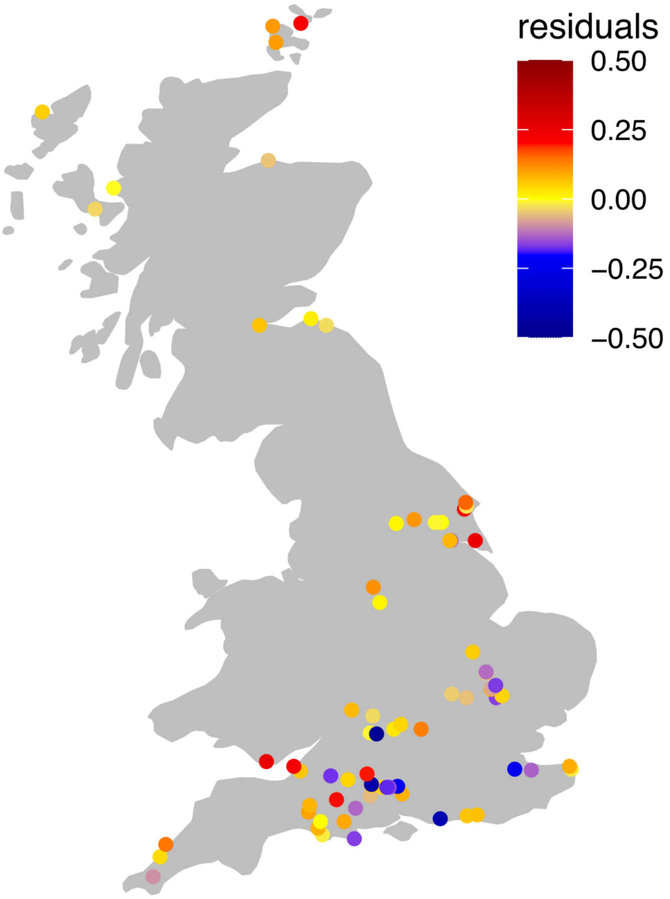

Supplementary Figure 33. EEF ancestry underpredicts continental ancestry in southern regions. We regress the average amount of British Bronze Age ancestry (estimated by SOURCEFIND) against EEF ancestry for Iron Age archaeological sites and plot the resulting residuals. These show lower levels of British Bronze Age ancestry (large negative residuals) in the channel core region than would be predicted from EEF ancestry alone.

British Bronze Age ancestry through time

To explore changes in the ancestral composition of British populations through time, we plot collated SOURCEFIND ancestry profiles for the same geographical regions and time bins used for qpAdm analysis. Due to stricter coverage thresholds for imputation and haplotypic analysis, this dataset is only 60% of the size used for qpAdm. However, despite this loss of power, similar temporal trends emerge to those observed for EEF ancestry proportions. As the British Early Bronze Age population are used as surrogates, we are only able to plot changes from the Middle Bronze Age onwards (Supplementary Figure 33).

We observe little change in Scotland through time and find that the Scottish Early Bronze Age is the major contributor to later Scottish populations. To explore further, we plot the relative difference between Scottish and English Early Bronze Age ancestry for Iron Age archaeological sites and find a marked distinction between Scotland and the south of Britain (Supplementary Figure 34).

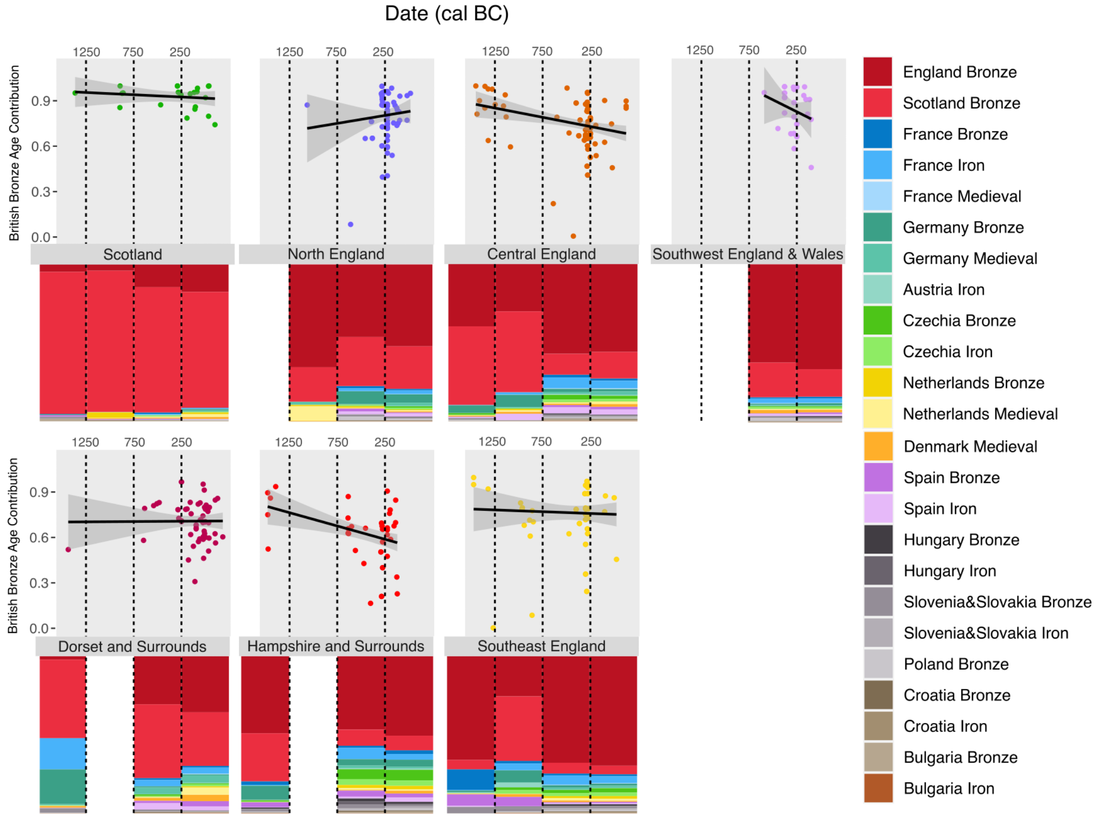

Supplementary Figure 34. SOURCEFIND ancestry profiles and estimates of British Bronze Age haplotypic contributions through time. We group samples into the same geographical and temporal time bins as used for qpAdm analysis (see Extended Data Figure 7). Surrogate and target individuals are listed with their population IDs in Supplementary Table 12.

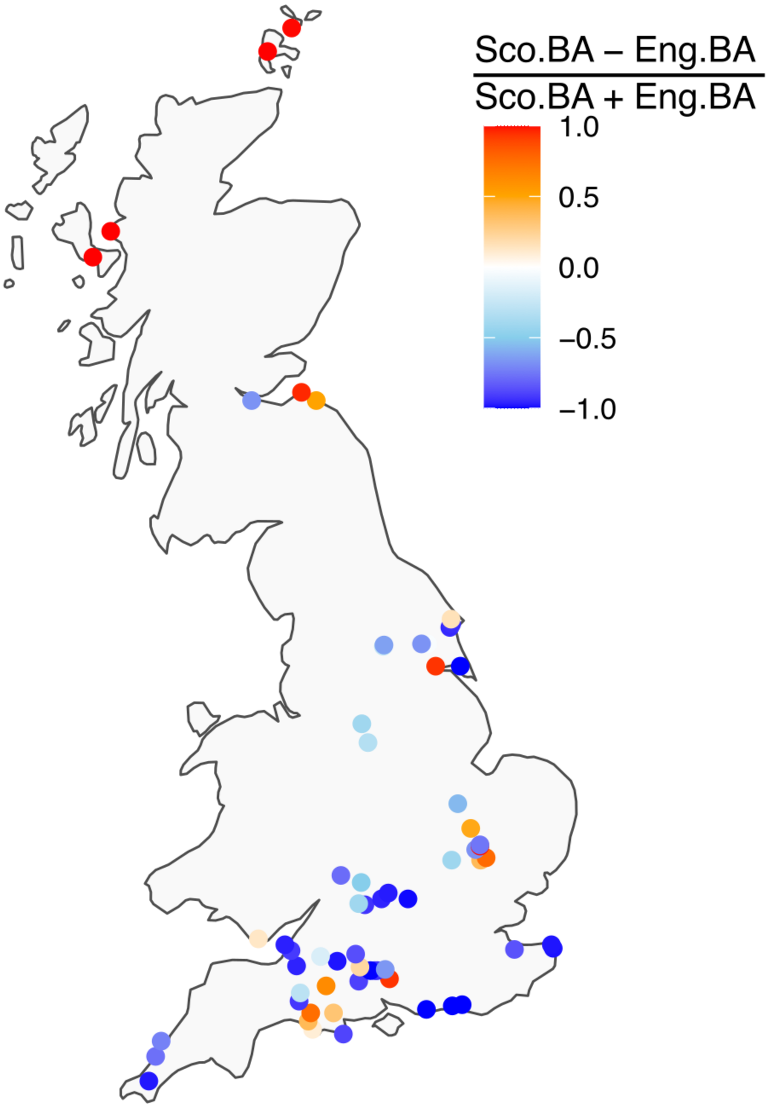

Supplementary Figure 35. Relative difference in Scottish and English Early Bronze Age ancestry for Iron Age archaeological sites. We take the difference in SOURCEFIND contributions from Scottish and English Early Bronze Age genomes and divide by the sum. The median value for each Iron Age site is plotted. A value of 1 indicates that the British Early Bronze Age contribution is entirely from the Scottish Early Bronze Age, while a value of -1 indicates that it is entirely from the English Early Bronze Age. We see highest relative contributions from the Scottish Early Bronze Age in the North of Scotland and from the English Early Bronze Age in the south of England.

A note on genetic outliers

There were several archaeological sites and individual genomes that showed outlying levels of continental ancestry (highlighted in Supplementary Figures 30 and 31). We further investigated by plotting SOURCEFIND ancestry profiles for all targeted Bronze and Iron Age archaeological sites in Britain, France and the Netherlands. We plot individual ancestry profiles for genomes with SOURCEFIND estimates of British Bronze Age ancestry two standard deviations below the mean (Extended Data Figure 3). We discuss sites of interest in turn below.

Driffield Terrace, Yorkshire: This is a Roman cemetery from which the genomes of six adult males, possibly soldiers, have been sequenced[47]. SOURCEFIND found three of these men to have close to zero contribution from the British Bronze Age population. Two of these individuals have previously been identified as outliers. 3DT26 derives their ancestry from the Middle East [47], while 6DT3 has been shown to have higher affinity to continental populations through rare allele sharing and IBD-based analyses[50,113]. The third individual, 3DT16, to our knowledge, has not before been identified as an outlier. SOURCEFIND estimates his ancestry to derive from Medieval and Bronze Age populations of Germany and the Netherlands.

Slonk Hill, Sussex: We identify one individual at Slonk Hill (I14550[34]) whose ancestry is estimated to derive almost entirely from the Netherlands Medieval population. This individual was flagged as having an uncertain date range that overlaps with the Medieval period[34]. The burial context suggests a Late Roman or Early Medieval date.

North Bersted, Sussex: This genome is from a male warrior burial (30-45 years) with elaborate grave goods[34,114]. The burial dates to the La Tène period, *circa* 150-50 BC and has an isotopic signature suggestive of a non-local origin. SOURCEFIND estimates his ancestry to derive mainly from Czech Bronze and Iron Age populations.

Lechlade Memorial Hall. Gloucestershire: Two outlying individuals were identified from this site (I12783, I12787), one of whom had zero contribution from British Bronze Age populations. Top contributions were instead from French and Czech sources. These genomes derive from two Early Iron Age crouched burials[34], comprising a woman and adolescent male.

Carsington Pasture Cave, Derbyshire: One genome (I12774) in this sample set is an outlier with respect to date (Early Iron Age) and ancestry[34]. SOURCEFIND estimates this male individual's ancestry to derive mainly from Spanish and French populations, in agreement with his positioning on PCA close to the median value for the Spanish Iron Age population. This individual was previously

identified as an outlier with respect to EEF ancestry[34]. This is suggestive of mobility along the Atlantic seaboard in the Early Iron Age.

Rowbarrow, Wiltshire: An Early Iron Age genome from Rowbarrow (I19863) shows majority contributions from German and Czech sources. This individual was previously identified as an outlier with respect to EEF ancestry[34].

Danebury and Amesbury Down, Hampshire: Two Middle to Late Iron Age individuals from Amesbury (I16601) and Danebury (I20990) show majority contributions from German, Czech and Netherlands sources.

East Kent Access Road and Cliffs End Farm, Kent: Two middle Iron Age individuals from these sites (I19907 and I14863) show majority contributions from French, German, Czech and Netherlands sources.

Winterborne Kingston, Dorset: We observe three individuals from Winterborne Kingston with SOURCEFIND estimates of British Bronze Age ancestry one standard deviation below the mean (WBK02, WBK03 and WBK04). WBK02 is the most pronounced outlier with his continental ancestry modelled as coming almost entirely from the Spanish Bronze Age.

Cliffs End Farm and Margetts Pit, Kent: While the majority of tested Middle and Late Bronze Age genomes show only minor contributions from continental sources, two extreme outliers in Kent are observed from Margetts Pit (I13716l; 1391–1129 cal BC) and Cliffs End Farm (I14861; 912-808 cal BC). These individuals were previously identified as outliers with respect to EEF ancestry and used as the proxy source for incoming continental ancestry during the Middle and Late Bronze Age in Patterson et al. 2022[34]. This study found these outliers were genetically similar to individuals from the Knoviz culture of central Europe, a subgroup of the Urnfield cultural complex (1300-800 BC). SOURCEFIND models the ancestry of the Margetts Pit individual as a mix of French and Spanish Bronze Age components. The Cliffs End individual also has a substantial contribution from the Spanish Bronze Age, as well as German and Czech sources.

References

1. Harding, D. *The Iron Age in Lowland Britain*. (Routledge and Kegan Paul, London, 1974).
2. *Introduction to British Prehistory*. (Leicester University Press, Leicester, 1979).
3. Cunliffe, B. W. *Iron Age Britain*. (Batsford, London, 1995).
4. Bradley, R. *The Prehistory of Britain and Ireland*. (Cambridge University Press, Cambridge, 2007).
5. Darvill, T. *Prehistoric Britain (2nd Edition)*. (Routledge, Abingdon, 2010).
6. Sharples, N. *Social Relations in Later Prehistory*. (Oxford University Press, Oxford, 2010).
7. Whimster, R. *Burial Practices in Iron Age Britain: A Discussion and Gazetteer of the Evidence C. 700 BC – AD 43*. (British Archaeological Reports 90, Oxford, 1981).
8. Harding, D. W. *Death and Burial in Iron Age Britain*. (Oxford University Press, 2016).
9. Whimster, R. Iron Age Burial in Southern Britain. *Proceedings of the Prehistoric Society* **43**, 317–328 (1977).
10. Gale, J. *Prehistoric Dorset*. (Tempus, Stroud, 2003).
11. Papworth, M. *The Search for the Durotriges: Dorset and the West Country in the Late Iron Age*. (History Press, Stroud, 2011).
12. Stewart, D. & Russell, M. *Hillforts and the Durotriges: A Geophysical Survey of Iron Age Dorset*. (Archaeopress, Oxford, 2017).
13. Gerdau-Radonic, K., Sperrevik, J., Smith, M., Cheetham, P. & Russell, M. Deathways of the Durotriges: reconstructing identity through archaeothanatology in later Iron Age southern Britain. in *The Routledge Handbook of Archaeothanatology* (eds. Schotsman, E. & Knüsel, C.) 243–262 (Routledge, London, 2021).
14. Papworth, M. *Deconstructing the Durotriges: A Definition of Iron Age Communities within the Dorset Environs*. (British Archaeological Reports, Oxford, 2008).
15. Russell, M. *et al.* The Durotriges Project, phase one: an interim statement. *Proc. Dorset Nat. Hist. Archeol. Soc.* **135**, 217–221 (2014).
16. Aitken, G. & Aitken, N. Excavations at Whitcombe, 1965-1967. *Proc. Dorset Nat. Hist. Archeol.*

Soc. **112**, 57–94 (1990).

17. Russell, M., Smith, M., Cheetham, P., Evans, D. & Manley, H. The Girl with the Chariot Medallion: a well-furnished, Late Iron Age Durotrigian burial from Langton Herring, Dorset'. *Archaeological Journal* **176**, 196–230 (2019).
18. Wheeler, M. *Maiden Castle*. (Society of Antiquaries Report 12, Oxford University Press, Oxford, 1943).
19. Bailey, J. An Early Iron Age / Romano British site at Pins Knoll, Litton Cheney. *Proc. Dorset Nat. Hist. Archeol. Soc.* **89**, 147–159 (1967).
20. Russell, M. *et al.* The Durotriges Project 2016: an interim statement. *Proc. Dorset Nat. Hist. Archeol. Soc.* **138**, 105–111 (2017).
21. Fitzpatrick, A. A 1st-century AD Durotrigian inhumation burial with a decorated Iron Age mirror from Portesham, Dorset. *Proc. Dorset Nat. Hist. Archeol. Soc.* **118**, 51–70 (1997).
22. Davies, S., Bellamy, P., Heaton, M. & Woodward, P. *Excavations at Alington Avenue, Fordington, Dorchester, Dorset, 1984-87*. (Dorset Natural History and Archaeological Society Monograph 15, Dorchester, 2002).
23. Valentin, J. Manor Farm, Portesham: excavations on a multi-phase religious and settlement site. *Proc. Dorset Nat. Hist. Archeol. Soc.* **125**, 23–70 (2003).
24. Akerman, J. Some antiquities discovered at Spettisbury, near Blandford, Dorset. *Proceedings of the Society of Antiquaries London* **4**, 188–191 (1859).
25. Gresham, C. Spettisbury Rings, Dorset. *Archaeological Journal* **96**, 115–131 (1939).
26. Russell, M. *et al.* The Durotriges Project, Phase Two: an interim statement. *Proc. Dorset Nat. Hist. Archeol. Soc.* **136**, 157–161 (2015).
27. Russell, M. *et al.* The Durotriges Project, phase three: an interim statement. *Proc. Dorset Nat. Hist. Archeol. Soc.* **137**, 173–177 (2016).
28. *Standards for Data Collection from Human Skeletal Remains*. (Arkansas Archaeological Survey Research Series No 44, Fayetteville, 1994).
29. McKinley, J. Compiling a skeletal inventory: disarticulated and co-mingled remains. in *Guidelines to the Standards for Recording Skeletal Remains* (eds. Brickley, M. B. & McKinley,

J.) 14–17 (BABAO, Institute of Field Archaeologists, Reading, 2004).

30. Eska, J. F. The emergence of the Celtic languages. in *The Celtic Languages* 36–41 (Routledge, 2009).
31. Eska, J. F. The dialectology of Celtic. in *Volume 2 Handbook of Comparative and Historical Indo-European Linguistics* (eds. Klein, J. & Joseph B, Fritz, M) (De Gruyter Mouton, 2017).
32. Sims-Williams, P. The Celtic languages. in *The Indo-European Languages* (ed. Kapović, M.) 352–86 (Routledge, Abingdon, 2017).
33. Falileev, A. I. *In Search of the Eastern Celts: Studies in Geographical Names, Their Distribution and Morphology*. (Archeolingua Alapítvány, 2014).
34. Patterson, N. *et al.* Large-scale migration into Britain during the Middle to Late Bronze Age. *Nature* **601**, 588–594 (2022).
35. Koch, J. T. & Cunliffe, B. *Celtic from the West 3: Atlantic Europe in the Metal Ages — Questions of Shared Language*. (Oxbow Books, 2016).
36. Stifter, D. 12. With the Back to the Ocean: the Celtic Maritime Vocabulary. *The Indo-European Puzzle Revisited: Integrating Archaeology, Genetics, and Linguistics* 172 (2023).
37. Sims-Williams, P. An alternative to 'Celtic from the East' and 'Celtic from the West'. *Camb. Archaeol. J.* **30**, 511–529 (2020).
38. Brace, S. *et al.* Ancient genomes indicate population replacement in Early Neolithic Britain. *Nat Ecol Evol* **3**, 765–771 (2019).
39. Brunel, S. *et al.* Ancient genomes from present-day France unveil 7,000 years of its demographic history. *Proc. Natl. Acad. Sci. U. S. A.* **117**, 12791–12798 (2020).
40. Cassidy, L. M. *et al.* A dynastic elite in monumental Neolithic society. *Nature* **582**, 384–388 (2020).
41. Dulias, K. *et al.* Ancient DNA at the edge of the world: Continental immigration and the persistence of Neolithic male lineages in Bronze Age Orkney. *Proc. Natl. Acad. Sci. U. S. A.* **119**, (2022).
42. Fernandes, D. M. *et al.* A genomic Neolithic time transect of hunter-farmer admixture in central Poland. *Sci. Rep.* **8**, 14879 (2018).

43. Fischer, C.-E. *et al.* Origin and mobility of Iron Age Gaulish groups in present-day France revealed through archaeogenomics. *iScience* **25**, 104094 (2022).

44. Fowler, C. *et al.* A high-resolution picture of kinship practices in an Early Neolithic tomb. *Nature* **601**, 584–587 (2022).

45. Freilich, S. *et al.* Reconstructing genetic histories and social organisation in Neolithic and Bronze Age Croatia. *Sci. Rep.* **11**, 16729 (2021).

46. Margaryan, A. *et al.* Population genomics of the Viking world. *Nature* **585**, 390–396 (2020).

47. Martiniano, R. *et al.* Genomic signals of migration and continuity in Britain before the Anglo-Saxons. *Nat. Commun.* **7**, 10326 (2016).

48. Mathieson, I. *et al.* The genomic history of southeastern Europe. *Nature* **555**, 197–203 (2018).

49. Mittnik, A. *et al.* Kinship-based social inequality in Bronze Age Europe. *Science* **366**, 731–734 (2019).

50. Morez, A. *et al.* Imputed genomes and haplotype-based analyses of the Picts of early medieval Scotland reveal fine-scale relatedness between Iron Age, early medieval and the modern people of the UK. *PLoS Genet.* **19**, e1010360 (2023).

51. Olalde, I. *et al.* The Beaker phenomenon and the genomic transformation of northwest Europe. *Nature* **555**, 190–196 (2018).

52. Rivollat, M. *et al.* Ancient genome-wide DNA from France highlights the complexity of interactions between Mesolithic hunter-gatherers and Neolithic farmers. *Sci Adv* **6**, eaaz5344 (2020).

53. Sánchez-Quinto, F. *et al.* Megalithic tombs in western and northern Neolithic Europe were linked to a kindred society. *Proc. Natl. Acad. Sci. U. S. A.* **116**, 9469–9474 (2019).

54. Schroeder, H. *et al.* Unraveling ancestry, kinship, and violence in a Late Neolithic mass grave. *Proc. Natl. Acad. Sci. U. S. A.* **116**, 10705–10710 (2019).

55. Seguin-Orlando, A. *et al.* Heterogeneous Hunter-Gatherer and Steppe-Related Ancestries in Late Neolithic and Bell Beaker Genomes from Present-Day France. *Curr. Biol.* **31**, 1072–1083.e10 (2021).

56. Žegarac, A. *et al.* Ancient genomes provide insights into family structure and the heredity of

social status in the early Bronze Age of southeastern Europe. *Sci. Rep.* **11**, 10072 (2021).

57. Danecek, P. *et al.* Twelve years of SAMtools and BCFtools. *Gigascience* **10**, (2021).

58. Weissensteiner, H. *et al.* HaploGrep 2: mitochondrial haplogroup classification in the era of high-throughput sequencing. *Nucleic Acids Res.* **44**, W58–63 (2016).

59. van Oven, M. & Kayser, M. Updated comprehensive phylogenetic tree of global human mitochondrial DNA variation. *Hum. Mutat.* **30**, E386–94 (2009).

60. Soares, P. *et al.* Correcting for purifying selection: an improved human mitochondrial molecular clock. *Am. J. Hum. Genet.* **84**, 740–759 (2009).

61. Nei, M. & Roychoudhury, A. K. Sampling variances of heterozygosity and genetic distance. *Genetics* **76**, 379–390 (1974).

62. Nei, M. & Tajima, F. DNA polymorphism detectable by restriction endonucleases. *Genetics* **97**, 145–163 (1981).

63. Yonova-Doing, E. *et al.* An atlas of mitochondrial DNA genotype-phenotype associations in the UK Biobank. *Nat. Genet.* **53**, 982–993 (2021).

64. Lott, M. T. *et al.* mtDNA variation and analysis using MITOMAP and MITOMASTER. *Curr. Protoc. Bioinformatics* 1.23.1–26 (2013).

65. Rebolledo-Jaramillo, B. *et al.* Maternal age effect and severe germ-line bottleneck in the inheritance of human mitochondrial DNA. *Proc. Natl. Acad. Sci. U. S. A.* **111**, 15474–15479 (2014).

66. Zaidi, A. A. *et al.* Bottleneck and selection in the germline and maternal age influence transmission of mitochondrial DNA in human pedigrees. *Proc. Natl. Acad. Sci. U. S. A.* **116**, 25172–25178 (2019).

67. McKenna, A. *et al.* The Genome Analysis Toolkit: a MapReduce framework for analyzing next-generation DNA sequencing data. *Genome Res.* **20**, 1297–1303 (2010).

68. Solé-Morata, N. *et al.* Analysis of the R1b-DF27 haplogroup shows that a large fraction of Iberian Y-chromosome lineages originated recently in situ. *Sci. Rep.* **7**, 7341 (2017).

69. Lucotte, G. The Major Y-Chromosome Haplogroup R1b-M269 in West-Europe, Subdivided by the Three SNPs S21/U106, S145/L21 and S28/U152, Shows a Clear Pattern of Geographic

Differentiation. *Advances in Anthropology* **05**, 22–30 (2015).

70. Schiffels, S. *et al.* Iron Age and Anglo-Saxon genomes from East England reveal British migration history. *Nat. Commun.* **7**, 10408 (2016).
71. Rootsi, S. *et al.* Phylogeography of Y-chromosome haplogroup I reveals distinct domains of prehistoric gene flow in europe. *Am. J. Hum. Genet.* **75**, 128–137 (2004).
72. Rootsi, S. *et al.* Distinguishing the co-ancestries of haplogroup G Y-chromosomes in the populations of Europe and the Caucasus. *Eur. J. Hum. Genet.* **20**, 1275–1282 (2012).
73. Horváth, C. Redefining pre-Indo-European language families of bronze age Western Europe: A study based on the synthesis of scientific evidence from archaeology, historical linguistics and genetics. *Eur. Sci. J.* (2019) doi:10.19044/esj.2019.v15n26p1.
74. Valverde, L. *et al.* New clues to the evolutionary history of the main European paternal lineage M269: dissection of the Y-SNP S116 in Atlantic Europe and Iberia. *Eur. J. Hum. Genet.* **24**, 437–441 (2016).
75. Rubinacci, S., Ribeiro, D. M., Hofmeister, R. J. & Delaneau, O. Efficient phasing and imputation of low-coverage sequencing data using large reference panels. *Nat. Genet.* **53**, 120–126 (2021).
76. Browning, B. L., Tian, X., Zhou, Y. & Browning, S. R. Fast two-stage phasing of large-scale sequence data. *Am. J. Hum. Genet.* **108**, 1880–1890 (2021).
77. Browning, B. L. & Browning, S. R. Improving the accuracy and efficiency of identity-by-descent detection in population data. *Genetics* **194**, 459–471 (2013).
78. Antonio, M. L. *et al.* Ancient Rome: A genetic crossroads of Europe and the Mediterranean. *Science* **366**, 708–714 (2019).
79. Gretzinger, J. *et al.* The Anglo-Saxon migration and the formation of the early English gene pool. *Nature* **610**, 112–119 (2022).
80. Jones, E. R. *et al.* The Neolithic Transition in the Baltic Was Not Driven by Admixture with Early European Farmers. *Curr. Biol.* **27**, 576–582 (2017).
81. Krzewińska, M. *et al.* Genomic and Strontium Isotope Variation Reveal Immigration Patterns in a Viking Age Town. *Curr. Biol.* **28**, 2730–2738.e10 (2018).
82. 1000 Genomes Project Consortium *et al.* A global reference for human genetic variation. *Nature*

526, 68–74 (2015).

83. Quinlan, A. R. & Hall, I. M. BEDTools: a flexible suite of utilities for comparing genomic features. *Bioinformatics* **26**, 841–842 (2010).

84. Goudet, J., Kay, T. & Weir, B. S. How to estimate kinship. *Mol. Ecol.* **27**, 4121–4135 (2018).

85. Ramstetter, M. D. *et al.* Benchmarking Relatedness Inference Methods with Genome-Wide Data from Thousands of Relatives. *Genetics* **207**, 75–82 (2017).

86. Chang, C. C. *et al.* Second-generation PLINK: rising to the challenge of larger and richer datasets. *Gigascience* **4**, 7 (2015).

87. Manichaikul, A. *et al.* Robust relationship inference in genome-wide association studies. *Bioinformatics* **26**, 2867–2873 (2010).

88. Caballero, M. *et al.* Crossover interference and sex-specific genetic maps shape identical by descent sharing in close relatives. *PLoS Genet.* **15**, e1007979 (2019).

89. Bhérer, C., Campbell, C. L. & Auton, A. Refined genetic maps reveal sexual dimorphism in human meiotic recombination at multiple scales. *Nat. Commun.* **8**, 14994 (2017).

90. Campbell, C. L., Furlotte, N. A., Eriksson, N., Hinds, D. & Auton, A. Escape from crossover interference increases with maternal age. *Nat. Commun.* **6**, 6260 (2015).

91. Ramstetter, M. D. *et al.* Inferring Identical-by-Descent Sharing of Sample Ancestors Promotes High-Resolution Relative Detection. *Am. J. Hum. Genet.* **103**, 30–44 (2018).

92. Ringbauer, H., Novembre, J. & Steinrücken, M. Parental relatedness through time revealed by runs of homozygosity in ancient DNA. *Nat. Commun.* **12**, 5425 (2021).

93. Mathieson, I. *et al.* Genome-wide patterns of selection in 230 ancient Eurasians. *Nature* **528**, 499–503 (2015).

94. Traag, V. A., Waltman, L. & van Eck, N. J. From Louvain to Leiden: guaranteeing well-connected communities. *Sci. Rep.* **9**, 5233 (2019).

95. Leslie, S. *et al.* The fine-scale genetic structure of the British population. *Nature* **519**, 309–314 (2015).

96. International Multiple Sclerosis Genetics Consortium *et al.* Genetic risk and a primary role for cell-mediated immune mechanisms in multiple sclerosis. *Nature* **476**, 214–219 (2011).

97. Patterson, N., Price, A. L. & Reich, D. Population structure and eigenanalysis. *PLoS Genet.* **2**, e190 (2006).
98. Gamba, C. *et al.* Genome flux and stasis in a five millennium transect of European prehistory. *Nat. Commun.* **5**, 5257 (2014).
99. Allentoft, M. E. *et al.* Population genomics of Bronze Age Eurasia. *Nature* **522**, 167–172 (2015).
100. Damgaard, P. de B. *et al.* 137 ancient human genomes from across the Eurasian steppes. *Nature* **557**, 369–374 (2018).
101. Brunel, S. *et al.* Ancient genomes from present-day France unveil 7,000 years of its demographic history. *Proc. Natl. Acad. Sci. U. S. A.* **117**, 12791–12798 (2020).
102. Cassidy, L. M. *et al.* A dynastic elite in monumental Neolithic society. *Nature* **582**, 384–388 (2020).
103. Broushaki, F. *et al.* Early Neolithic genomes from the eastern Fertile Crescent. *Science* **353**, 499–503 (2016).
104. Gamba, C. *et al.* Genome flux and stasis in a five millennium transect of European prehistory. *Nat. Commun.* **5**, 5257 (2014).
105. Lazaridis, I. *et al.* Ancient human genomes suggest three ancestral populations for present-day Europeans. *Nature* **513**, 409–413 (2014).
106. González-Fortes, G. *et al.* Paleogenomic Evidence for Multi-generational Mixing between Neolithic Farmers and Mesolithic Hunter-Gatherers in the Lower Danube Basin. *Curr. Biol.* **27**, 1801–1810.e10 (2017).
107. Yaka, R. *et al.* Variable kinship patterns in Neolithic Anatolia revealed by ancient genomes. *Curr. Biol.* **31**, 2455–2468.e18 (2021).
108. Mallick, S. *et al.* The Simons Genome Diversity Project: 300 genomes from 142 diverse populations. *Nature* **538**, 201–206 (2016).
109. Lawson, D. J., Hellenthal, G., Myers, S. & Falush, D. Inference of population structure using dense haplotype data. *PLoS Genet.* **8**, e1002453 (2012).
110. Hellenthal, G. *et al.* A genetic atlas of human admixture history. *Science* **343**, 747–751 (2014).
111. Chacón-Duque, J.-C. *et al.* Latin Americans show wide-spread Converso ancestry and imprint of

local Native ancestry on physical appearance. *Nat. Commun.* **9**, 5388 (2018).

112. Delaneau, O., Zagury, J.-F. & Marchini, J. Improved whole-chromosome phasing for disease and population genetic studies. *Nat. Methods* **10**, 5–6 (2013).

113. Schiffels, S. & Sayer, D. Investigating Anglo-Saxon migration history with ancient and modern DNA. in *Migration and Integration form Prehisory to the Middle Ages* vol. 17 11 (Tagungen Des Landesmuseums Für Vorgeschichte Halle, Saale, 2017).

114. Taylor, A., Weale, A. & Ford, S. *Bronze Age, Iron Age and Roman Landscapes of the Costal Plain, and a Late Iron Age Warrior Burial at North Bersted, Bognor Regis, West Sussex.* (Thames Valley Archaeological Services Limited, Reading, 2014).
