# Supplementary material for: Continental influx and pervasive matrilocality in Iron Age Britain
Source: Nature. 2025 Jan 15;637(8048):1136–42. doi: 10.1038/s41586-024-08409-6 (PMC11779635; doi:10.1038/s41586-024-08409-6)
Supplement: Supplementary file 2 — Reporting Summary [file 41586_2024_8409_MOESM2_ESM.pdf]

nature portfolio

Corresponding author(s): Lara M Cassidy

Last updated by author(s): 21/10/2024

Reporting Summary

Nature Portfolio wishes to improve the reproducibility of the work that we publish. This form provides structure for consistency and transparency in reporting. For further information on Nature Portfolio policies, see our Editorial Policies and the Editorial Policy Checklist.

Statistics

For all statistical analyses, confirm that the following items are present in the figure legend, table legend, main text, or Methods section.

n/a	Confirmed	
☐	☒	The exact sample size (*n*) for each experimental group/condition, given as a discrete number and unit of measurement
☒	☐	A statement on whether measurements were taken from distinct samples or whether the same sample was measured repeatedly
☐	☒	The statistical test(s) used AND whether they are one- or two-sided<br>*Only common tests should be described solely by name; describe more complex techniques in the Methods section.*
☒	☐	A description of all covariates tested
☒	☐	A description of any assumptions or corrections, such as tests of normality and adjustment for multiple comparisons
☒	☐	A full description of the statistical parameters including central tendency (e.g. means) or other basic estimates (e.g. regression coefficient) AND variation (e.g. standard deviation) or associated estimates of uncertainty (e.g. confidence intervals)
☒	☐	For null hypothesis testing, the test statistic (e.g. *F*, *t*, *r*) with confidence intervals, effect sizes, degrees of freedom and *P* value noted<br>*Give P values as exact values whenever suitable.*
☐	☒	For Bayesian analysis, information on the choice of priors and Markov chain Monte Carlo settings
☒	☐	For hierarchical and complex designs, identification of the appropriate level for tests and full reporting of outcomes
☒	☐	Estimates of effect sizes (e.g. Cohen's *d*, Pearson's *r*), indicating how they were calculated

Our web collection on statistics for biologists contains articles on many of the points above.

Software and code

Policy information about availability of computer code

Data collection	Sequencing data was generated on Illumina platforms. This data was coanalysed with publicly available sequence data downloaded from ENA using wget (GNU).
Data analysis	Details on what each software was used for can be found in the methods section. Software names and versions are provided in a list below.<br>FASTQC v0.11.5<br>cutadapt v1.9.1<br>AdapterRemoval v2.3.1<br>BWA v0.7.5a-r405<br>SAMtools v1.7<br>GATK v3.7.0<br>Picard Tools v2.0.1<br>BCFtools v1.10.2<br>Haplogrep2 v2.2.9<br>smartpca v16000 (EIGENSOFT)<br>ADMIXTOOLS2 v2.0.4<br>GLIMPSE1 v1.1.0<br>Beagle5 v05May22.33a<br>refinedIBD v17Jan20.102<br>leidenAlg v1.1.1<br>phangorn v2.11.1<br>SHAPEIT2 v2.r837

ChromoPainter v2
SOURCEFIND v2
fineSTRUCTURE v2
ped-sim

For manuscripts utilizing custom algorithms or software that are central to the research but not yet described in published literature, software must be made available to editors and reviewers. We strongly encourage code deposition in a community repository (e.g. GitHub). See the Nature Portfolio guidelines for submitting code & software for further information.

Data

Policy information about availability of data

All manuscripts must include a data availability statement. This statement should provide the following information, where applicable:

- Accession codes, unique identifiers, or web links for publicly available datasets
- A description of any restrictions on data availability
- For clinical datasets or third party data, please ensure that the statement adheres to our policy

Aligned sequence reads are available through the European Nucleotide Archive under accession number PRJEB81465. Other relevant data are available from the corresponding authors upon reasonable request.

Research involving human participants, their data, or biological material

Policy information about studies with human participants or human data. See also policy information about sex, gender (identity/presentation), and sexual orientation and race, ethnicity and racism.

Reporting on sex and gender	Samples were defined as male or female based on read coverage across the sex chromosomes. No sex chromosome aneuploidies were observed. Comparisons were made between the male and female populations buried at Winterborne Kingston to draw inferences about kinship and marriage customs. When discussing these customs and other cultural phenomena we use the terms men and women.
Reporting on race, ethnicity, or other socially relevant groupings	We do not bin samples by the socially constructed categories of race or ethnicity. We group samples by geographical region or genetic cluster (defined based on haplotypic data).
Population characteristics	All human samples are archaeological in nature. An osteological assessment of age-at-death, pathologies and trauma was carried out.
Recruitment	N/A
Ethics oversight	N/A

Note that full information on the approval of the study protocol must also be provided in the manuscript.

Field-specific reporting

Please select the one below that is the best fit for your research. If you are not sure, read the appropriate sections before making your selection.

☒ Life sciences ☐ Behavioural & social sciences ☐ Ecological, evolutionary & environmental sciences

For a reference copy of the document with all sections, see nature.com/documents/nr-reporting-summary-flat.pdf

Life sciences study design

All studies must disclose on these points even when the disclosure is negative.

Sample size	We exhaustively sampled all burials excavated at Winterborne Kingston. These were analysed with all publicly available data from the Iron Age of northwestern Europe.
Data exclusions	Data was excluded from certain analyses based on:<br><br>1. genomic coverage and genotype missingness<br>2. temporal range<br>3. geographic range<br>4. outlying genetic ancestry<br><br>Rationale for these exclusions is given in detail in the Methods section and Supplementary Information.
Replication	N/A

Randomization	N/A
Blinding	N/A

Reporting for specific materials, systems and methods

We require information from authors about some types of materials, experimental systems and methods used in many studies. Here, indicate whether each material, system or method listed is relevant to your study. If you are not sure if a list item applies to your research, read the appropriate section before selecting a response.

Materials & experimental systems

n/a	Involved in the study
☒	☐ Antibodies
☒	☐ Eukaryotic cell lines
☐	☒ Palaeontology and archaeology
☒	☐ Animals and other organisms
☒	☐ Clinical data
☒	☐ Dual use research of concern
☒	☐ Plants

Methods

n/a	Involved in the study
☒	☐ ChIP-seq
☒	☐ Flow cytometry
☒	☐ MRI-based neuroimaging

Palaeontology and Archaeology

Specimen provenance	All samples were obtained from the Department of Archaeology and Anthropology, Bournemouth University, United Kingdom. This museum collection is curated by Gabrielle Delbarre, a co-author on this study, who provided the necessary permissions to sample these specimens for ancient DNA and radiocarbon dating.
Specimen deposition	Residues from aDNA sampling have been returned to the collection at Bournemouth University.
Dating methods	New dates were generated at CIRAM, Bordeaux, France and Chronocentre, QUB, UK. Details of each laboratory's protocol are available from their websites. Dates were calibrated using CALIB 8.2 and the IntCal20 curve.

☒ Tick this box to confirm that the raw and calibrated dates are available in the paper or in Supplementary Information.

Ethics oversight	Ethical approval was obtained from the School of Natural Sciences Research Ethics Committee, Trinity College Dublin. All samples are over 1400 years old and thus have no close genetic relationships to living persons. These specimens do not come from a sensitive context that has special social or political significance to some people alive today. We followed established ethical guidelines within the field of ancient DNA. This includes drawing up a detailed research plan prior to initiating the project, minimizing damage to human remains while sampling and depositing sequence data to a publicly accessible online repository.

Note that full information on the approval of the study protocol must also be provided in the manuscript.

Plants

Seed stocks	N/A
Novel plant genotypes	N/A
Authentication	N/A
